# 1-Iodoglycal: A Versatile Intermediate for the Synthesis of d-Glyco Amides and Esters Employing Carbonylative Cross-Coupling Reaction

**DOI:** 10.1021/acsomega.4c02645

**Published:** 2024-07-11

**Authors:** Milene M. Hornink, Monica F. Z. J. Toledo, Daniel C. Pimenta, Caio Paschoalin, Pamela M. Silva, Giuseppe E. Figlino, Eurípedes Aguiar, Gustavo Cervi, Francisco W. M. Ribeiro, Thiago Carita Correra, Angélique Ferry, Hélio A. Stefani

**Affiliations:** † Departamento de Farmácia, Faculdade de Ciências Farmacêuticas, Universidade de São Paulo, São Paulo 05508-900, SP, Brasil; ‡ Instituto Butantan, São Paulo 05503-900, SP, Brasil; § Instituto de Química, Universidade de São Paulo, São Paulo 05513-900, SP, Brasil; ∥ Centro Universitário São Camilo, São Paulo 04263-200, SP, Brasil; ⊥ Université Paris-Saclay, CNRS, BioCIS, 91400 Orsay, France; # BioCIS, CNRS, CY Cergy-Paris Université, 5 mail Gay-Lussac, 95000 Cergy-Pontoise cedex, France; ∇ Institut Universitaire de France (IUF), Paris 75005, France

## Abstract

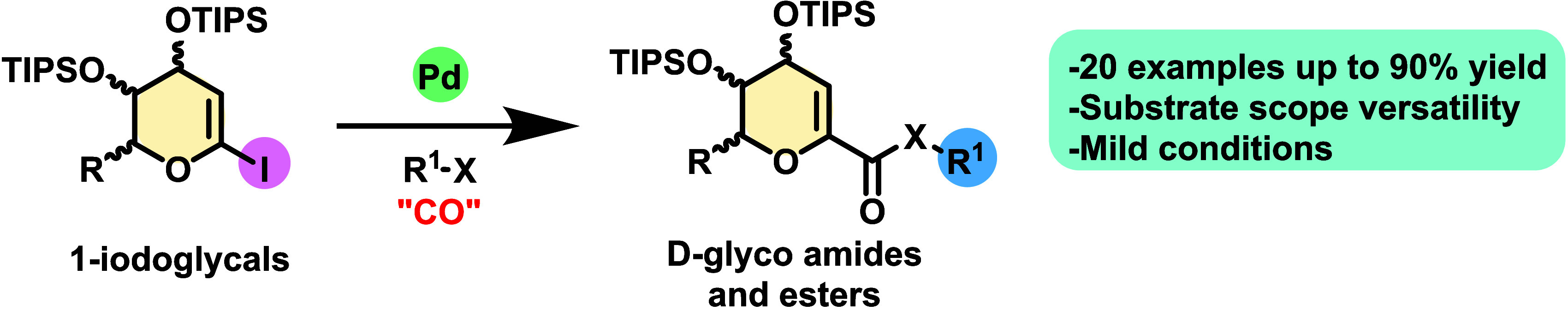

In this study, we present the development of two catalytic processes: a Pd-PEPPSI-catalyzed aminocarbonylation and a Pd(OAc)_2_-Xantphos-catalyzed alkoxycarbonylation of d-glycals, utilizing carbonylative cross-coupling reactions. We explored successfully various types of aromatic amines, as well as alkyl amines and amino acids, to synthesize new d-glycal amides. However, we observed limitations in the reactivity of alkyl and heteroaromatic amines. The processes enabled the synthesis of 20 novel C1-branched glycoamides and 7 new d-gluco esters.

## Introduction

The amide functional group is widely present in small, complex molecules, whether they are synthetic or natural, and it is frequently found in biological active molecules.^1^ The construction of amide bonds is one of the most important and common transformations in organic synthesis.^2^ The investigation of methods for catalytic and sustainable amide or peptide formation was defined in 2018 as a priority in the 10 Key Green Chemistry Research Areas by the ACS Green Chemistry Institute Pharmaceutical Roundtable (GCIPR).^3^

**Figure 1:**
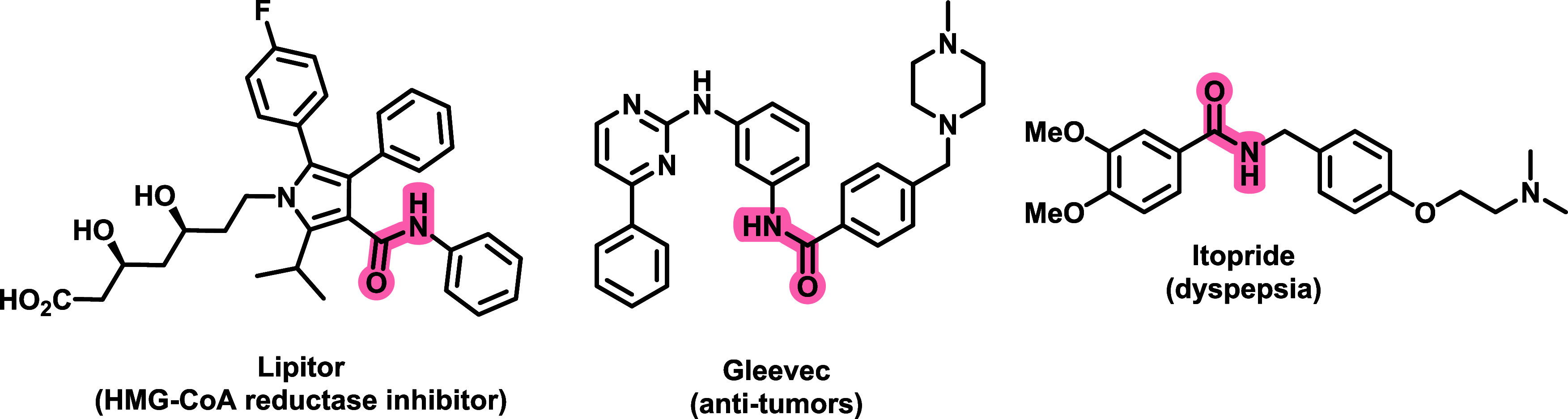
Chemical structures of amide-containing marketed drugs.

There is a great number of drugs containing this pattern, such as, Atorvastatin (common trade name: Lipitor) used for the prevention of cardiovascular disease,^4^ Imatinib (common trade name: Gleevec), which is an oral targeted therapy medication used to treat cancer,^5^ and Itopride (common trade name: Ganaton), indicated for the treatment of functional dyspepsia and other gastrointestinal diseases^6^ (Figure 1).

Traditional methods for forming amide bonds usually entail nucleophilic acyl substitutions.^7^ These reactions require prior activation of the carboxylic acid group, typically as acyl chlorides or reactive anhydrides and esters.^8^ It has been estimated that the “acylation of amine” accounts for 16% of the reactions commonly used in pharmaceutical synthesis.^9^ Transition-metal-catalyzed carbonylations, on the other hand, utilize CO as the primary source of the carbonyl moiety, thereby circumventing the use of hazardous chemicals.^10^ Nowadays, a variety of CO surrogates,^11^ such as metal carbonyls,^12^ oxalic acid,^13^ chloroform,^14^ or silacarboxylic acids,^15^ are available for performing aminocarbonylation reactions.

**Scheme 1:**
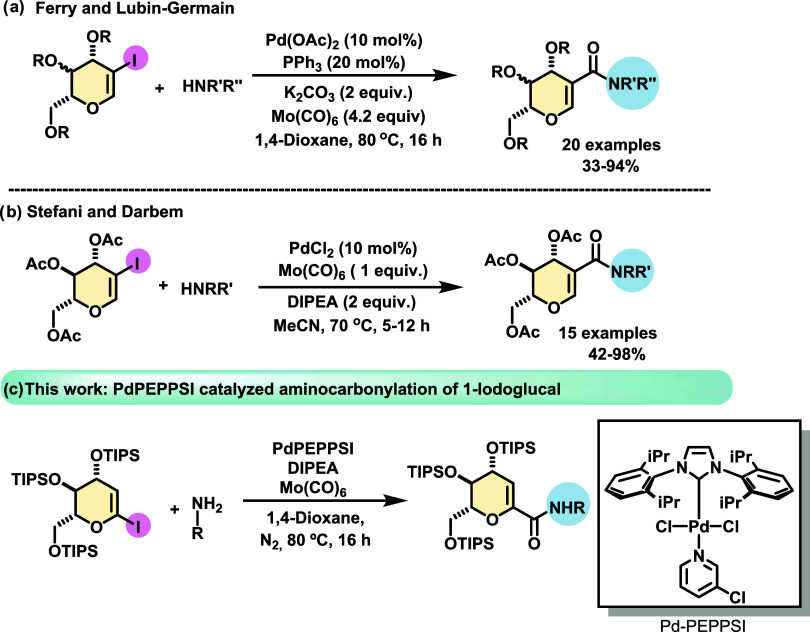
Previous Aminocarbonylations of 2-Iodoglycals Reported by (a) the Ferry Group, (b) the Stefani Group, and (c) This Work

A large number of studies dealing with the development of transition-metal-catalyzed aminocarbonylation reactions have already been performed using aromatic and heteroaromatic halides.^16^ There are only two previous reports that applied the aminocarbonylation reaction on sugar derivatives, using a solid source of CO, such as Mo(CO)_6_. The first systematic study was described by Ferry et al.,^17^ whose approach used diverse protected 2-iodoglycals with aromatic, and alkyl amines as well as sulfonamide or amino esters. Different protecting groups, such as benzyl, acetyl or isopropylidene, were evaluated to verify the extent of the reaction (Scheme 1a).

The second study, published by Stefani and co-workers,^18^ described the synthesis of amidoglucal compounds via the aminocarbonylation cross-coupling reaction between 2-iodoglucal and various amines. Aromatic and heteroaromatic compounds, alkyl amines, amino esters, and ureas were used (Scheme 1b).

Current carbonylative coupling methods for amide synthesis mainly use aromatic derivatives. As stated above, the aminocarbonylation of more functionalized molecules such as glycals is poorly investigated, and the methodologies developed to date give access only to C2-branched sugars. Considering the vital role of sugars as drugs and as enhancers of bioavailability and solubility, herein we report a protocol for the aminocarbonylation of 1-iodoglycals to access new C1-glycoamides (Scheme 1c).

## Results and Discussion

**Scheme 2:**
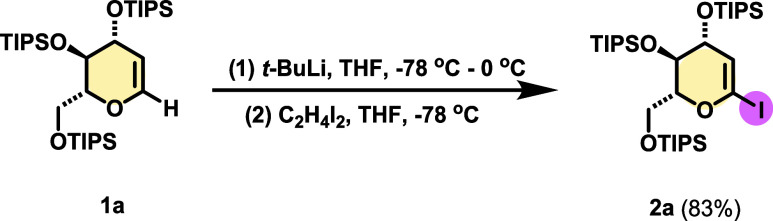
Starting Material Preparation

The model substrate, per-silylated 1-iodoglucal (**2b**), was synthesized according to literature procedures.^19^ Initially, in order to introduce iodine at position 1, it was necessary to protect the free hydroxyl groups of commercial d-glucal using triisopropylsilane chloride (TIPSCl) and imidazole in *N*,*N*-dimethylformamide (DMF). Then, treatment with *t*-BuLi in tetrahydrofuran (THF) at −78 °C, followed by the addition of diiodoethane (C_2_H_4_I_2_) to the silylated glucal vinyl anion, led to the desired 1-iodoglucal **2b** (Scheme 2).

The first set of conditions were chosen to evaluate the possible formation of the coupling product by fixing the coupling partners Mo(CO)_6_ (1 equiv) as a solid and easy-to-handle “CO” source, *N*,*N*-diisopropylethylamine (DIPEA, 2 equiv) as the base, 1,4-dioxane as the solvent, and aniline as the partner (1.2 equiv). Initially, we conducted the aminocarbonylation reaction using PdCl_2_ as the sole catalyst without a ligand. As depicted in Table 1, entry 1, these conditions yielded an encouraging 20% NMR yield (18% isolated) of the desired product **3a**.

**Table 1:**
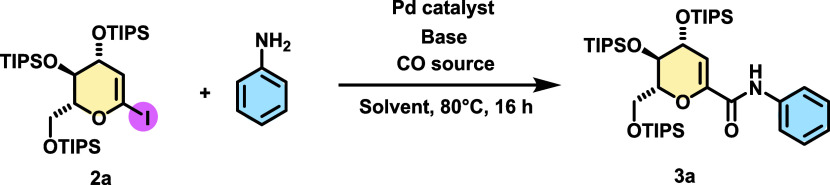
Optimization of the Aminocarbonylation Reaction Conditions

entry	catalyst/ligand	base	solvent	“CO” source	yield (%)^a^**3a**
Catalyst/Ligand Screening					
1	PdCl_2_	DIPEA	1,4-dioxane	Mo(CO)_6_	20 (18)
2	PdBr_2_	DIPEA	1,4-dioxane	Mo(CO)_6_	21
3	Pd(OAc)_2_	DIPEA	1,4-dioxane	Mo(CO)_6_	28
4	Pd(acac)_2_	DIPEA	1,4-dioxane	Mo(CO)_6_	21
5	Pd(PhCN)_2_Cl_2_	DIPEA	1,4-dioxane	Mo(CO)_6_	26
6	Pd(dba)_2_	DIPEA	1,4-dioxane	Mo(CO)_6_	29
7	Pd_2_(dba)_3_	DIPEA	1,4-dioxane	Mo(CO)_6_	28
8	Pd(OAc)_2_/PPh_3_ (20 mol %)	DIPEA	1,4-dioxane	Mo(CO)_6_	37
9	Pd(OAc)_2_/XantPhos (10 mol %)	DIPEA	1,4-dioxane	Mo(CO)_6_	90
10	Pd-PEPPSI	DIPEA	1,4-dioxane	Mo(CO)_6_	97 (90)
11		DIPEA	1,4-dioxane	Mo(CO)_6_	N.R
12	Pd-PEPPSI	DIPEA	1,4-dioxane	Mo(CO)_6_	N.R^b^
Base Screening					
13	Pd-PEPPSI	TEA	1,4-dioxane	Mo(CO)_6_	28
14	Pd-PEPPSI	K_2_CO_3_	1,4-dioxane	Mo(CO)_6_	19
15	Pd-PEPPSI	*t*BuOK	1,4-dioxane	Mo(CO)_6_	56
Solvent Screening					
16	Pd-PEPPSI	DIPEA	toluene	Mo(CO)_6_	16
17	Pd-PEPPSI	DIPEA	acetonitrile	Mo(CO)_6_	49 (55)
“CO” Source Screening					
18	Pd-PEPPSI	DIPEA	1,4-dioxane	Fe_2_(CO)_9_	N.R
**19**	Pd-PEPPSI	DIPEA	1,4-dioxane	Co_2_(CO)_8_	N.R
20	Pd-PEPPSI	DIPEA	1,4-dioxane	CO balloon	N.R

a Yield determined by ^1^H NMR using trichloroethylene as an internal standard.

b Reaction under US irradiation, 60 min, ultrasound bath. N.R. = No Reaction. Isolated yields in parentheses.

Next, we carried out the reaction with several palladium catalysts, such as PdBr_2_, Pd(OAc)_2_, Pd(acac)_2_, Pd(PhCN)_2_Cl_2_, Pd(dba)_2_, and Pd_2_(dba)_3_, without the presence of ligands.^20^ As shown in Table 1, entries 2–7, the yields were very similar, ranging from 20 to 29%, after a 16 h reaction. We chose Pd(OAc)_2_ as the palladium source and evaluated the addition of some phosphine ligands. As shown in Table 1, entry 8, the use of triphenylphosphine (20 mol %) improved the yield to 37%; meanwhile, Xantphos (Table 1, entry 9) provided 90% NMR yield of **3a**. Then, we decided to evaluate the activity of Pd-PEPPSI, and as depicted in Table 1, entry 10. This catalyst proved to provide the best yield with a 97% NMR yield (90% of isolated yield of the product). Considering the disadvantages of using phosphine ligands, such as air and moisture sensitivity, high cost, and difficult purification from the final product,^21^ and regarding some previous reports on phosphine-free aminocarbonylations,^22^ we decided to investigate the reaction with the phosphine-free conditions afforded by the Pd-PEPPSI catalyst. When the reaction was performed in the absence of the Pd-PEPPSI catalyst, the formation of the aminocarbonylation product was not observed, as shown in Table 1, entry 11. An attempt to perform the reaction using ultrasound irradiation also did not prove to be favorable for obtaining the product, as shown in Table 1, entry 12.

The use of a different organic base, such as triethylamine (TEA), afforded a low yield (only 28%) (Table 1, entry 13). When inorganic bases such as K_2_CO_3_ were evaluated, only 19% yield of **3a** was obtained, as shown in Table 1, entry 14. *t*BuOK provided moderate yield of the desired product (Table 1, entry 15), probably due to the significant steric hindrance of *t*Bu group. Next, we turned our attention to the reaction solvent. Toluene afforded a poor yield of the product, while acetonitrile provided a moderate yield, as shown in Table 1, entries 16 and 17, respectively. We also performed a screening of CO sources other than Mo(CO)_6_, such as Fe(CO)_9_, Co_2_(CO)_8_, or CO gas (atmospheric pressure), as depicted in Table 1 entries 18–20. None of them provided the desired product, the ^1^H NMR of the crude reaction mixture showed only the starting 1-iodoglycal **2a**. The lower efficiency of other CO sources in aminocarbonylation reactions have been already noted previously in the literature.^23^

With optimized conditions established, which included 1-iodoglycal (1.0 equiv), amine (1.2 equiv), Pd-PEPPSI (10 mol %), DIPEA (2.0 equiv), Mo(CO)_6_ (1.0 equiv), and 1,4-dioxane at 80 °C for 16 h, we investigated the scope of the aminocarbonylation coupling reaction. Various aromatic and heteroaromatic compounds, primary and secondary amines, alkyl amines, and amino esters were examined.

The reaction with our model amine, aniline, led to the formation of the C1-amidoglycal product **3a** with an excellent isolated yield of 90%, as shown in Table 2. Next, we performed the reaction with a series of methoxy-substituted anilines. The *ortho* and *para*-methoxy anilines afforded the products **3b** and **3d** in low yields, respectively 38 and 45% while the *meta*-methoxy aniline provided amide **3c** in a good 59% yield. Similar yields were observed for compound **3e**, containing a *para*-methyl group on the benzene ring, and for the sterically hindered 2,4,6-trimethylaniline **3f** (36 and 46%, respectively).

**Table 2:**
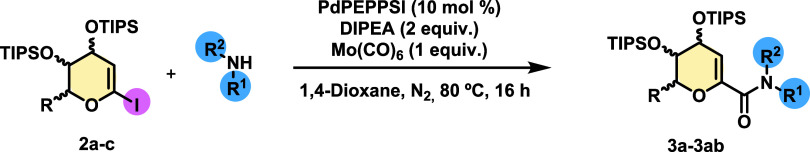

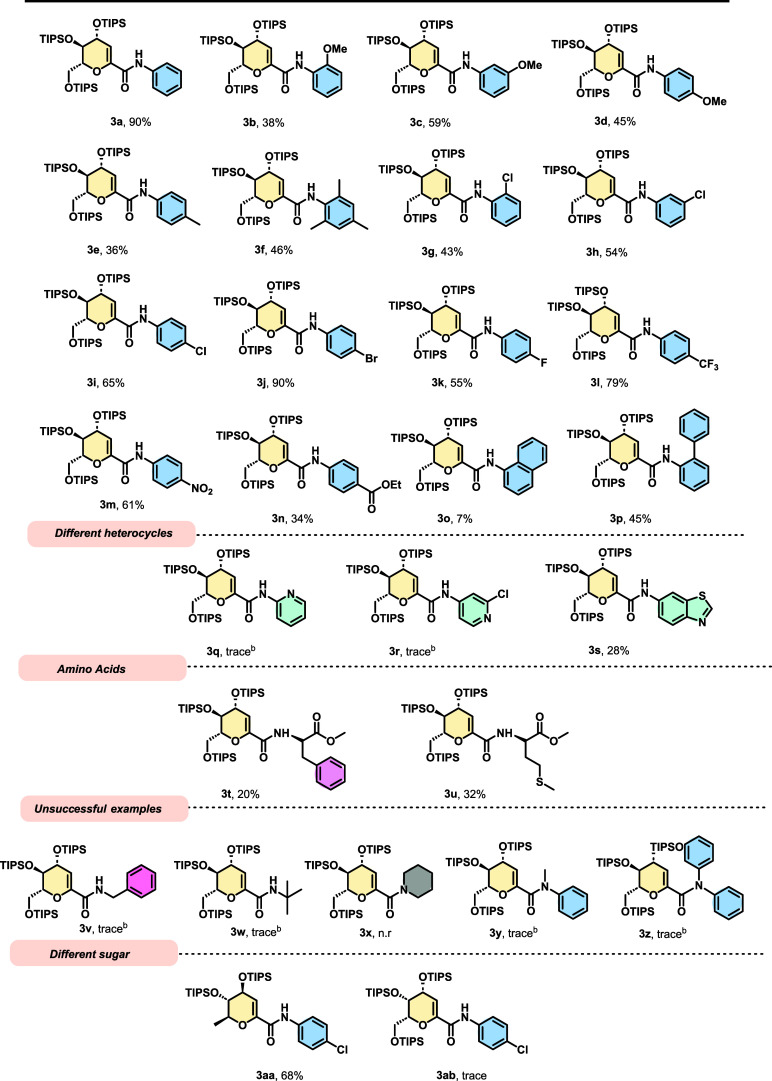
Substrate Scope for Amines^a^

a Reaction conditions: **2a**–**c** (0.1 mmol), DIPEA (0.2 mmol), amine (0.12 mmol), Mo(CO)_6_ (0.1 mmol), Pd-PEPPSI (10 mol %), 1,4 dioxane (0.8 mL), 80 °C, 16 h.

b Traces of products were determined by ^1^H NMR of the crude.

The reaction with *ortho*, *meta*, and *para* chloroanilines provided slightly improved yields when compared to the electron donating anilines (**3g**, **3h**, **3i**, Table 2). Other halogen-containing analogs, such as *p*-bromoaniline and *p*-fluoroaniline, provide the aminocarbonylation products in excellent to good yields, (**3j** and **3k** respectively). The electron withdrawing groups, such as *p*-trifluoromethyl and *p*-nitro groups, also afforded the amide products **3l** and **3m** in high and good yields (79 and 61%, respectively).

As an amine partner, 1-naphthylamine affords the product **3o** in a poor isolated yield of only 7%, while [1,1′-biphenyl]-2-amine, used as a hindered example, allowed compound **3p** to be obtained with a 45% yield. Attempts to prepare d-gluco amides using heteroaromatic amines were also tested. The use of aminopyridine derivatives **3q** and **3r** yielded only trace amounts of the desired products, as observed in the ^1^H NMR analysis of the crude extracted mixture, while heterocyclic 6-aminobenzothiazole provided **3s** with a low yield (28%).

Amino esters were also tested to verify the range of the aminocarbonylation reaction. The methyl-esters of phenylalanine and methionine were applied as coupling partners and provided products **3t** and **3u** in yields of 20 and 32%, respectively (Table 2).

Alkyl amines, such as benzylamine and *t*-butylamine, provided traces of the coupling products **3v** and **3w**, and secondary amines, such as piperidine, *N*-methylaniline, and diphenylamine, also showed no reaction or trace amounts of the coupling products, **3x**, **3y**, and **3z** (Table 2).

Besides the amine, other 1-iodoglycals were engaged in the aminocarbonylation conditions, such as 1-iodo-l-rhamnal and 1-iodo-d-galactal, synthesized according to the procedures outlined in the literature.^24^ As shown in Table 2, Compound **3aa** was isolated with a yield similar to those obtained with d-glucal; on the other hand, the product of the carbonylative coupling reaction with d-galactal was observed only in traces (**3ab**), probably due to the high instability of the starting compound.

Taking advantage of the reaction conditions developed to obtain d-gluco amides, the possibility of obtaining the corresponding d-gluco esters was envisioned.^18^ Initially, we applied the same reaction conditions optimized for the gluco-amides. In this first attempt, we engaged 1-iodoglucal and phenol, in the presence of the Pd-PEPPSI catalyst, obtaining only 23% of the isolated compound **4a** (Table 3). Next, we evaluated Pd(OAc)_2_ as the sole catalyst in the absence of ligands, and a low yield of 21% was achieved.

**Table 3:**
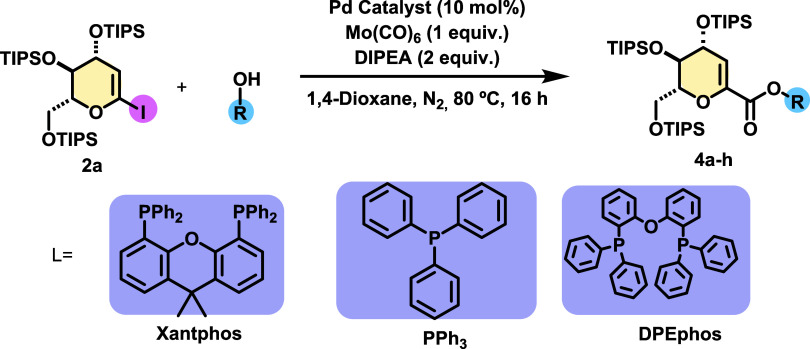

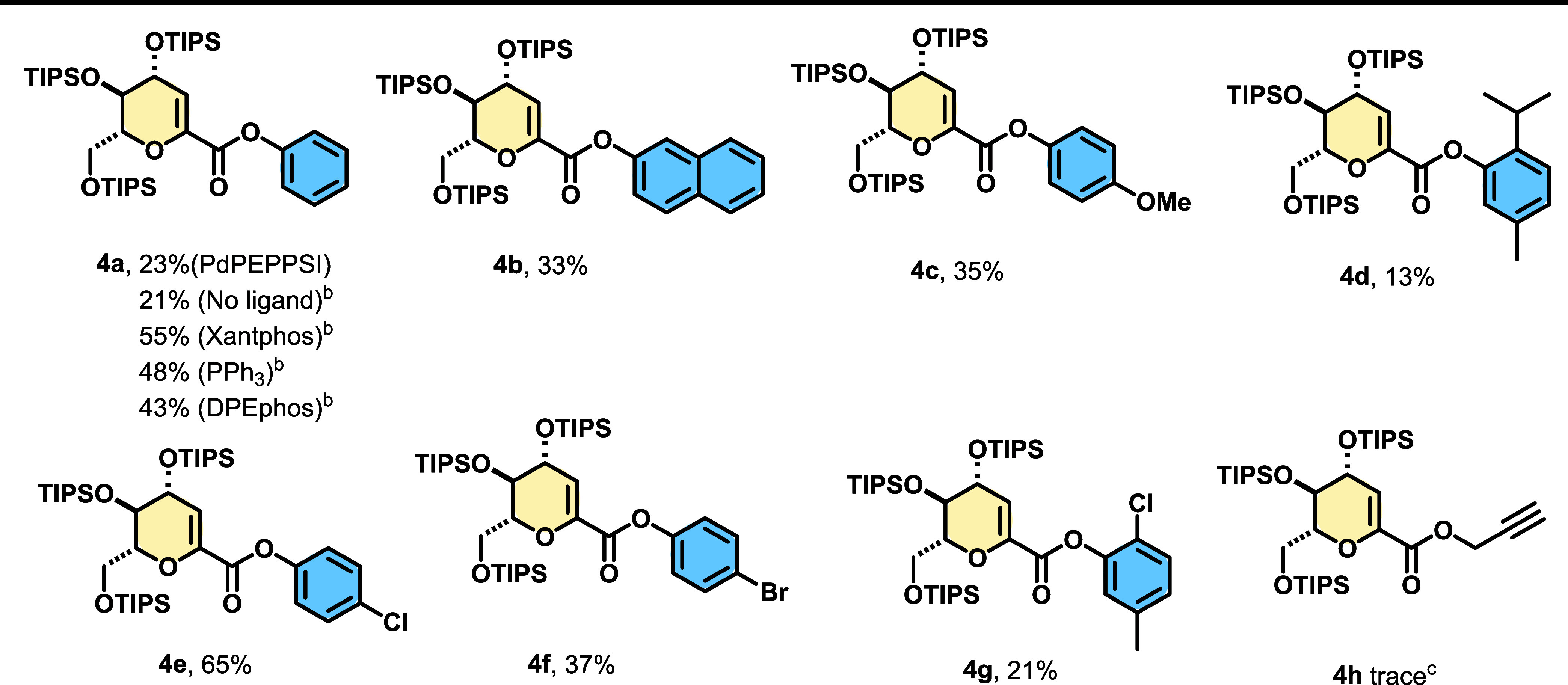
Substrate Scope for Alcohols^a^

a Reaction conditions: **2a** (0.1 mmol), DIPEA (0.2 mmol), alcohol (0.12 mmol), Mo(CO)_6_ (0.1 mmol), Pd(OAc)_2_ (10 mol %), Xantphos (10 mol %) 1,4-dioxane (0.8 mL), 80 °C, 16 h.

b Pd(OAc)_2_ as catalyst.

c Traces of products were determined by ^1^H NMR of the crude.

In the hope of improving the reaction yield, three phosphines were surveyed as ligand: Xantphos, which gave a yield of 55%, as well as triphenylphosphine and DPEPhos, which provided yields of 48 and 43%, respectively (Table 3). With these results in hand, we decided to perform alkoxy carbonylation, employing Pd(OAc)_2_ as the catalyst and Xantphos as ligand, to evaluate the substrate scope with different alcohol partners. Reaction with naphthalen-2-ol gave a 35% yield (**4b**), and alcohols with electron donating groups such as 4-methoxyphenol (**4c**) and 2-isopropyl-5-methylphenol (**4d**) afforded the alkoxycarbonylation products in yields of 35 and 13%, respectively (Table 3). Aromatic alcohols bearing halogen substituents on the benzene ring, such as *p*-chlorine or *p*-bromine, were also applied, and the desired products **4e** and **4f** were isolated with 65 and 37% yields, respectively. When the 2-chloro-5-methylphenol (**4g**) was applied, a low yield of 21% of the corresponding product was obtained. Reaction with propargyl alcohol (**4h**) resulted in only traces of the coupling product (Table 3).

**Scheme 3:**
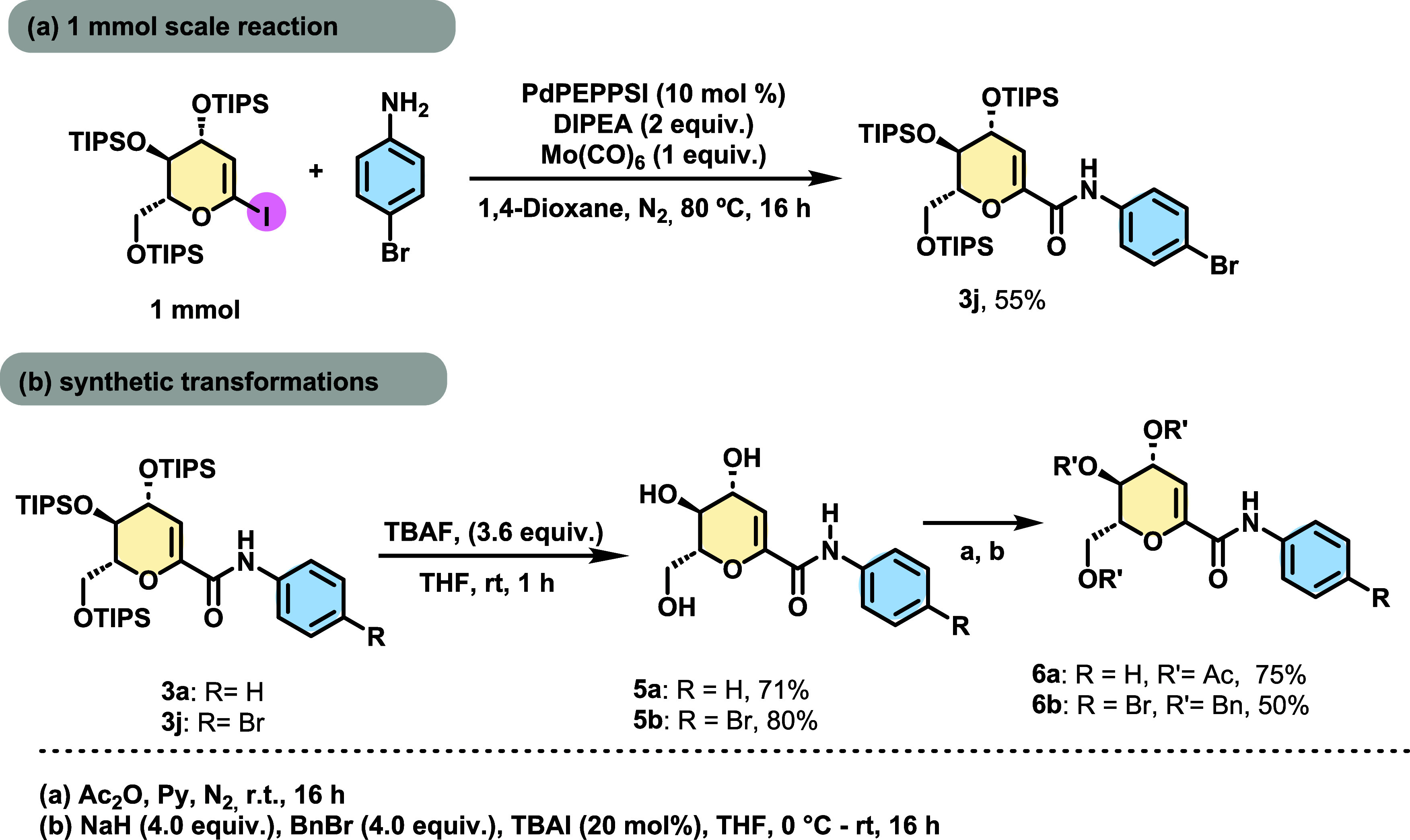
Gram-scale Synthesis and Further Synthetic Transformations

To demonstrate the potential applications of the obtained products, some synthetic transformations were performed. First, we carried out a reaction on a 10-times scale (1 mmol) and obtained the coupling product with a 55% yield (Scheme 3a). Then we performed the deprotection of two compounds with tetra-*n*-butylammonium fluoride (TBAF) in THF as the solvent; the hydroxylated amides were obtained in 71% (**5a**) and 80% (**5b**) yields, respectively. (Scheme 3b).^25^

Subsequently, the deprotected glucal **5a** underwent a protection reaction with acetic anhydride and pyridine, resulting in the triacetylated product (**6a**) with a yield of 75%. Similarly, compound **5b** was protected with benzyl groups, yielding product **6b** with a 50% yield (Scheme 3b).

**Scheme 4:**
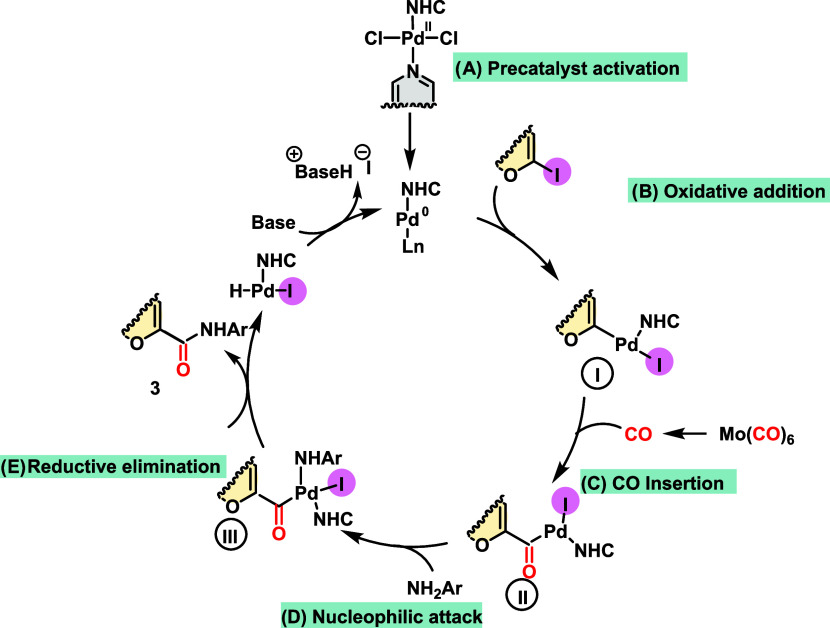
Proposed Reaction Mechanism

Based on these experimental results and previous reports of similar transformations,^26^ a reaction mechanism was proposed. First, reduction of the Pd(II) catalyst to the active Pd(0) followed by oxidative insertion in the iodoglucal C–I bond affords the palladium intermediate **I**. This intermediate undergoes CO insertion to provide the acyl palladium intermediate (**II**) (Scheme 4).^27^ Next, the amine partner interacts with the Pd center, promoting the deprotonation of the amine mediated by the DIPEA, producing the ammonium iodide salt and intermediate **III**. Finally a reductive elimination proceeds via the attack of the carbonyl by the amine nitrogen, displacing the glucoamide product (**3**) and the Pd(0) catalyst.

This mechanistic proposal is in line with the experimental observation that the alkylamines only show trace amounts of the desired products (**3v**, **3w**, **3x**, **3y**, **3z**) in comparison to the 90% yield observed for aniline (**3a**). The formation of intermediate **III** depends on the proton abstraction from the amines mediated by the Pd center that would not be favorable for alkylamines or other heterocycles and would be enhanced by electron withdrawing groups in the aromatic amines.

Nevertheless, the amine formed in intermediate **III** needs to be sufficiently nucleophilic to attack the carbonyl group to form the product, which would be favored by the electron-donating groups. Thus, a balance between these two behaviors is necessary for the reaction to take place, explaining why there is no clear trend in the yields observed by changing the aniline substituents. This hypothesis is supported by theoretical calculations carried out at the M06-2x/Def2-SVP level of theory^28^ (See Figure S1 in Supporting Information) that show that intermediate **III** is 4.5 kcal/mol more stable than the isolated reactants when aniline is used, in contrast to benzylamine, which forms intermediate **III** 4.3 kcal/mol higher in energy than the reactants. This energy difference suggests that the formation of this intermediate **III** from benzylamine is more difficult than aniline, not allowing the product formation.

In summary, we have developed a palladium-catalyzed aminocarbonylation coupling reaction utilizing 1-iodoglycals and amines under phosphine-free conditions, employing Pd-PEPPSI as a catalyst. The method exhibits a broad scope for aryl, heteroaryl, and amino esters as coupling partners. Furthermore, we have demonstrated the synthetic versatility of the substrate scope by incorporating phenols to prepare glucal esters.

## Experimental Section

### General Information

All reagents were purchased from Sigma-Aldrich, Alfa Aesar, Acros Organics, Oakwood or Fluorochem. When they were not a HPLC-grade solvents, they were purified by distillation. Other solvents, like DIPEA was also dried over CaH_2_. Thin Layer Chromatography was carried out using g Merck TLC 60 F254 silica gel plates and visualized under UV light (254 nm) and stained with acidic vanillin solution. Flash column chromatography was performed using silica gel with a pore size of 60 Å, 230–400 Mesh (Sigma-Aldrich, cat.# 22,719-6). Nuclear magnetic resonance (NMR) spectra were recorded in CDCl_3_ or DMSO-*d*_6_ using a Bruker DPX 300 or 400 instrument (^1^H at 300 or 400 MHz, ^13^C at 75 or 101 MHz). Chemical shifts, δ, are reported in parts per million (ppm) and are referenced to the tetramethylsilane (TMS) signal. ^1^H peaks are quoted to the nearest 0.01 Hz and ^13^C peaks are quoted to the nearest 0.1 Hz. The abbreviation utilized to report the peaks are s (singlet), d (doublet), t (triplet), dd (doublet of doublets) m (multiplet). High-resolution mass spectra (HRMS) were recorded on a Shimadzu ESI-TOF mass spectrometer. Fourier transform infrared (FTIR) data were obtained using an Agilent Technologies Cary 630. Optical rotations were measured at 20 °C by using an Anton Paar MCP200 Polarimeter.

### General Procedure for Synthesis of C1-d-Amidoglycals (**3**)

O-TIPS-iodoglucal or O-TIPS-iodorhamnal (0.1 mmol, 74.1 mg or 56.9 mg, 1 equiv), DIPEA (0.2 mmol, 35 μL, 2 equiv), aniline (0.12 mmol, 1.2 equiv), 1,4-dioxane (0.4 mL), were added to a flame-dried 10 mL reaction tube. Then, Mo(CO)_6_ (0.1 mmol, 26.4 mg, 1 equiv), PdPEPPSI-IPr (0.01 mmol, 10 mol %, 6.8 mg) and 1,4-dioxane (0.4 mL) the reaction tube was capped. The mixture was then stirred at 80 °C for 16 h. The mixture was filtered through a pad of Celite and thoroughly rinsed with EtOAc. The organic layer was washed with saturated aqueous solution of NH_4_Cl. The crude mixture was purified by flash column chromatography (eluent: 0 to 40% dichloromethane (DCM) in hexanes). The 1,4-dioxane was degassed by Freeze–pump–thaw prior to use.

### General Procedure for Synthesis of C1-d-Glycal Esters (**4**)

O-TIPS-iodoglucal (0.1 mmol, 74.1 mg, 1 equiv), DIPEA (0.2 mmol, 35 μL, 2 equiv), alcohol (0.12 mmol, 1.2 equiv), 1,4-dioxane (0.4 mL), were added to a flame-dried 10 mL reaction tube. Then, Mo(CO)_6_ (0.1 mmol, 26.4 mg, 1 equiv), Pd(OAc)_2_ (10 mol %, 2.2 mg), Xantphos (10 mol %, 5.8 mg) and 1,4-dioxane (0.4 mL) the reaction tube was capped. The mixture was then stirred at 80 °C for 16 h. The mixture was filtered through a pad of Celite and thoroughly rinsed with EtOAc. The organic layer was washed with saturated aqueous solution of NH_4_Cl. The crude mixture was purified by flash column chromatography (eluent: 0 to 40% DCM in hexanes). The 1,4-dioxane was degassed by Freeze–pump–thaw prior to use.

#### (2*R*,3*R*,4*R*)-*N*-Phenyl-3,4-bis((triisopropylsilyl)oxy)-2-(((triisopropylsilyl)oxy)methyl)-3,4-dihydro-2*H*-pyran-6-carboxamide (**3a**)

The product was obtained as a yellow oil (66.1 mg, 90%). [α]_D_^20^ = −26 (*c* = 0.1 in CHCl_3_). ^1^H NMR (300 MHz, CDCl_3_) δ 8.46 (bs, 1H), 7.62 (d, *J* = 7.6 Hz, 2H), 7.33 (t, *J* = 7.7 Hz, 2H), 7.11 (t, *J* = 7.5 Hz, 1H), 6.19 (d, *J* = 4.9 Hz, 1H), 4.50 (d, *J* = 8.5 Hz, 1H), 4.11–4.16 (m, 2H), 3.75 (d, *J* = 13.1 Hz, 1H), 1.07 (m, 63H). ^13^C NMR (75 MHz, CDCl_3_) δ 160.2, 143.1, 137.7, 129.1 (2C), 124.4, 119.9 (2C), 105.2, 82.5, 70.1, 65.8, 61.4, 18.3–18.0 (18C), 12.6 (3C), 12.4 (3C), 12.1 (3C). IR (ν, cm^–1^) = 3290; 2846; 2771; 1644; 1605; 1546; 1479; 1413; 1397; 1274; 1201; 1022; 853; 730. HRMS (ESI-TOF) calcd 756.4850 [C_40_H_75_NO_5_Si_3_ + Na^+^], found 756.4861.

#### (2*R*,3*R*,4*R*)-*N*-(2-Methoxyphenyl)-3,4-bis((triisopropylsilyl)oxy)-2-(((triisopropylsilyl)oxy)methyl)-3,4-dihydro-2*H*-pyran-6-carboxamide (**3b**)

The product was obtained as a yellow oil (29.0 g, 38%). [α]_D_^20^ = −26 (*c* = 0.1 in CHCl_3_). ^1^H NMR (300 MHz, CDCl_3_) (mixture of rotamers): δ 9.08 (bs, 0.7H), 8.43 (dd, *J* = 7.9, 1.7 Hz, 0.8H), 8.39 (br s, 0.2H), 7.54 (d, *J* = 5.0 Hz, 0.5H), 7.26 (t, *J* = 7.7 Hz, 0.5H), 7.06–7.00 (m, 0.4H), 6.96 (dd, *J* = 7.8 Hz, 1.8 Hz 0.7H) 6.92–6.86 (m, 0.8H), 6.82–6.79 (m, 0.8H), 6.12–6.09 (m, 1H), 4.45–4.41 (m, 1H), 4.09–3.98 (m, 3H), 3.79 (s, 3H), 3.74–3.64 (m, 1H), 1.00–0.96 (m, 63H). ^13^C NMR (75 MHz, CDCl_3_) rotameric mixture, resonances for minor rotamer are enclosed in parentheses (): δ (160.1), 160.0 148.4, 143.6 (143.0), 128.9 (127.5), (124.3) 123.8, 121.0, 119.9 (119.7), 109.9, (105.1) 104.6, 82.4, 70.0, 65.7, 61.4, 55.6, 18.1–17.9 (18C), 12.5 (3C), 12.3 (3C), 12.0 (3C). IR (ν, cm^–1^) = 3289; 2846; 2771; 1641; 1601; 1551; 1479; 1413; 1391; 1210; 1059; 1024; 853; 724. HRMS (ESI-TOF) calcd 786.4956 [C_41_H_77_NO_6_Si_3_ + Na^+^], found 786.4948.

#### (2*R*,3*R*,4*R*)-*N*-(3-Methoxyphenyl)-3,4-bis((triisopropylsilyl)oxy)-2-(((triisopropylsilyl)oxy)methyl)-3,4-dihydro-2*H*-pyran-6-carboxamide (**3c**)

The product was obtained as a beige oil (45.9 mg, 60%). [α]_D_^20^ = −30 (*c* = 0.1 in CHCl_3_). ^1^H NMR (300 MHz, CDCl_3_) δ 8.46 (bs, 1H), 7.44–7.43 (m, 1H), 7.21 (m, 1H), 7.04 (d, *J* = 7.8 Hz, 1H), 6.67 (dd, *J* = 8.2 Hz, 2.5 Hz, 1H), 6.18 (dd, *J* = 5.3 Hz, 1.7 Hz, 1H), 4.49 (dd, *J* = 8.82 Hz, 2.73 Hz, 1H), 4.17–4.05 (m, 2H), 4.06–4.05 (m, 1H), 3.81 (s, 3H), 3.74 (dd, *J* = 11.7 Hz, 2.9 Hz, 1H), 1.08–1.04 (m, 63H). ^13^C NMR (75 MHz, CDCl_3_) δ 160.1, 160.0, 142.9, 138.7, 129.5, 111.8, 110.7, 105.1, 105.0, 82.4, 69.9, 65.6, 61.2, 55.2, 18.1–17.9 (18C), 12.4 (3C), 12.3 (3C), 11.9 (3C). IR (ν, cm^–1^) = 3288; 2844; 2771; 1642; 1602; 1549; 1470; 1209; 1062; 853; 737. HRMS (ESI-TOF) calcd 786.4956 [C_41_H_77_NO_6_Si_3_ + Na^+^], found 786.4660.

#### (2*R*,3*R*,4*R*)-*N*-(4-Methoxyphenyl)-3,4-bis((triisopropylsilyl)oxy)-2-(((triisopropylsilyl)oxy)methyl)-3,4-dihydro-2*H*-pyran-6-carboxamide (**3d**)

The product was obtained as a yellow oil (34.4 mg, 45%). [α]_D_^20^ = −30 (*c* = 0.1 in CHCl_3_). ^1^H NMR (300 MHz, CDCl_3_) δ 8.37 (bs, 1H), 7.53 (dd, *J* = 6.6 Hz, 2.2 Hz, 2H), 6.86 (dd, *J* = 6.6 Hz, 2.3 Hz, 2H), 6.16 (dd, *J* = 5.2 Hz, 1.5 Hz, 1H), 4.48 (dd, *J* = 8.7 Hz, 2.6, 1H), 4.17–4.10 (m, 2H), 4.06–4.04 (m, 1H), 3.79 (s, 3H), 3.72 (dd, *J* = 11.7 Hz, 2.9 Hz, 1H), 1.09–1.04 (m, 63H). ^13^C NMR (75 MHz, CDCl_3_) δ 160.0, 156.5, 143.2, 130.9, 121.4 (2C), 114.2 (2C), 105.0, 82.5, 70.1, 65.8, 61.4, 55.6, 18.2–17.8 (18C), 12.6 (3C), 12.4 (3C), 12.1 (3C). IR (ν, cm^–1^) = 3298; 2844; 2771; 1637; 1601; 1546; 1475; 1464; 1413; 1207; 1061; 855; 726. HRMS (ESI-TOF) calcd 786.4956 [C_41_H_77_NO_6_Si_3_ + Na^+^], found 786.4975.

#### (2*R*,3*R*,4*R*)-*N*-(*p*-Tolyl)-3,4-bis((triisopropylsilyl)oxy)-2-(((triisopropylsilyl)oxy)methyl)-3,4-dihydro-2*H*-pyran-6-carboxamide (**3e**)

The product was obtained as a pale-yellow oil (26.9 mg, 36%). [α]_D_^20^ = −25 (*c* = 0.1 in CHCl_3_). ^1^H NMR (300 MHz, CDCl_3_) δ 8.41 (bs, 1H), 7.50 (d, *J* = 8.1 Hz, 2H), 7.13 (d, *J* = 8.1 Hz, 2H), 6.17 (d, *J* = 5.3 Hz, 1.6 Hz, 1H), 4.48 (dd, *J* = 8.6 Hz, 2.7 Hz, 1H), 4.17–4.10 (m, 2H), 4.06–4.05 (m, 1H), 3.73 (dd, *J* = 11.7 Hz, 3 Hz, 1H), 2.32 (s, 3H), 1.07–1.04 (m, 63H). ^13^C NMR (75 MHz, CDCl_3_) δ 160.0, 143.2, 135.1, 134.0, 129.5(2C), 119.8 (2C), 105.0, 82.5, 70.1, 65.8, 61.4, 21.0, 18.2–18.0 (18C), 12.6 (3C), 12.4 (3C), 12.1 (3C). IR (ν, cm^–1^) = 3295, 2846; 2771; 1641; 1601; 1544; 1475; 1413; 1024; 855; 726. HRMS (ESI-TOF) calcd 786.4746 [C_41_H_77_NO_5_Si_3_ + K^+^], found 786.4774.

#### (2*R*,3*R*,4*R*)-*N*-Mesityl-3,4-bis((triisopropylsilyl)oxy)-2-(((triisopropylsilyl)oxy)methyl)-3,4-dihydro-2*H*-pyran-6-carboxamide (**3f**)

The product was obtained as a pale beige oil (35.6 mg, 46%). [α]_D_^20^ = −39 (*c* = 0.1 in CHCl_3_). Mp 116–118 °C. ^1^H NMR (300 MHz, CDCl_3_) δ 7.92 (bs, 1H), 6.80 (s, 2H), 6.13 (dd, *J* = 5.4 Hz, 1.6 Hz, 1H), 4.49 (d, *J* = 8.6 Hz, 1H), 4.21–4.14 (m, 2H), 4.08–4.06 (m, 1H), 3.76 (dd, *J* = 11.1 Hz, 2.7 Hz, 2H), 2.27 (s, 3H), 2.17 (s, 6H), 1.07 (m, 63H). ^13^C NMR (75 MHz, CDCl_3_) δ 160.8, 143.5, 136.8, 135.3 (2C), 130.9, 128.9 (2C), 104.6, 82.6, 70.1, 65.8, 61.6, 21.0, 18.3–18.0 (20C), 12.6 (3C), 12.4 (3C), 12.1 (3C). IR (ν, cm^–1^) = 3296; 2844; 1644; 1601; 1480; 1411; 1022; 853; 823; 726. HRMS (ESI-TOF) calcd 798.5320 [C_43_H_81_NO_5_Si_3_ + Na^+^], found 798.5296.

#### (2*R*,3*R*,4*R*)-*N*-(2-Chlorophenyl)-3,4-bis((triisopropylsilyl)oxy)-2-(((triisopropylsilyl)oxy)methyl)-3,4-dihydro-2*H*-pyran-6-carboxamide (**3g**)

The product was obtained as a beige solid (33.5 mg, 43%). [α]_D_^20^ = −50 (*c* = 0.1 in CHCl_3_). Mp 41–43 °C. ^1^H NMR (300 MHz, CDCl_3_) δ 9.15 (bs, 1H), 8.52 (d, *J* = 8.1 Hz, 1H), 7.37 (d, *J* = 8.0 Hz, 1H), 7.30–7.24 (m, 1H), 7.06–7.00 (m, 1H), 6.19 (dd, *J* = 5.3 Hz, 1.6 Hz, 1H), 4.53 (dd, *J* = 8.3 Hz, 2.8 Hz, 1H), 4.17–4.08 (m, 3H), 3.79 (dd, *J* = 11.7 Hz, 3.0 Hz, 1H), 1.11–1.02 (m, 63H). ^13^C NMR (75 MHz, CDCl_3_) δ 160.3, 143.2, 134.6, 129.1, 127.7, 124.6, 123.3, 121.4, 105.5, 82.8, 70.1, 65.8, 61.4, 18.2–18.0 (18C), 12.6 (3C), 12.4 (3C), 12.1 (3C). IR (ν, cm^–1^) = 3272, 2846; 2771; 1646; 1607; 1542; 1475; 1415; 1393; 1024; 853; 726. HRMS (ESI-TOF) calcd 790.4461 [C_40_H_74_ClNO_5_Si_3_ + Na^+^], found 790.4429.

#### (2*R*,3*R*,4*R*)-*N*-(3-Chlorophenyl)-3,4-bis((triisopropylsilyl)oxy)-2-(((triisopropylsilyl)oxy)methyl)-3,4-dihydro-2*H*-pyran-6-carboxamide (**3h**)

The product was obtained as a beige oil (41.5 mg, 54%). [α]_D_^20^ = −21 (*c* = 0.1 in CHCl_3_). ^1^H NMR (300 MHz, CDCl_3_) δ 8.51 (bs, 1H), 7.78–7.77 (m, 1H), 7.46 (dd, *J* = 8.0 Hz, 2.0 Hz, 1H), 7.28–7.23 (m, 1H), 7.10 (dd, *J* = 7.9 Hz, 2.0 Hz, 1H), 6.20 (dd, *J* = 5.3 Hz, 1.6 Hz, 1H), 4.50 (dd, *J* = 8.5 Hz, 2.6 Hz, 1H), 4.18–4.11 (m, 2H), 4.08–4.06 (m, 1H), 3.75 (dd, *J* = 11.7 Hz, 3.0 Hz, 1H), 1.09–1.06 (m, 63H). ^13^C NMR (75 MHz, CDCl_3_) δ 160.3, 142.8, 138.8, 134.8, 130.0, 124.4, 120.0, 117.8, 105.7, 82.6, 70.0, 65.7, 61.3, 18.2–18.0 (18C), 12.6 (3C), 12.4 (3C), 12.1 (3C). IR (ν, cm^–1^) = 3291; 2846; 2771; 1646; 1607; 1538; 1473; 1413; 1022; 853; 726. HRMS (ESI-TOF) calcd 790.4461 [C_40_H_74_ClNO_5_Si_3_ + Na^+^], found 790.4448.

#### (2*R*,3*R*,4*R*)-*N*-(4-Chlorophenyl)-3,4-bis((triisopropylsilyl)oxy)-2-(((triisopropylsilyl)oxy)methyl)-3,4-dihydro-2*H*-pyran-6-carboxamide (**3i**)

The product was obtained as a yellow oil (50.3 mg, 65%). [α]_D_^20^ = −15 (*c* = 0.1 in CHCl_3_). ^1^H NMR (300 MHz, CDCl_3_) δ 8.46 (bs, 1H), 7.57 (d, *J* = 8.3 Hz, 2H), 7.29 (d, *J* = 8.4 Hz, 2H), 6.17 (d, *J* = 5.1 Hz, 1H), 4.48 (d, *J* = 8.7 Hz, 1H), 4.16–4.12 (m, 2H), 4.04 (m, 1H), 3.72 (dd, *J* = 11.8 Hz, 3.0 Hz, 1H), 1.06 (m, 63H). ^13^C NMR (75 MHz, CDCl_3_) δ 160.2, 142.9, 136.2, 129.4, 129.1 (2C), 121.0 (2C), 105.6, 82.6, 70.1, 65.7, 61.3, 18.2–18.0 (18C), 12.6 (3C), 12.4 (3C), 12.1 (3C). IR (ν, cm^–1^) = 3291; 2846; 2771; 1646; 1605; 1540; 1471; 1413; 1022; 853; 726. HRMS (ESI-TOF) calcd 790.4461 [C_40_H_74_ClNO_5_Si_3_ + Na^+^], found 790.4448.

#### (2*R*,3*R*,4*R*)-*N*-(4-Bromophenyl)-3,4-bis((triisopropylsilyl)oxy)-2-(((triisopropylsilyl)oxy)methyl)-3,4-dihydro-2*H*-pyran-6-carboxamide (**3j**)

The product was obtained as a yellow oil (73.1 mg, 90%). [α]_D_^20^ = −46 (*c* = 0.1 in CHCl_3_). ^1^H NMR (300 MHz, CDCl_3_) δ 8.46 (bs, 1H), 7.52 (dd, *J* = 8.7 Hz, 2.2 Hz, 2H), 7.43 (dd, *J* = 8.7 Hz, 2.3 Hz, 2H), 6.18 (dd, *J* = 5.3 Hz, 1.6 Hz, 1H), 4.48 (dd, *J* = 8.8 Hz, 2.4 Hz, 1H), 4.16–4.10 (m, 2H), 4.06–4.04 (m, 1H), 4.73 (dd, *J* = 11.7 Hz, 2.9 Hz, 1H), 1.07–1.05 (m, 63H). ^13^C NMR (75 MHz, CDCl_3_) δ 166.2, 142.9, 136.7, 132.0 (2C), 121.4 (2C), 117.0, 105.6, 82.6, 70.1, 65.7, 61.3, 18.2–18.0 (18C), 12.6 (3C), 12.4 (3C), 12.1 (3C). IR (ν, cm^–1^) = 3291; 2846; 2771; 1639; 1605; 1538; 1471; 1413; 1022; 853; 726. HRMS (ESI-TOF) calcd 834.3955 [C_40_H_74_BrNO_5_Si_3_ + Na^+^], found 834.3953.

#### (2*R*,3*R*,4*R*)-*N*-(4-Fluorophenyl)-3,4-bis((triisopropylsilyl)oxy)-2-(((triisopropylsilyl)oxy)methyl)-3,4-dihydro-2*H*-pyran-6-carboxamide (**3k**)

The product was obtained as a brown oil (41.4 mg, 55%). [α]_D_^20^ = −26 (*c* = 0.1 in CHCl_3_). ^1^H NMR (300 MHz, CDCl_3_) δ 8.44 (bs, 1H), 7.60–7.55 (m, 2H), 7.05–6.99 (m, 2H), 6.18 (dd, *J* = 5.3 Hz, 1.6 Hz, 1H), 4.48 (dd, *J* = 8.6 Hz, 2.5 Hz, 1H), 4.17–4.10 (m, 2H), 4.06–4.05 (m, 1H), 3.73 (dd, *J* = 11.7 Hz, 2.9 Hz, 1H), 1.07–1.04 (m, 63H). ^13^C NMR (75 MHz, CDCl_3_) δ 160.2, 159.5 (d, *J* = 242 Hz, C–F), 143.0, 133.7 (d, *J* = 3 Hz, C–F), 121.5 (d, *J* = 8.2 Hz, C–F), 115.7 (d, *J* = 22.5 Hz, C–F), 105.4, 82.5, 70.1, 65.7, 61.3, 18.3–18.0 (18C), 12.6 (3C), 12.4 (3C), 12.1 (3C). ^19^F NMR (282 MHz, CDCl_3_) δ −117.08. IR (ν, cm^–1^) = 3295; 2846; 2771; 1642; 1605; 1557; 1477; 1460; 1413; 1024; 855; 726. HRMS (ESI-TOF) calcd 774.4756 [C_40_H_74_FNO_5_Si_3_ + Na^+^], found 774.4780.

#### (2*R*,3*R*,4*R*)-*N*-(4-(Trifluoromethyl)phenyl)-3,4-bis((triisopropylsilyl)oxy)-2-(((triisopropylsilyl)oxy)methyl)-3,4-dihydro-2*H*-pyran-6-carboxamide (**3l**)

The product was obtained as a brown oil (63.3 mg, 79%). [α]_D_^20^ = −38 (*c* = 0.1 in CHCl_3_). ^1^H NMR (300 MHz, CDCl_3_) δ 8.60 (bs, 1H), 7.74 (d, *J* = 8.4 Hz, 1H), 7.59 (d, *J* = 8.4 Hz, 2H), 6.20 (dd, *J* = 5.3 Hz, 1.6 Hz, 1H), 4.51 (d, *J* = 7.8 Hz, 1H), 4.18–4.11 (m, 2H), 4.07–4.05 (m, 1H), 3.73 (dd, *J* = 11.7 Hz, 2.9 Hz, 1H), 1.09–1.05 (m, 63H). ^13^C NMR (75 MHz, CDCl_3_) δ 160.4, 142.7, 140.7, 126.3 (q, *J* = 4.0 Hz, C–F, 2C), 126.0 (q, *J* = 21 Hz, C–F), 121.0 (q, *J* = 227 Hz, C–F), 119.5 (2C), 106.0, 82.7, 70.0, 65.6, 61.2, 18.2–18.0 (18C), 12.6 (3C), 12.4 (3C), 12.1 (3C). ^19^F NMR (282 MHz, CDCl_3_) δ −61.25. IR (ν, cm^–1^) = 3289; 2846; 2771; 1646; 1605; 1547; 1479; 1415; 1279; 1024; 855; 726. HRMS (ESI-TOF) calcd 824.4724 [C_41_H_74_F_3_NO_5_Si_3_ + K^+^], found 824.4721.

#### (2*R*,3*R*,4*R*)-*N*-(4-Nitrophenyl)-3,4-bis((triisopropylsilyl)oxy)-2-(((triisopropylsilyl)oxy)methyl)-3,4-dihydro-2*H*-pyran-6-carboxamide (**3m**)

The product was obtained as a beige solid (47.7 mg, 61%). [α]_D_^20^ = −25 (*c* = 0.1 in CHCl_3_). Mp 113–115 °C. ^1^H NMR (300 MHz, CDCl_3_) δ 8.76 (bs, 1H), 8.23 (d, *J* = 9.2 Hz, 2H), 7.79 (d, *J* = 9.0 Hz, 2H), 6.23 (d, *J* = 5.1 Hz, 1H), 4.52 (d, *J* = 8.3 Hz, 1H), 4.18–4.11 (m, 2H), 4.06 (m, 1H), 3.73 (dd, *J* = 11.8 Hz, 2.8 Hz, 1H), 1.07–1.05 (m, 63H). ^13^C NMR (75 MHz, CDCl_3_) δ 160.5, 143.8, 143.3, 142.4, 125.2 (2C), 119.3 (2C), 106.7, 82.8, 70.0, 65.6, 61.2, 18.2–18.0 (18C), 12.6 (3C), 12.4 (3C), 12.1 (3C). IR (ν, cm^–1^) = 3263; 2846; 2771; 1648; 1601; 1557; 1456; 1413; 1290; 1022; 853; 823; 720. HRMS (ESI-TOF) calcd 801.4701 [C_40_H_74_N_2_O_7_Si_3_ + Na^+^], found 801.4727.

#### Ethyl 4-((2*R*,3*R*,4*R*)-3,4-Bis((triisopropylsilyl)oxy)-2-(((triisopropylsilyl)oxy)methyl)-3,4-dihydro-2*H*-pyran-6-carboxamido)benzoate (**3n**)

The product was obtained as a yellow oil (27.6 mg, 34%). [α]_D_^20^ = −31 (*c* = 0.1 in CHCl_3_). ^1^H NMR (300 MHz, CDCl_3_) δ 8.62 (bs, 1H), 8.03 (d, *J* = 8.6 Hz, 2H), 7.69 (d, *J* = 8.6 Hz, 2H), 6.20 (d, *J* = 4.2 Hz, 1H), 4.50 (d, *J* = 7.2 Hz, 1H), 4.36 (q, *J* = 7.0 Hz, 2H), 4.17–4.10 (m, 2H), 4.05 (m, 1H), 3.73 (dd, *J* = 11.7 Hz, 2.7 Hz, 1H), 1.39 (t, *J* = 7.1 Hz, 3H), 1.07–1.05 (m, 63H). ^13^C NMR (75 MHz, CDCl_3_) δ 166.2, 160.4, 142.8, 141.7, 130.9 (2C), 126.2, 119.0 (2C), 105.9, 82.6, 70.0, 65.7, 61.3, 60.9, 18.2–18.0 (20C), 14.5, 12.6 (3C), 12.4 (3C), 12.1 (3C). IR (ν, cm^–1^) = 3289; 2846; 2771; 1663; 1646; 1475; 1415; 1233; 1061; 1024; 855; 745. HRMS (ESI-TOF) calcd 828.5062 [C_43_H_79_NO_7_Si_3_ + Na^+^], found 828.5045.

#### (2*R*,3*R*,4*R*)-*N*-([1,1′-Biphenyl]-2-yl)-3,4-bis((triisopropylsilyl)oxy)-2-(((triisopropylsilyl)oxy)methyl)-3,4-dihydro-2*H*-pyran-6-carboxamide (**3o**)

The product was obtained as a beige solid (36.5 mg, 45%). Mp 81–83 °C. [α]_D_^20^ = −19 (*c* = 0.1 in CHCl_3_). ^1^H NMR (300 MHz, CDCl_3_) δ 8.75 (bs, 1H), 8.53 (d, *J* = 8.2 Hz, 1H), 7.45–7.35 (m, 6H), 7.28–7.25 (m, 1H), 7.19–7.14 (m, 1H), 6.13 (dd, *J* = 5.3 Hz, 1.5 Hz, 1H), 4.22–4.18 (m, 1H), 4.13–4.07 (m, 2H), 3.82–3.77 (m, 2H), 1.06–0.97 (m, 63H). ^13^C NMR (75 MHz, CDCl_3_) δ 160.0, 143.5, 138.0, 134.7, 132.4, 130.1, 129.3 (2C), 128.9 (2C), 128.5, 127.9, 124.2, 120.8, 104.9, 82.0, 69.7, 65.9, 61.1, 18.2–18.0 (18C), 12.6 (3C), 12.5 (3C), 12.0 (3C). IR (ν, cm^–1^) = 3274; 2844; 2769; 1639; 1607; 1534; 1477; 1404; 1058; 1024; 853; 735. HRMS (ESI-TOF) calcd 832.5163 [C_46_H_79_NO_5_Si_3_ + Na^+^], found 832.5151.

#### (2*R*,3*R*,4*R*)-*N*-(Naphthalen-1-yl)-3,4-bis((triisopropylsilyl)oxy)-2-(((triisopropylsilyl)oxy)methyl)-3,4-dihydro-2*H*-pyran-6-carboxamide (**3p**)

The product was obtained as a beige oil (5.6 mg, 7%). [α]_D_^20^ = −27 (*c* = 0.1 in CHCl_3_). ^1^H NMR (300 MHz, CDCl_3_) δ 9.06 (bs, 1H), 8.22 (d, *J* = 7.5 Hz, 1H), 7.88–7.85 (m, 2H), 7.68 (d, *J* = 8.3 Hz, 1H), 7.51–7.49 (m, 3H), 6.25 (d, *J* = 5.2 Hz, 1H), 4.59 (d, *J* = 8.7 Hz, 1H), 4.25–4.28 (m, 2H), 4.11 (m, 1H), 3.80 (dd, *J* = 12.0 Hz, 2.2 Hz, 1H), 1.09–1.05 (m, 63H). ^13^C NMR (75 MHz, CDCl_3_) δ 160.6, 143.4, 134.2, 132.2, 128.9, 126.6, 126.05, 126.03, 125.4, 120.4, 119.4, 105.4, 82.9, 70.2, 65.8, 61.6, 18.3–18.2 (18C), 12.6 (3C), 12.5 (3C), 12.1 (3C). IR (ν, cm^–1^) = 3306; 2844; 2771; 1646; 1605; 1486; 1451; 1413; 1026; 855; 747. HRMS (ESI-TOF) calcd 822.4746 [C_44_H_77_NO_5_Si_3_ + K^+^], found 822.4761.

#### (2*R*,3*R*,4*R*)-*N*-(Benzo[*d*]thiazol-5-yl)-3,4-bis((triisopropylsilyl)oxy)-2-(((triisopropylsilyl)oxy)methyl)-3,4-dihydro-2*H*-pyran-6-carboxamide (**3s**)

The product was obtained as a pale-yellow oil (22.1 mg, 28%). [α]_D_^20^ = −27 (*c* = 0.1 in CHCl_3_). ^1^H NMR (300 MHz, CDCl_3_) δ 9.05 (bs, 1H), 8.77 (s, 1H), 8.69 (s, 1H), 8.13 (d, *J* = 8.6 Hz, 1H), 7.41 (d, *J* = 8.6 Hz, 1H), 6.22 (d, *J* = 5.3 Hz, 1H), 4.53–4.50 (m, 1H), 4.18–4.07 (m, 3H), 3.75 (d, *J* = 11.8 Hz, 2H), 1.07–1.05 (m, 63H). ^13^C NMR (75 MHz, CDCl_3_) δ 160.4, 142.8, 135.9, 123.2, 119.6, 112.5, 105.9, 82.6, 70.0, 65.7, 61.3, 18.2–18.0 (18C), 12.6 (3C), 12.4 (3C), 12.1 (3C). IR (ν, cm^–1^) = 3293; 2844; 2769; 1631; 1601; 1520; 1473; 1413; 1356; 1203; 1059; 1024; 853; 726. HRMS (ESI-TOF) calcd 813.4523 [C_41_H_74_N_2_O_5_SSi_3_ + Na^+^], found 813.4532.

#### (*R*)-Methyl 2-((2*R*,3*R*,4*R*)-3,4-Bis((triisopropylsilyl)oxy)-2-(((triisopropylsilyl)oxy)methyl)-3,4-dihydro-2*H*-pyran-6-carboxamido)-3-phenylpropanoate (**3t**)

The product was obtained as a pale-yellow oil (16.4 mg, 20%). [α]_D_^20^ = −26 (*c* = 0.1 in CHCl_3_). ^1^H NMR (300 MHz, CDCl_3_) δ 7.25–7.20 (m, 3H), 7.11 (d, *J* = 7.2 Hz, 2H), 6.05 (d, *J* = 5.2 Hz, 1H), 4.95–4.88 (m, 1H), 4.40–4.36 (m, 1H), 4.12–4.07 (m, 2H), 3.98 (dd, *J* = 11.4 Hz, 8.6 Hz, 1H), 3.79–3.74 (m. 1H), 3.67 (s, 3H), 3.21–3.06 (m, 2H), 1.06–1.02 (m, 63H). ^13^C NMR (75 MHz, CDCl_3_) δ 171.4, 161.9, 143.1, 136.0, 129.4 (2C), 128.2 (2C), 127.1, 104.6, 82.1, 69.9, 65.8, 61.3, 53.4, 52.2, 38.3, 18.2–17.8 (18C), 12.6 (3C), 12.4 (3C), 12.1 (3C). IR (ν, cm^–1^) = 3304; 2844; 2771; 1693; 1631; 1601; 1460; 1413; 1059; 1028; 855; 730. HRMS (ESI-TOF) calcd 858.4957[C_44_H_81_NO_7_Si_3_ + K^+^], found 858.4990.

#### (*R*)-Methyl 2-((2*R*,3*R*,4*R*)-3,4-Bis((triisopropylsilyl)oxy)-2-(((triisopropylsilyl)oxy)methyl)-3,4-dihydro-2*H*-pyran-6-carboxamido)-4-(methylthio)butanoate (**3u**)

The product was obtained as a pale-yellow oil (25.7 mg, 32%). [α]_D_^20^ = −36 (*c* = 0.1 in CHCl_3_). ^1^H NMR (300 MHz, CDCl_3_) δ 7.20 (d, *J* = 8.0 Hz 1H), 6.05 (dd, *J* = 5.3 Hz, 1.6 Hz, 1H), 4.79–4.73 (m, 1H), 4.44–4.40 (m, 1H), 4.12–4.01 (m, 3H), 3.77 (dd, *J* = 11,5 Hz, 3.6 Hz, 1H), 3.74 (s, 3H), 2.51–2.46 (m, 2H), 2.27–2.15 (m, 1H), 2.07 (s, 3H), 2.04–1.94 (m, 1H), 1.05 (m, 63H). ^13^C NMR (75 MHz, CDCl_3_) δ 171.8, 162.2, 143.0, 104.8, 82.3, 69.9, 65.8, 61.4, 52.5, 51.5, 32.1, 30.0, 18.2–18.1 (18C), 15.6, 12.6 (3C), 12.4 (3C), 12.1 (3C). IR (ν, cm^–1^) = 3308; 2846; 2771; 1689; 1631; 1601; 1460; 1413; 1052; 1026; 855; 730; 659. HRMS (ESI-TOF) calcd 826.4939 [C_40_H_81_NO_7_SSi_3_ + K^+^], found 826.4951.

#### (2*S*,3*S*,4*S*)-*N*-(4-Chlorophenyl)-2-methyl-3,4-bis((triisopropylsilyl)oxy)-3,4-dihydro-2*H*-pyran-6-carboxamide (**3aa**)

The product was obtained as a pale-yellow oil (41.0 mg, 68%). [α]_D_^20^ = +34.4 (*c* = 0.9 in CHCl_3_). ^1^H NMR (400 MHz, CDCl_3_) δ 8.36 (bs, 1H), 7.61–7.59 (m, 2H), 7.31–7.29 (m, 2H), 6.20–6.18 (m, 1H), 4.55–4.50 (m, 1H), 4.20–4.19 (m, 1H), 3.98–3.97 (m, 1H), 1.41 (d, *J* = 7.1 Hz, 3H), 1.07–1.04 (m, 42H). ^13^C NMR (101 MHz, CDCl_3_) δ 160.5, 142.5, 136.2, 129.4, 129.1 (2C), 121.1 (2C), 105.7, 76.5, 72.9, 66.4, 18.3 (3C), 18.2 (3C), 18.2 (3C), 18.2 (3C), 15.8, 12.6 (3C), 12.5 (3C). IR (ν, cm^–1^) = 3310; 2945, 2868; 1683; 1655; 1593; 1524, 1494; 1464; 1402, 1095; 1062; 883; 682. HRMS (ESI-TOF) calcd 618.3178 [C31H_54_ClNO_4_Si_2_ + Na^+^], found 618.3203.

#### (2*R*,3*R*,4*R*)-Phenyl 3,4-Bis((triisopropylsilyl)oxy)-02-(((triisopropylsilyl)oxy)methyl)-3,4-dihydro-2*H*-pyran-06-carboxylate (**4a**)

The product was obtained as a yellow oil (40.4 mg, 55%). [α]_D_^20^ = −46 (*c* = 0.1 in CHCl_3_). ^1^H NMR (300 MHz, CDCl_3_) δ 7.38 (t, *J* = 7.5 Hz, 2H), 7.22 (d, *J* = 7.3 Hz, 1H), 7.14 (d, *J* = 8.0 Hz, 2H), 6.25 (d, *J* = 5.3 Hz, 1H), 4.53 (m, 1H), 4.23–4.21 (m, 2H), 4.06 (dd, *J* = 11.1 Hz, 7.4 Hz, 1H), 3.94 (dd, *J* = 11.1 Hz, 5.3 Hz, 1H), 1.10–1.06 (m, 63H). ^13^C NMR (75 MHz, CDCl_3_) δ 161.6, 150.8, 142.0, 129.4 (2C), 125.9, 121.7 (2C), 109.7, 81.8, 69.5, 66.0, 61.4, 18.2–18.0 (18C), 12.6 (3C), 12.5 (3C), 12.1 (3C). IR (ν, cm^–1^) = 2846; 2771; 1691; 1596; 1415; 1210; 1158; 1065; 1018; 855; 722. HRMS (ESI-TOF) calcd 757.4690 [C_40_H_74_O_6_Si_3_ + Na^+^], found 757.4696.

#### (2*R*,3*R*,4*R*)-Naphthalen-2-yl 3,4-Bis((triisopropylsilyl)oxy)-2-(((triisopropylsilyl)oxy)methyl)-3,4-dihydro-2*H*-pyran-6-carboxylate (**4b**)

The product was obtained as a beige oil (25.4 mg, 33%). [α]_D_^20^ = −20 (*c* = 0.1 in CHCl_3_). ^1^H NMR (300 MHz, CDCl_3_) δ 7.88–7.80 (m, 3H), 7.62 (m, 1H), 7.52–7.46 (m, 2H), 7.29 (m, 1H), 6.29 (d, *J* = 5.4 Hz, 1H), 4.57–4.53 (m, 1H), 4.24–4.21 (m, 2H), 4.08 (m, 1H), 3.96 (dd, *J* = 11. Hz, 5.5 Hz, 1H), 1.13–1.06 (m, 63H). ^13^C NMR (75 MHz, CDCl_3_) δ 161.8, 148.5, 142.1, 133.9, 131.6, 129.4, 127.9, 127.8, 126.6, 125.8, 121.2, 118.7, 109.9, 81.8, 69.5, 66.0, 61.4, 18.3–18.1 (18C), 12.7 (3C), 12.5 (3C), 12.2 (3C). IR (ν, cm^–1^) = 2846; 2771; 1689; 1415; 1274; 1171; 1063; 1017; 855; 724. HRMS (ESI-TOF) calcd 823.4586 [C_44_H_76_O_6_Si_3_ + K^+^], found 823.4592.

#### (2*R*,3*R*,4*R*)-4-Methoxyphenyl 3,4-Bis((triisopropylsilyl)oxy)-2-(((triisopropylsilyl) oxy)methyl)-3,4-dihydro-2*H*-pyran-6-carboxylate (**4c**)

The product was obtained as a beige oil (26.8 mg, 35%). [α]_D_^20^ = −20 (*c* = 0.1 in CHCl_3_). ^1^H NMR (300 MHz, CDCl_3_) δ 7.04 (d, *J* = 9.0 Hz, 2H), 6.89 (d, *J* = 9.0 Hz, 2H), 6.22 (dd, *J* = 5.1 Hz, 1.2 Hz, 1H), 4.52 (dd, *J* = 7.4 Hz, 5.2 Hz, 1H), 4.22–4.17 (m, 2H), 4.05 (dd, *J* = 11.2 Hz, 7.6 Hz, 1H), 3.33 (dd, *J* = 11.1 Hz, 5.1 Hz, 1H), 3.8 (s, 3H), 1.09–1.06 (m, 63H). ^13^C NMR (75 MHz, CDCl_3_) δ 162.0, 157.4, 144.3, 142.1, 122.4 (2C), 114.5 (2C), 109.6, 81.8, 69.5, 66.0, 61.4, 55.7, 18.3–18.0 (18C), 12.6 (3C), 12.5 (3C), 12.2 (3C). IR (ν, cm^–1^) = 2846; 2771; 1689; 1596; 1458; 1415; 1274; 1156; 1065; 1020; 855; 726. HRMS (ESI-TOF) calcd 787.4797 [C_41_H_76_O_7_Si_3_ + K^+^], found 787.4760.

#### (2*R*,3*R*,4*R*)-2-Isopropyl-5-methylphenyl 3,4-Bis((triisopropylsilyl)oxy)-2-(((triisopropylsilyl)oxy)methyl)-3,4-dihydro-2*H*-pyran-6-carboxylate (**4d**)

The product was obtained as a red-brown oil (10.3 mg, 13%). [α]_D_^20^ = −39 (*c* = 0.1 in CHCl_3_). ^1^H NMR (300 MHz, CDCl_3_) δ 7.26 (d, *J* = 7.7 Hz, 1H), 7.02 (d, *J* = 7.8 Hz, 1H), 6.87 (s, 1H), 6.26 (d, *J* = 4.9 Hz, 1H), 4.52 (m, 1H), 4.23–4.19 (m, 2H), 4.05 (dd, *J* = 10.9 Hz, 7.5 Hz, 1H), 3.95 (dd, *J* = 10.9 Hz, 4.9 Hz, 1H), 2.98 (sept, *J* = 6.9 Hz, 1H), 2.31 (s, 3H), 1.17 (d, *J* = 6.8 Hz, 6H), 1.09–1.06 (m, 63H). ^13^C NMR (75 MHz, CDCl_3_) δ 161.7, 148.0, 142.2, 137.2, 136.6, 127.2, 126.5, 122.8, 109.4, 81.8, 69.4, 66.0, 61.5, 27.4, 23.2, 23.0, 20.9, 18.2–18.1 (18C), 12.6 (3C), 12.5 (3C), 12.2 (3C). IR (ν, cm^–1^) = 2846; 2771; 1687; 1594; 1413; 1274; 1188; 1050; 1020; 855; 726. HRMS (ESI-TOF) calcd 813.5317 [C_44_H_82_O_6_Si_3_ + Na^+^], found 813.5305.

#### (2*R*,3*R*,4*R*)-4-Chlorophenyl 3,4-Bis((triisopropylsilyl)oxy)-2-(((triisopropylsilyl)oxy)methyl)-3,4-dihydro-2*H*-pyran-6-carboxylate (**4e**)

The product was obtained as a beige oil (50.0 mg, 65%). [α]_D_^20^ = −25 (*c* = 0.1 in CHCl_3_). ^1^H NMR (300 MHz, CDCl_3_) δ 7.35 (d, *J* = 8.3 Hz, 2H), 7.09 (d, *J* = 8.4 Hz, 2H), 6.23 (d, *J* = 5.3 Hz, 1H), 4.52 (dd, *J* = 6.3 Hz, 5.3 Hz, 1H), 4.21–4.18 (m, 2H), 4.06 (m, 2H), 4.05 (dd, *J* = 11.1 Hz, 7.4 Hz, 1H), 3.92 (dd, *J* = 11.1 Hz, 5.2 Hz, 1H), 1.11–1.05 (m, 63H). ^13^C NMR (75 MHz, CDCl_3_) δ 161.4, 149.3, 141.8, 131.4, 129.5 (2C), 123.1 (2C), 110.1, 81.9, 69.5, 65.9, 61.4, 18.2–18.1 (18C), 12.6 (3C), 12.5 (3C), 12.1 (3C). IR (ν, cm^–1^) = 2846; 2771; 1693; 1596; 1439; 1415; 1162; 1054; 1017; 853; 724. HRMS (ESI-TOF) calcd 791.4301 [C_40_H_73_ClO_6_Si_3_ + Na^+^], found 791.4337.

#### (2*R*,3*R*,4*R*)-4-Chlorophenyl 3,4-Bis((triisopropylsilyl)oxy)-2-(((triisopropylsilyl)oxy)methyl)-3,4-dihydro-2*H*-pyran-6-carboxylate (**4f**)

The product was obtained as a beige oil (30.1 mg, 37%). [α]_D_^20^ = −18 (*c* = 0.1 in CHCl_3_). ^1^H NMR (300 MHz, CDCl_3_) δ 7.50 (d, *J* = 8.8 Hz, 2H), 7.03 (d, *J* = 8.7 Hz, 2H), 6.23 (d, *J* = 4.3 Hz, 1H), 4.52 (dd, *J* = 7.2 Hz, 5.0 Hz, 1H), 4.21–4.17 (m, 2H), 4.05 (dd, *J* = 11.1 Hz, 7.4 Hz, 1H), 3.92 (dd, *J* = 11.1 Hz, 5.1 Hz, 1H), 1.09–1.06 (m, 63H). ^13^C NMR (75 MHz, CDCl_3_) δ 161.3, 149.8, 141.8, 132.5 (2C), 123.5 (2C), 119.1, 110.2, 81.9, 69.5, 65.9, 61.3, 18.2–18.1 (18C), 12.6 (3C), 12.5 (3C), 12.1 (3C). IR (ν, cm^–1^) = 2846; 2771; 1693; 1596; 1436; 1415; 1274; 1162; 1063; 1017; 979; 853; 724. HRMS (ESI-TOF) calcd 851.3535 [C_40_H_73_BrO_6_Si_3_ + K^+^], found 851.3583.

#### (2*R*,3*R*,4*R*)-2-Chloro-5-methylphenyl 3,4-Bis((triisopropylsilyl)oxy)-2-(((triisopropylsilyl)oxy)methyl)-3,4-dihydro-2*H*-pyran-6-carboxylate (**4g**)

The product was obtained as a beige oil (16.5 mg, 21%). [α]_D_^20^ = −23 (*c* = 0.1 in CHCl_3_). ^1^H NMR (300 MHz, CDCl_3_) δ 7.30 (d, *J* = 7.8 Hz, 1H), 7.09–6.98 (m, 2H), 6.31 (d, *J* = 4.8 Hz, 1H), 4.54–4.51 (m, 1H), 4.24–4.20 (m, 2H), 4.05 (dd, *J* = 11.1 Hz, 7.1 Hz, 1H), 3.96 (dd, *J* = 11.1 Hz, 5.2 Hz, 1H), 2.33 (s, 3H), 1.08–1.06 (m, 63H). ^13^C NMR (75 MHz, CDCl_3_) δ 160.6, 146.7, 141.6, 138.1, 129.9, 127.9, 124.3, 123.8, 110.3, 81.9, 69.4, 66.0, 61.4, 21.0, 18.2–18.1 (18C), 12.6 (3C), 12.5 (3C), 12.1 (3C). IR (ν, cm^–1^) = 2846; 2771; 1708; 1594; 1413; 1208; 1184; 1065; 1026; 855; 724. HRMS (ESI-TOF) calcd 805.4457 [C_41_H_75_ClO_6_Si_3_ + Na^+^], found 805.4488.

### Procedure for Deprotection of C1-Amidoglycals^25^

A solution of TBAF (1 M in THF, 0.324 mmol, 3.6 equiv) was added to a solution of amidoglycal **3a** or **3j** (0.09 mmol, 1.0 equiv) in anhydrous THF (500 μL) at room temperature, under N_2_ atmosphere. The mixture was stirred at room temperature for 2 h, and then quenched with water (200 μL). To the crude mixture was added silica gel for column chromatography and the solvent removed under reduced pressure. The product was purified by flash column chromatography using MeOH/EtOAc as eluent (0–10%).

#### (2*S*,3*R*,4*S*)-3,4-Dihydroxy-2-(hydroxymethyl)-*N*-phenyl-3,4-dihydro-2*H*-pyran-6-carboxamide (**5a**)

The product was obtained as a white solid (17 mg, 71%). [α]_D_^20^ = −62 (*c* = 0.1 in MeOH). Mp 88–90 °C. ^1^H NMR (300 MHz, DMSO-*d*_6_) δ 9.53 (bs, 1H), 7.65 (d, *J* = 7.6 Hz, 2H), 7.35 (t, *J* = 7.6 Hz, 2H), 7.12 (t, *J* = 7.4 Hz, 1H), 5.74 (d, *J* = 2.5 Hz, 1H), 5.32 (d, *J* = 5.6 Hz, 1H), 5.17 (d, *J* = 5.6 Hz, 1H), 4.90 (dd, *J* = 8.0 Hz, 4.8 Hz, 1H), 4.17–4.07 (m, 1H), 3.87–3.80 (m, 2H), 3.72–3.67 (m, 1H), 3.43–3.36 (m, 1H). ^13^C NMR (75 MHz, DMSO-*d*_6_) δ 159.4, 144.6, 137.7, 128.5 (2C), 124.1, 120.8 (2C), 109.4, 80.8, 68.6, 68.4, 60.6. IR (ν, cm^–1^) = 3175; 2870; 2823; 1631; 1596; 1547; 1486; 1397; 1197; 1041; 992; 946. HRMS (ESI-TOF) calcd 288.0848 [C_13_H_15_NO_5_ + Na^+^], found 288.0840.

#### (2*S*,3*R*,4*S*)-*N*-(4-Bromophenyl)-3,4-dihydroxy-2-(hydroxymethyl)-3,4-dihydro-2*H*-pyran-6-carboxamide (**5b**)

The product was obtained as a pale-yellow solid (25 mg, 80%). [α]_D_^20^ = −59 (*c* = 0.1 in MeOH). Mp 138–140 °C. ^1^H NMR (300 MHz, DMSO-*d*_6_) δ 9.61 (bs, 1H), 7.61 (d, *J* = 7.6 Hz, 2H), 7.50 (d, *J* = 7.5 Hz, 2H), 5.71 (m, 1H), 5.32 (m, 1H), 5.18 (m, 1H), 4.85 (m, 1H), 4.06 (d, *J* = 5.9 Hz, 1H), 3.81–3.77 (m, 2H), 3.68–3.62 (m, 1H), 3.39–3.33 (m, 1H). ^13^C NMR (75 MHz, DMSO-*d*_6_) δ 159.5, 144.4, 137.2, 131.4 (2C), 122.7 (2C), 115.9, 109.6, 80.8, 68.5, 68.3, 60.5. IR (ν, cm^–1^) = 3119; 2823; 2769; 1588; 1540; 1480; 1441; 1352; 1039; 1005; 946; 795. HRMS (ESI-TOF) calcd 365.9953 [C_15_H_14_BrNO_5_ + Na^+^], found 365.9948.

### Procedure for Protection of C1-Amidoglycals with Acetyl Group

To a solution of the deprotected amidoglycal (**5a**, 0.18 mmol, 48 mg) in pyridine (500 μL), under N_2_ atmosphere, Ac_2_O (250 μL) was added dropwise. The reaction mixture was stirred for 16 h, diluted with CH_2_Cl_2_ (5 mL) and washed with H_2_O (5 mL). The aqueous layer was extracted (2 × 5 mL) with CH_2_Cl_2_, and the combined organic layers dried under MgSO_4_. The crude product was purified by flash column chromatography using EtOAc/Hexanes (20%) as eluent.

#### (2*R*,3*S*,4*R*)-2-(Acetoxymethyl)-6-(phenylcarbamoyl)-3,4-dihydro-2*H*-pyran-3,4-diyl Diacetate (**6a**)

The product was obtained as a white solid (53 mg, 75%). [α]_D_^20^ = −62 (*c* = 0.1 in CHCl_3_). Mp 100–102 °C. ^1^H NMR (300 MHz, CDCl_3_) δ 8.23 (bs, 1H), 7.60 (d, *J* = 7.5 Hz, 2H), 7.35 (t, *J* = 7.5 Hz, 2H), 7.14 (t, *J* = 7.4 Hz, 2H), 6.12 (d, *J* = 3.6 Hz, 1H), 5.53 (dd, *J* = 5.3 Hz, 3.6 Hz, 1H), 5.26 (t, *J* = 6.5 Hz, 1H), 4.47–4.42 (m, 2H), 4.38–4.32 (m, 1H), 2.13 (s, 3H), 2.10 (s, 3H), 2.07 (s, 3H). ^13^C NMR (75 MHz, CDCl_3_) δ 170.8, 170.1, 169.6, 158.2, 146.7, 137.1, 129.2 (2C), 125.0, 120.1 (2C), 103.8, 75.8, 67.1, 66.9, 61.0, 20.96, 20.93, 20.8. IR (ν, cm^–1^) = 3192; 2877; 1691; 1661; 1633; 1609; 1484; 1458; 1398; 1322; 1186; 1061; 1022; 728. HRMS (ESI-TOF) calcd 430.0904 [C_19_H_21_NO_8_ + K^+^], found 430.0908.

### Procedure for Protection of C1-Amidoglycals with Benzyl Group

To a solution of the deprotected amidoglycal (**5b**, 0.09, 31 mg, 1.0 equiv) in anhydrous THF (500 μL) placed in an ice bath, NaH (0.36 mmol, 62 mg, 4.0 equiv) was added portionwise, then TBAI (0.018 mmol, 6.6 mg, 20 mol %) and benzyl bromide (0.36, 43 μL, 4 equiv) The reaction mixture was stirred for 16 h, carefully diluted with distillated H_2_O (5 mL). The aqueous layer was extracted (3 × 5 mL) with EtOAc, and the combined organic layers dried under MgSO_4_. The crude product was purified by flash column chromatography using EtOAc/Hexanes (20%) as eluent.

#### (2*R*,3*S*,4*R*)-3,4-Bis(benzyloxy)-2-((benzyloxy)methyl)-*N*-(4-bromophenyl)-3,4-dihydro-2*H*-pyran-6-carboxamide (**6b**)

The product was obtained as a pale-yellow oil (28 mg, 50%). [α]_D_^20^ = −42 (*c* = 0.1 in CHCl_3_). ^1^H NMR (300 MHz, CDCl_3_) δ 7.35–7.23 (m, 15H), 7.19–7.14 (m, 3H), 6.87 (s, 1H), 6.83 (d, *J* = 8.5 Hz, 2H), 5.70 (d, *J* = 2.8 Hz, 1H), 4.96–4.83 (m, 2H), 4.75–4.63 (m, 2H), 4.54–4.46 (m, 2H), 4.19 (dd, *J* = 6.5 Hz, 2.7 Hz, 1H), 3.72 (dd, *J* = 9.3 Hz, 6.8 Hz, 1H), 3.49 (m, 1H), 3.35 (dd, *J* = 10.0 Hz, 3,9 Hz, 1H), 3.16 (m, 1H). ^13^C NMR (75 MHz, CDCl_3_) δ 163.9, 148.8, 142.1, 138.17, 138.13, 138.12, 136.7, 128.7, 128.6, 128.5, 128.49, 128.48, 128.0, 127.9, 127.8, 127.77, 127.71, 127.10, 120.6, 106.0, 77.9, 76.3, 74.0, 73.6, 73.4, 70.6, 67.8. IR (ν, cm^–1^) = 2929; 2827; 2769; 1594; 1585; 1441; 1406; 1035; 709; 674. HRMS (ESI-TOF) calcd 636.1362 [C_34_H_32_BrNO_5_ + Na^+^], found 636.1373.

## Supporting Information Available

NMR spectra and theoretical computational (PDF)
