# Supplementary material for: 1-Iodoglycal: A Versatile Intermediate for the Synthesis of d-Glyco Amides and Esters Employing Carbonylative Cross-Coupling Reaction
Source: ACS Omega. 2024 Jul 11;9(29):31732–44. doi: 10.1021/acsomega.4c02645 (PMC11270555; doi:10.1021/acsomega.4c02645)
Supplement: Supplementary file 1 — ao4c02645_si_001.pdf [file ao4c02645_si_001.pdf]

1-Iodoglycal: A Versatile Intermediate for the Synthesis of D-glyco Amides and Esters Employing Carbonylative Cross-Coupling Reaction

Milene M. Hornink,[a] Monica F. Z. J. Toledo,[a] Daniel C. Pimenta,[b] Caio Paschoalin,[c] Pamela M. Silva,[d] Giuseppe E. Figlino,[d] Eurípedes Aguiar,[a] Gustavo Cervi,[c] Francisco W. M. Ribeiro,[c] Thiago Carita Correra,[c] Angélique Ferry,[e,f,g] Hélio A. Stefani[a]*

[a]Departamento de Farmácia, Faculdade de Ciências Farmacêuticas, Universidade de São Paulo, São Paulo, SP-Brasil. [b]Instituto Butantan, São Paulo, SP-Brasil. [c]Instituto de Química, Universidade de São Paulo, São Paulo, SP-Brasil. [d]Centro Universitário São Camilo, São Paulo, SP-Brasil. [e]Université Paris-Saclay, CNRS, BioCIS, 91400, Orsay, France. [f]CY Cergy-Paris Université, BioCIS, CNRS, 5 mail Gay-Lussac, 95000 Cergy-Pontoise cedex, France. [g]Institut Universitaire de France (IUF)

***Corresponding Author**: hstefani@usp.br

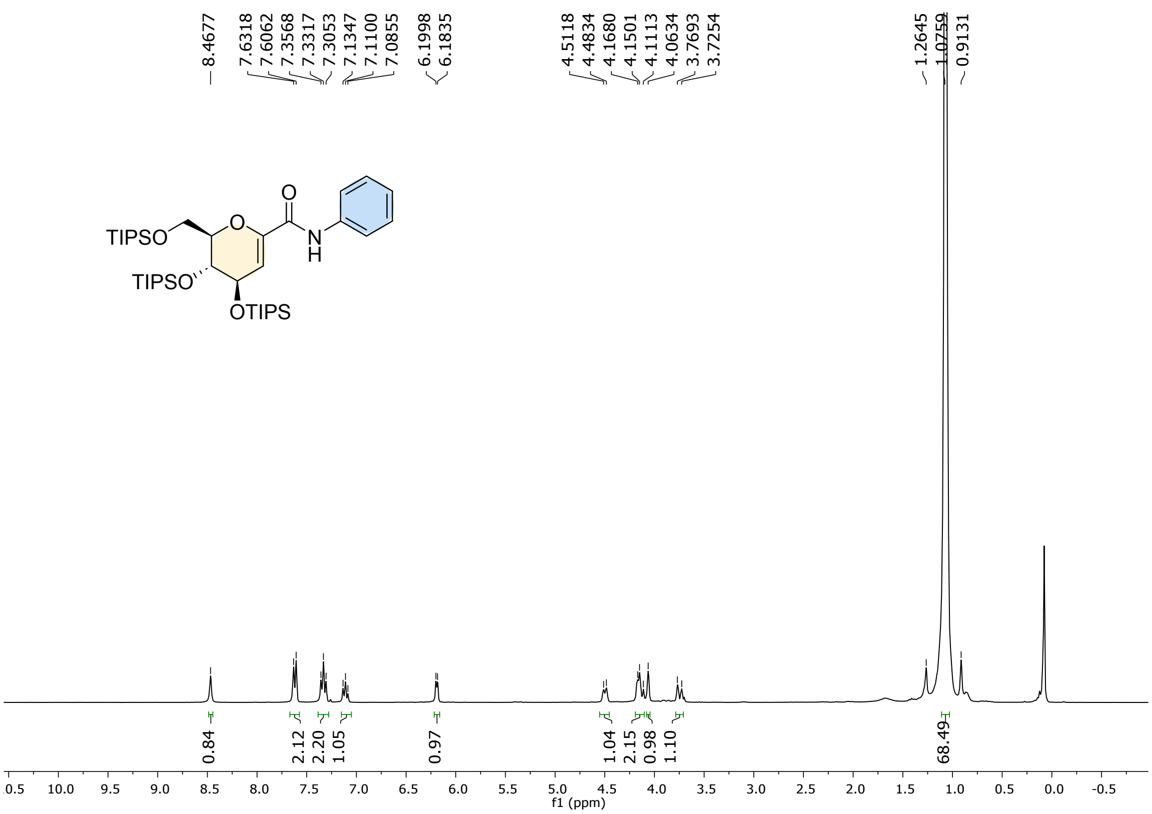

Figure S1. ^{1}H NMR spectra (300 MHz, $CDCl_3$) of **3a**

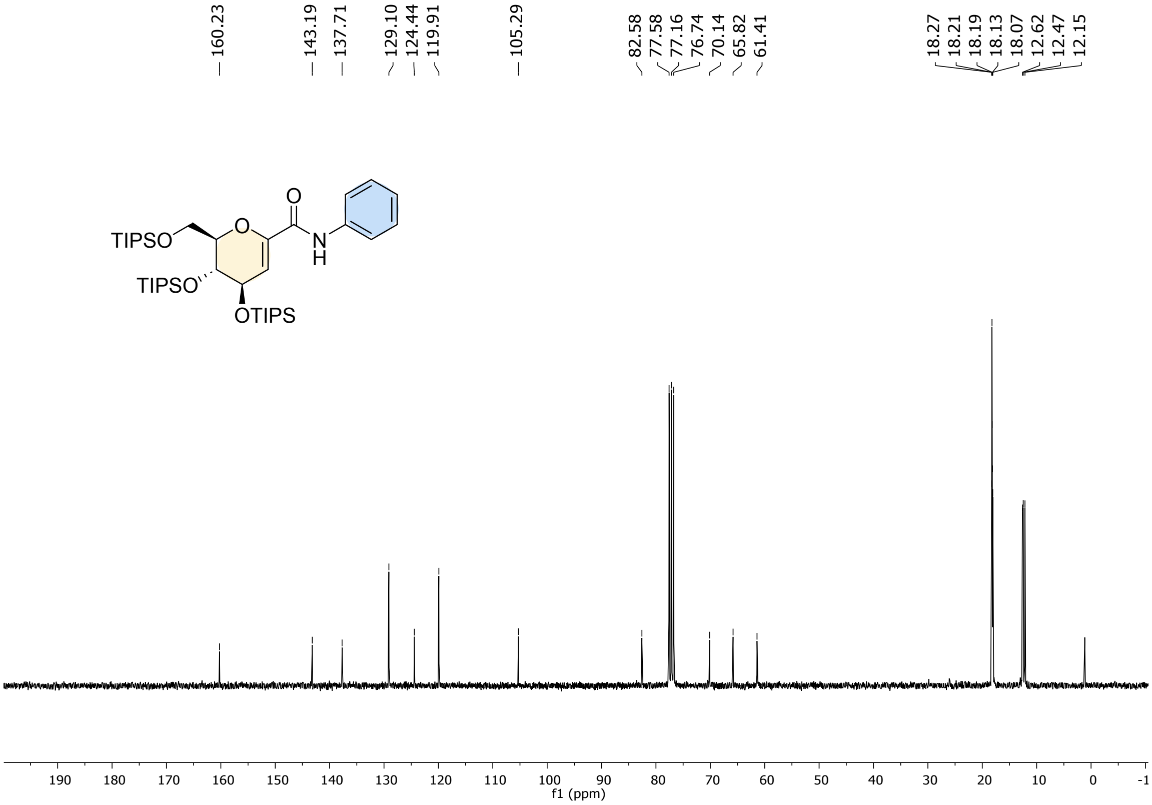

Figure S2. ^{13}C NMR spectra (75 MHz, $CDCl_3$) of **3a**

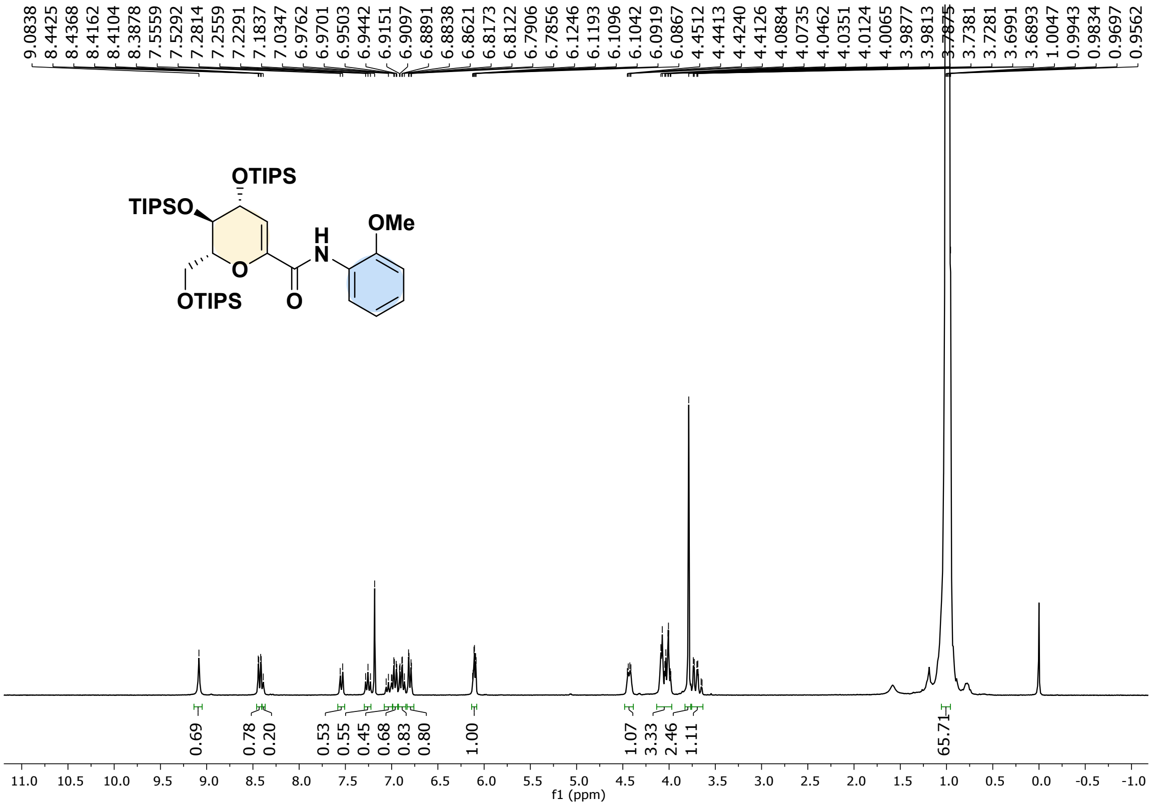

Figure S3. ^{1}H NMR spectra (300 MHz, $CDCl_3$) of **3b**

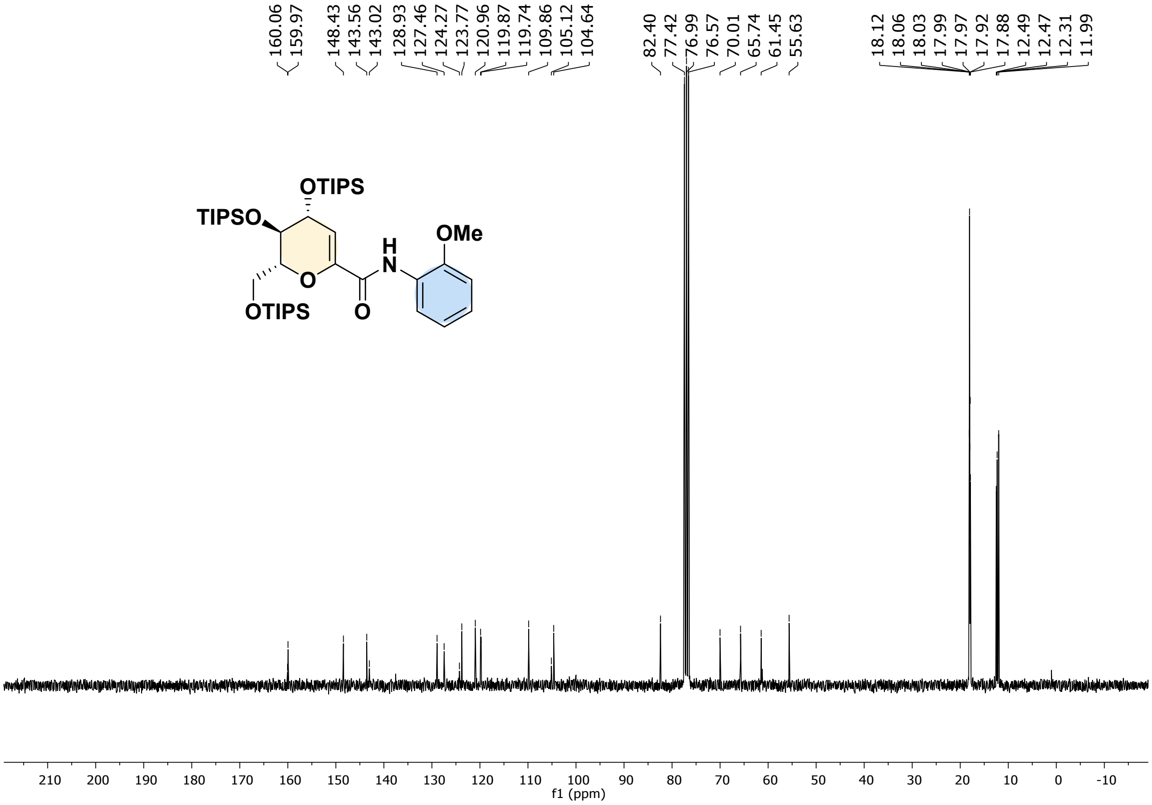

Figure S4. ^{13}C NMR spectra (75 MHz, $CDCl_3$) of **3b**

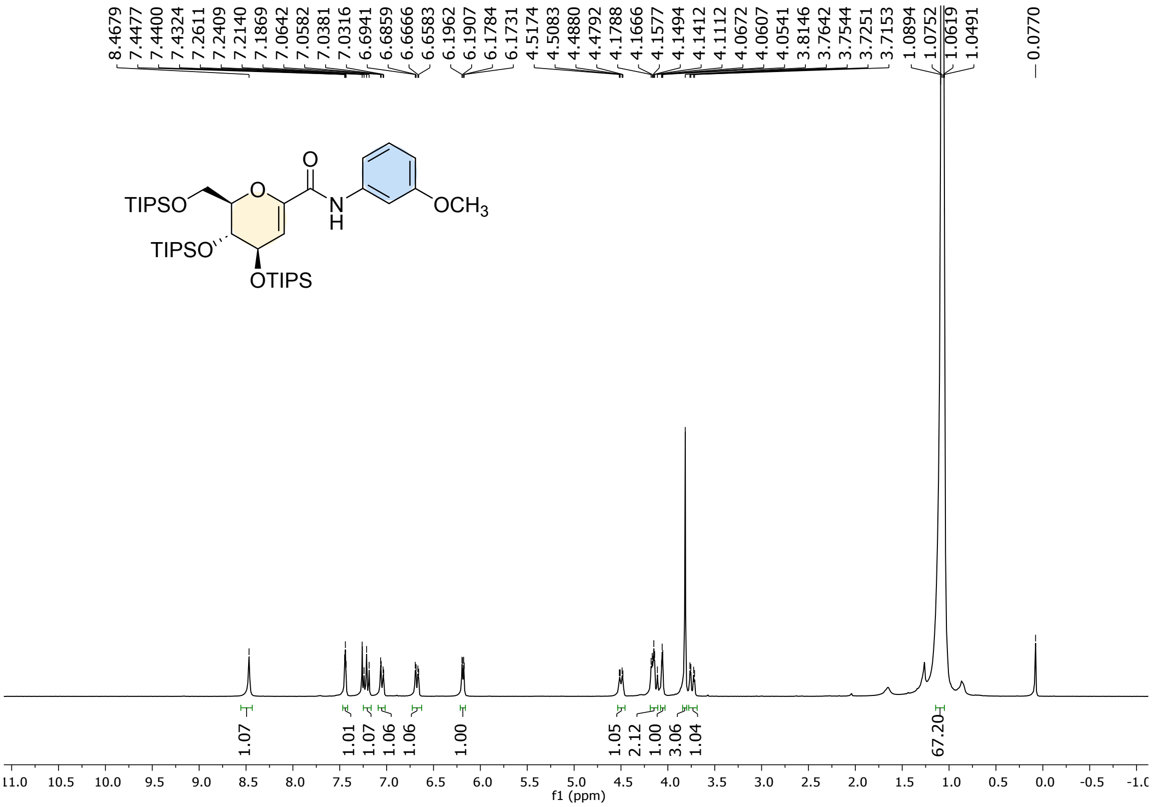

Figure S5. ^{1}H NMR spectra (300 MHz, $CDCl_3$) of **3c**

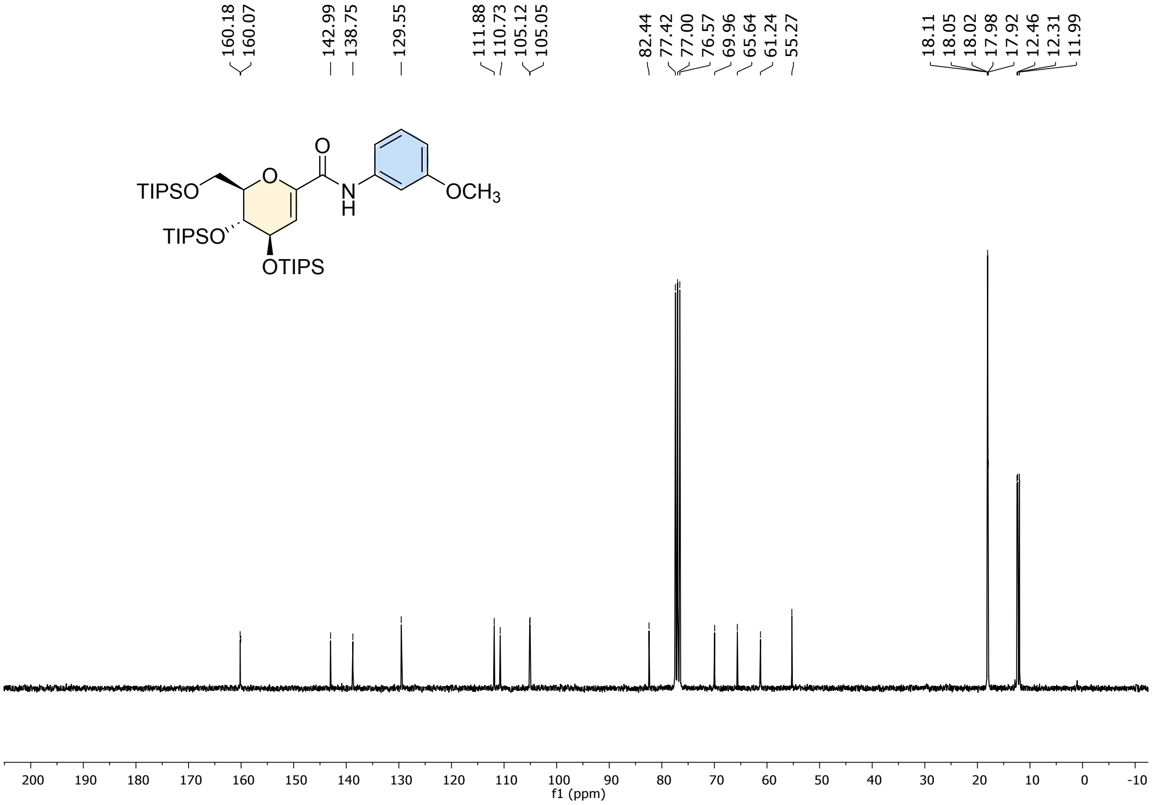

Figure S6. ^{13}C NMR spectra (75 MHz, $CDCl_3$) of **3c**

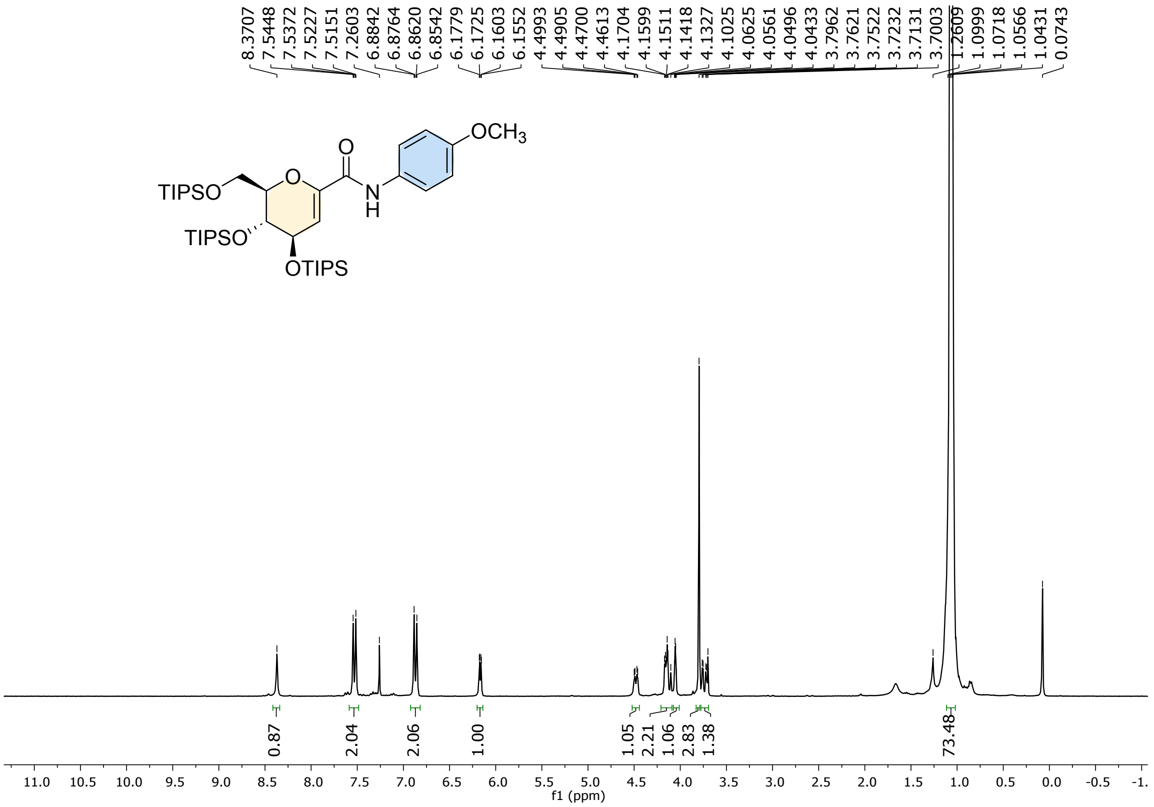

Figure S7. ^{1}H NMR spectra (300 MHz, $CDCl_3$) of **3d**

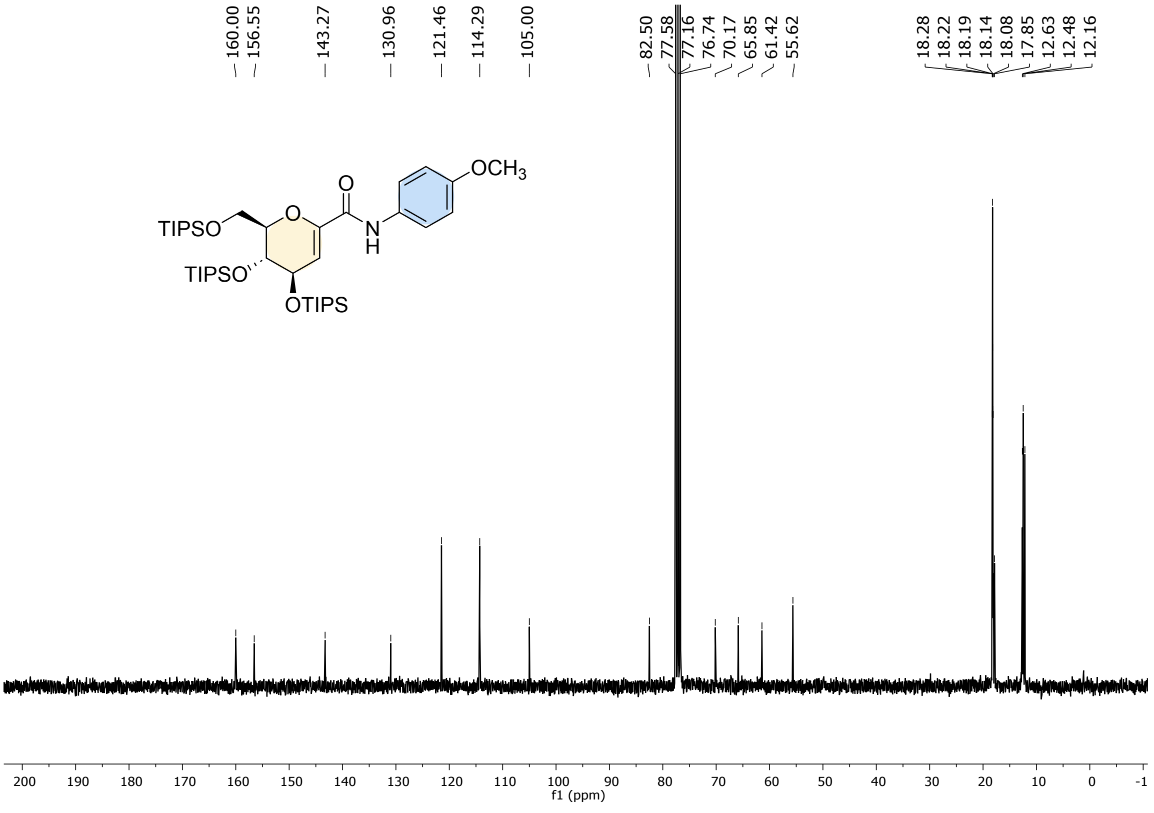

Figure S8. ^{13}C NMR spectra (75 MHz, $CDCl_3$) of **3d**

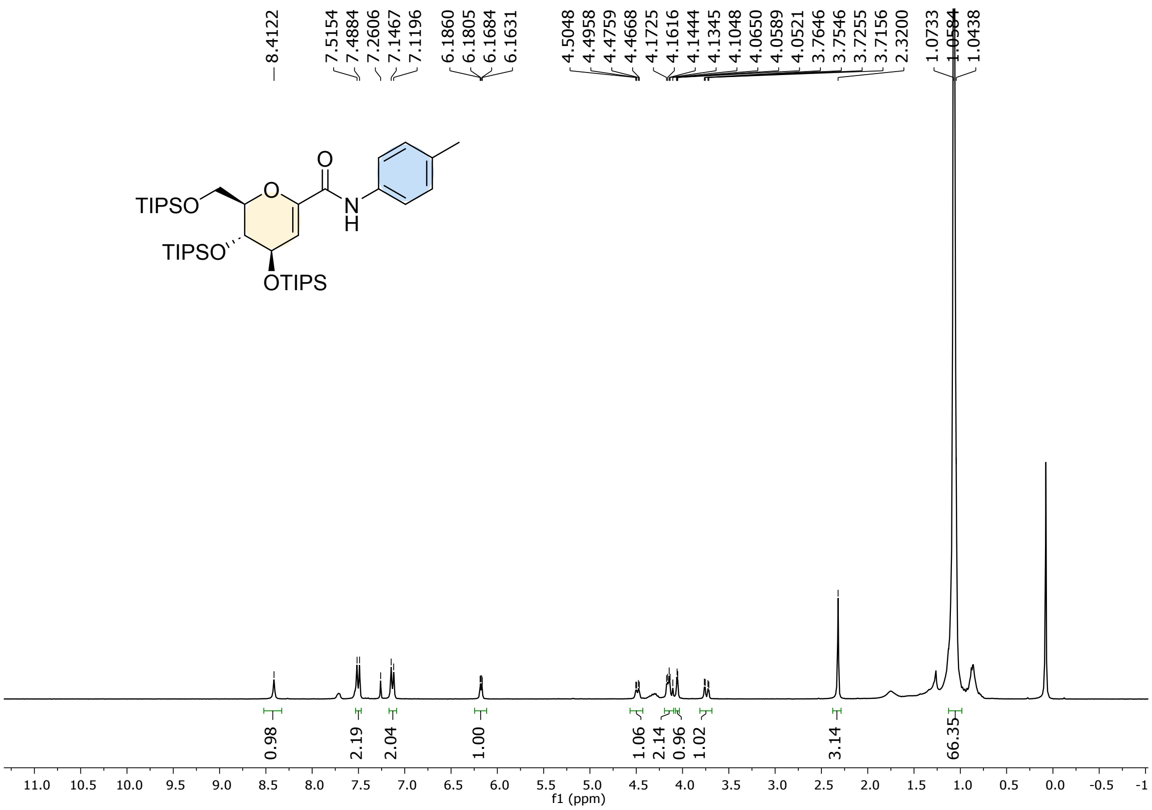

Figure S9. ^{1}H NMR spectra (300 MHz, $CDCl_3$) of **3e**

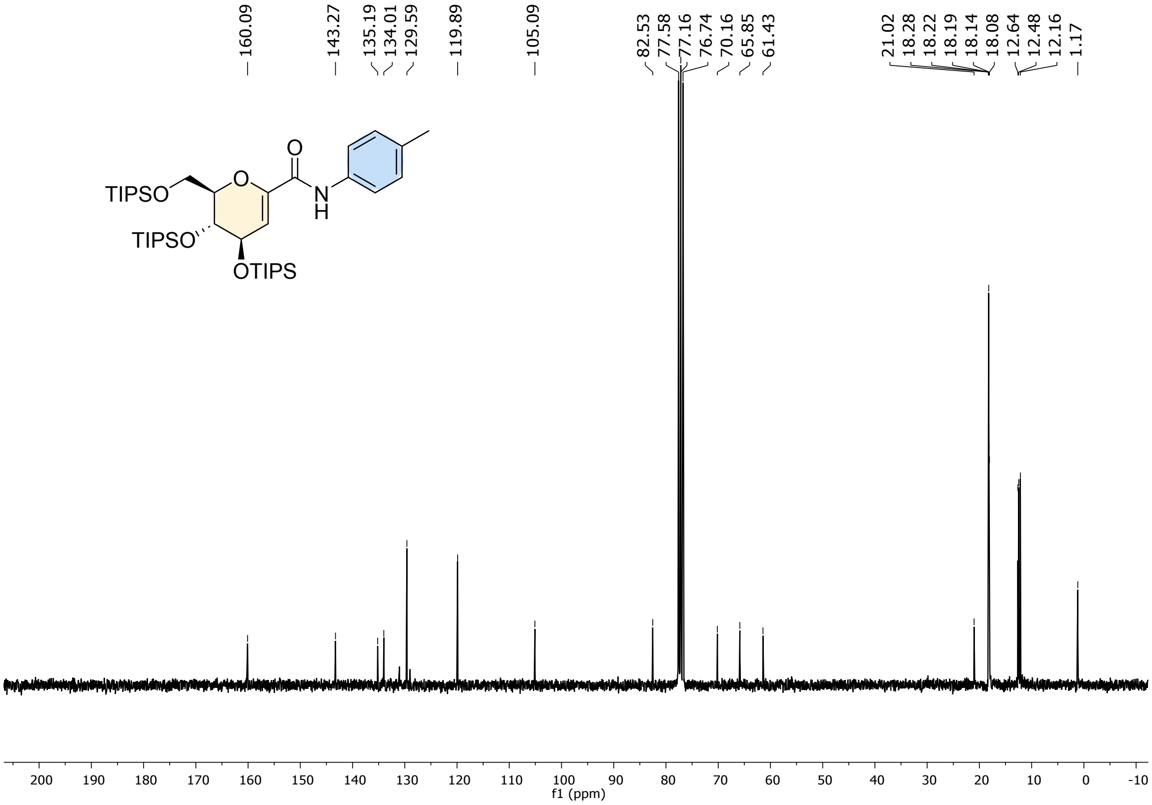

Figure S10. ^{13}C NMR spectra (75 MHz, $CDCl_3$) of **3e**

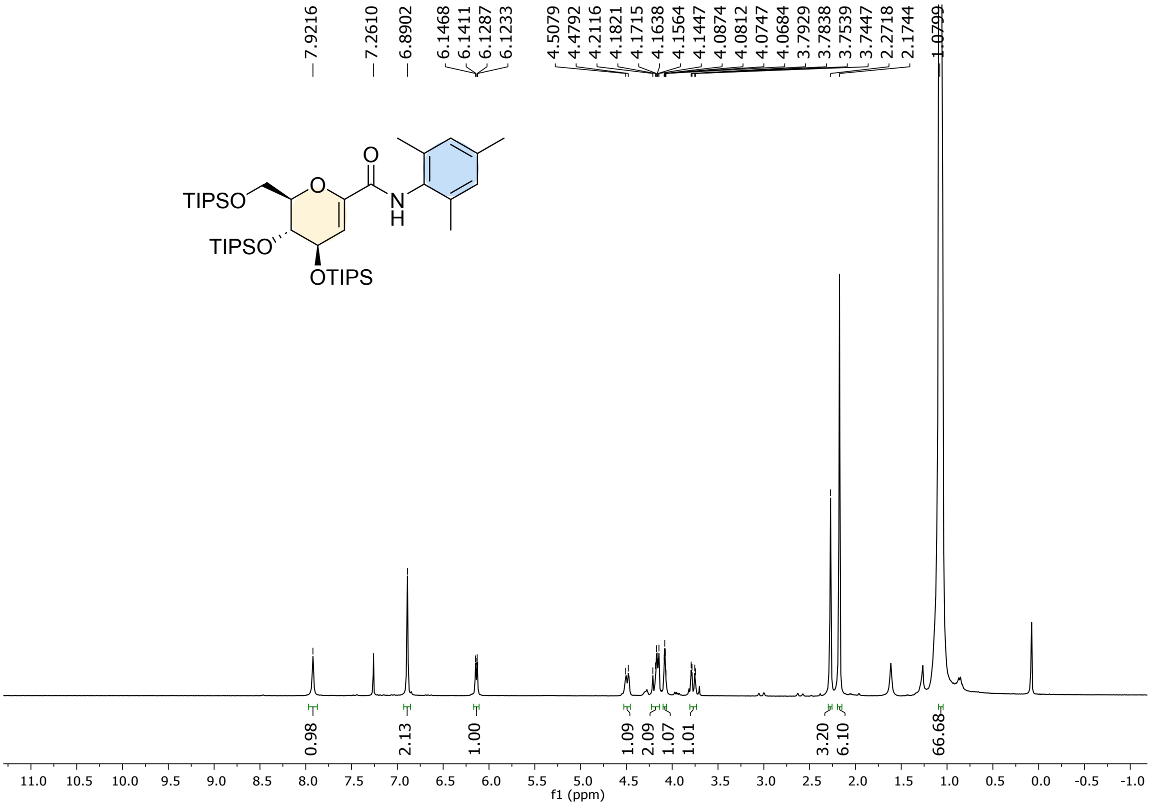

Figure S11. ^{1}H NMR spectra (300 MHz, $CDCl_3$) of **3f**

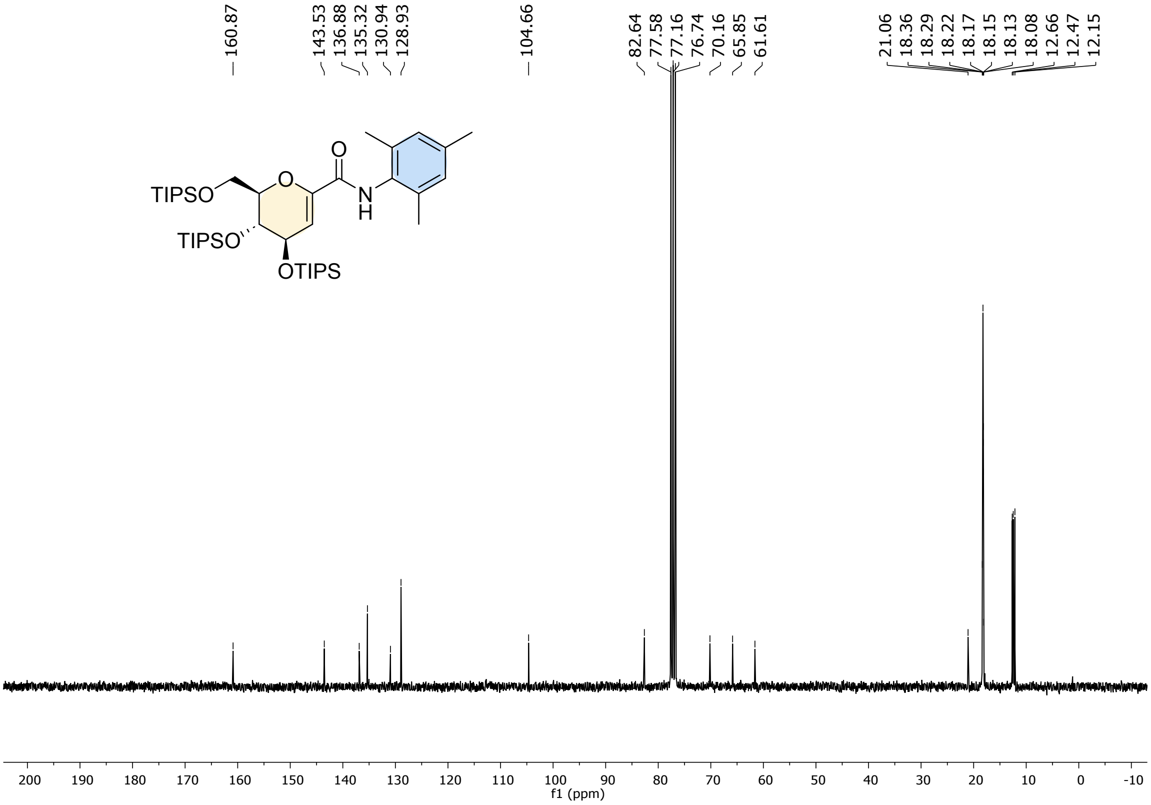

Figure S12. ^{13}C NMR spectra (75 MHz, $CDCl_3$) of **3f**

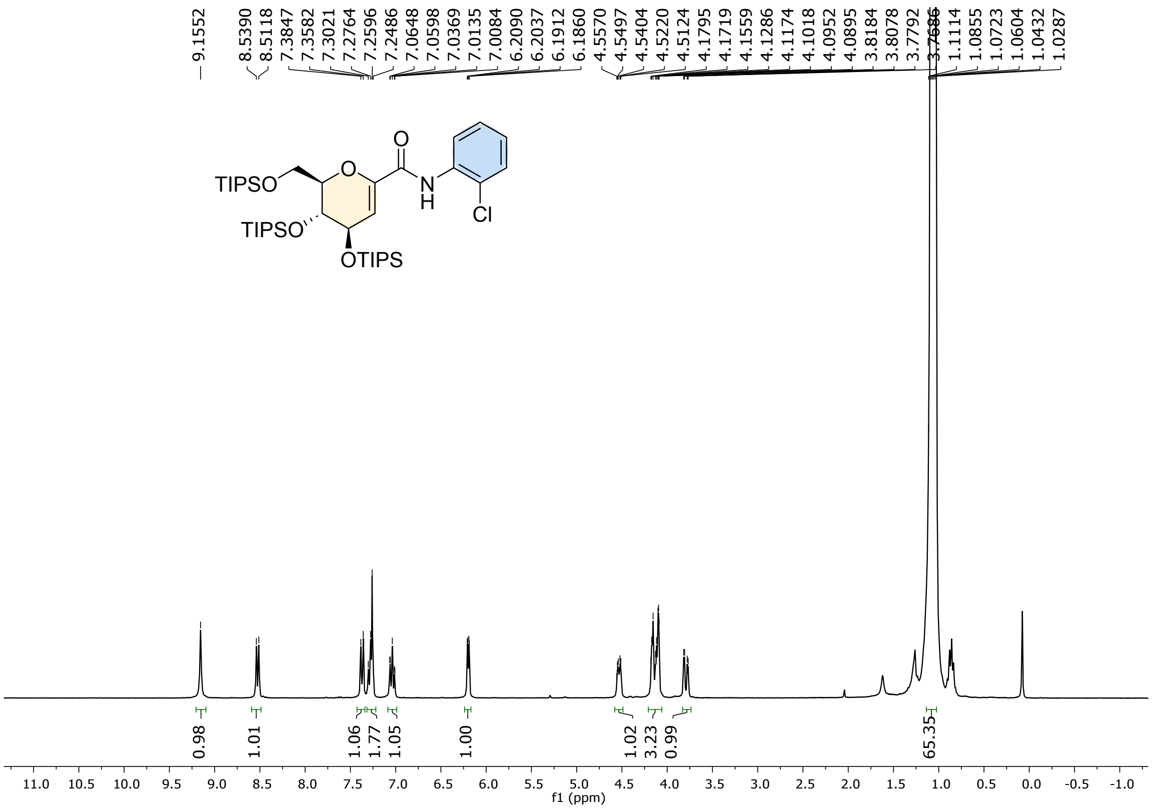

Figure S13. ^{1}H NMR spectra (300 MHz, $CDCl_3$) of **3g**

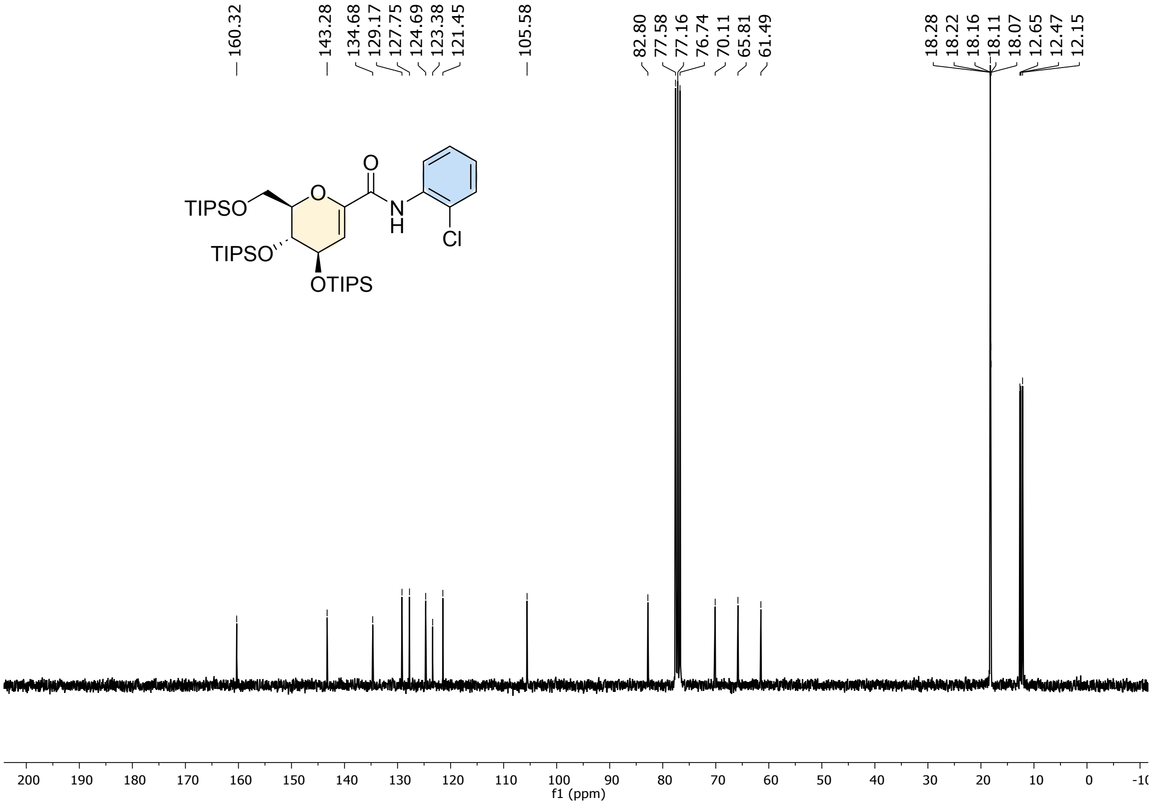

Figure S14. ^{13}C NMR spectra (75 MHz, $CDCl_3$) of **3g**

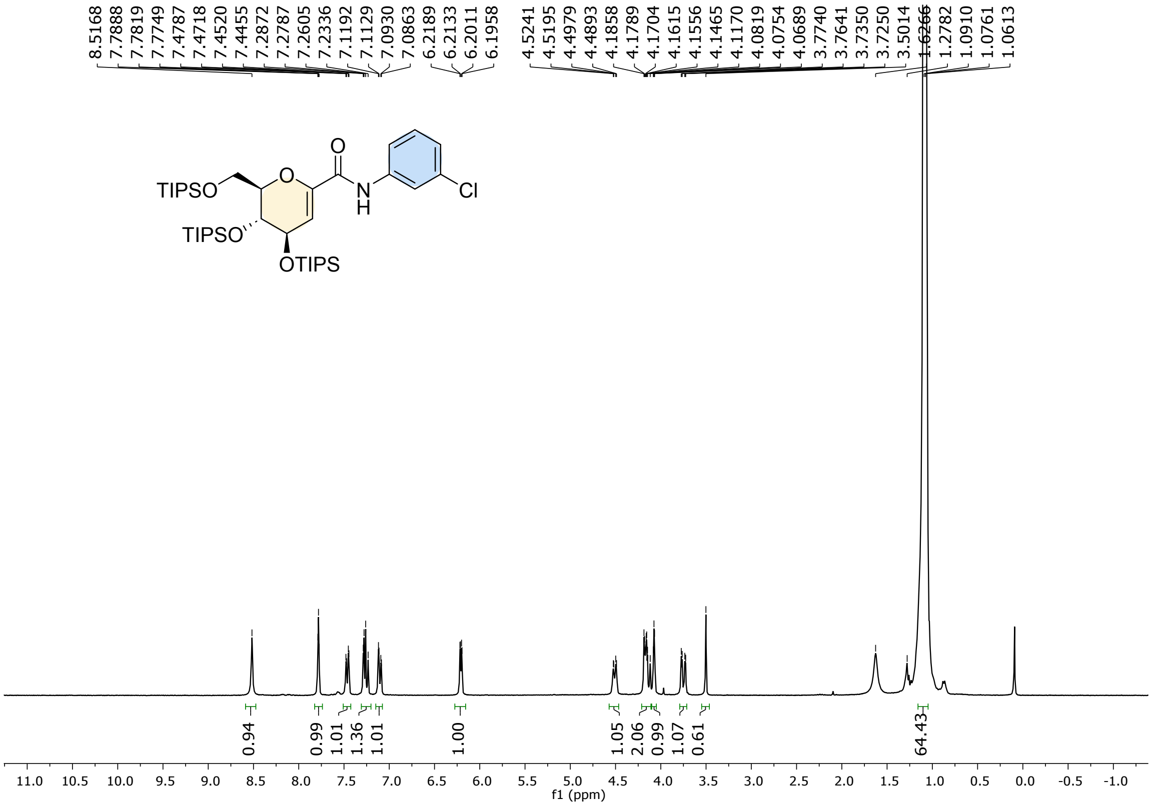

Figure S15. ^{1}H NMR spectra (300 MHz, $CDCl_3$) of **3h**

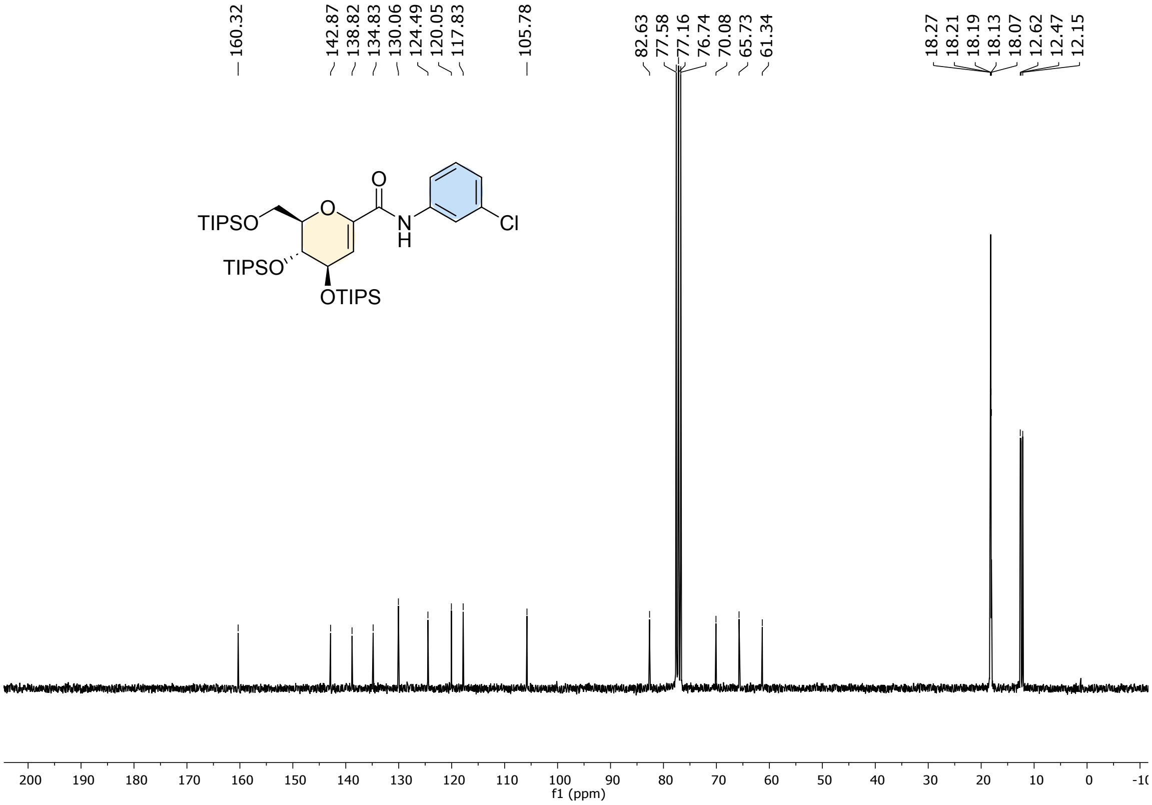

Figure S16. ^{13}C NMR spectra (75 MHz, $CDCl_3$) of **3h**

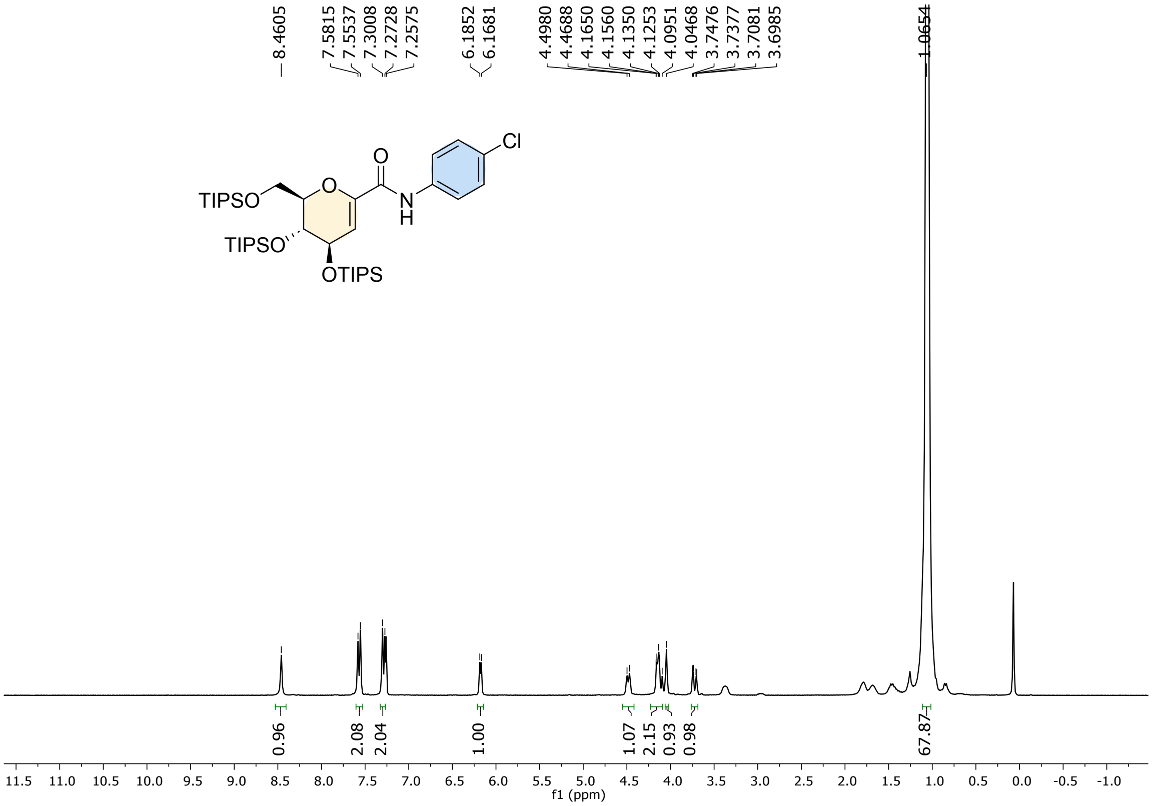

Figure S17. ^{1}H NMR spectra (300 MHz, $CDCl_3$) of **3i**

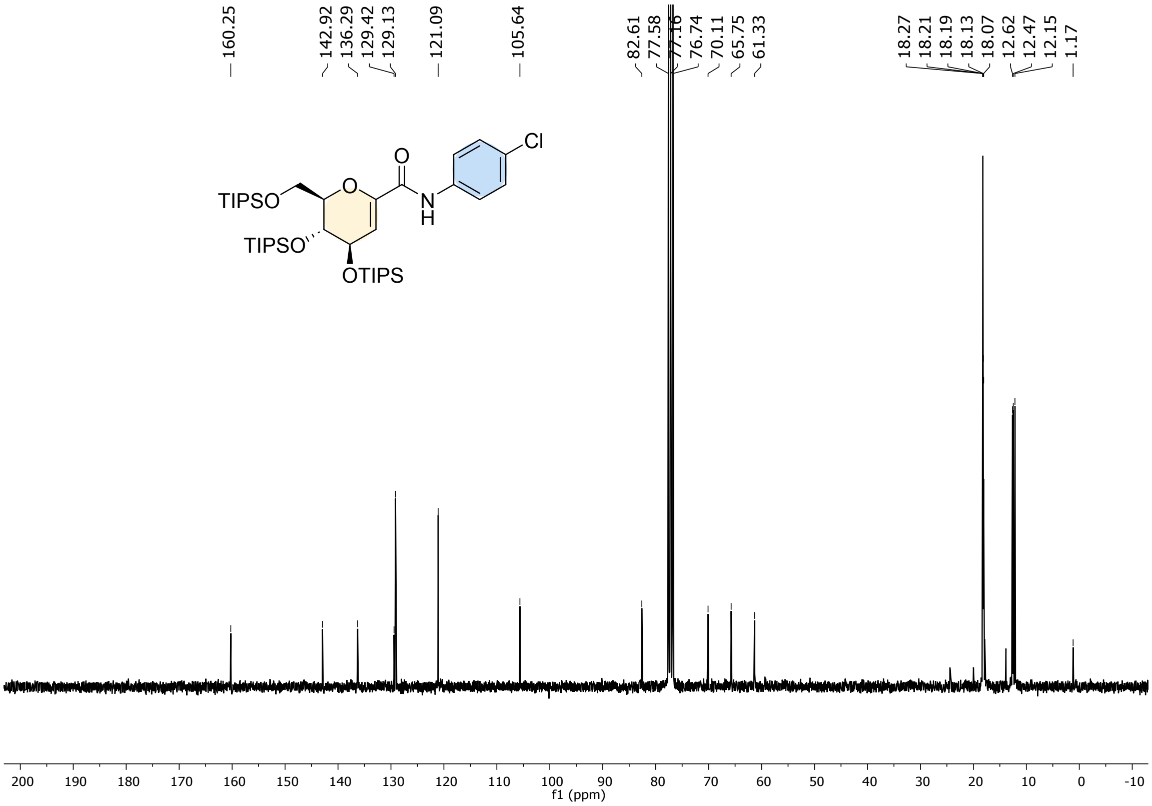

Figure S18. ^{13}C NMR spectra (75 MHz, $CDCl_3$) of **3i**

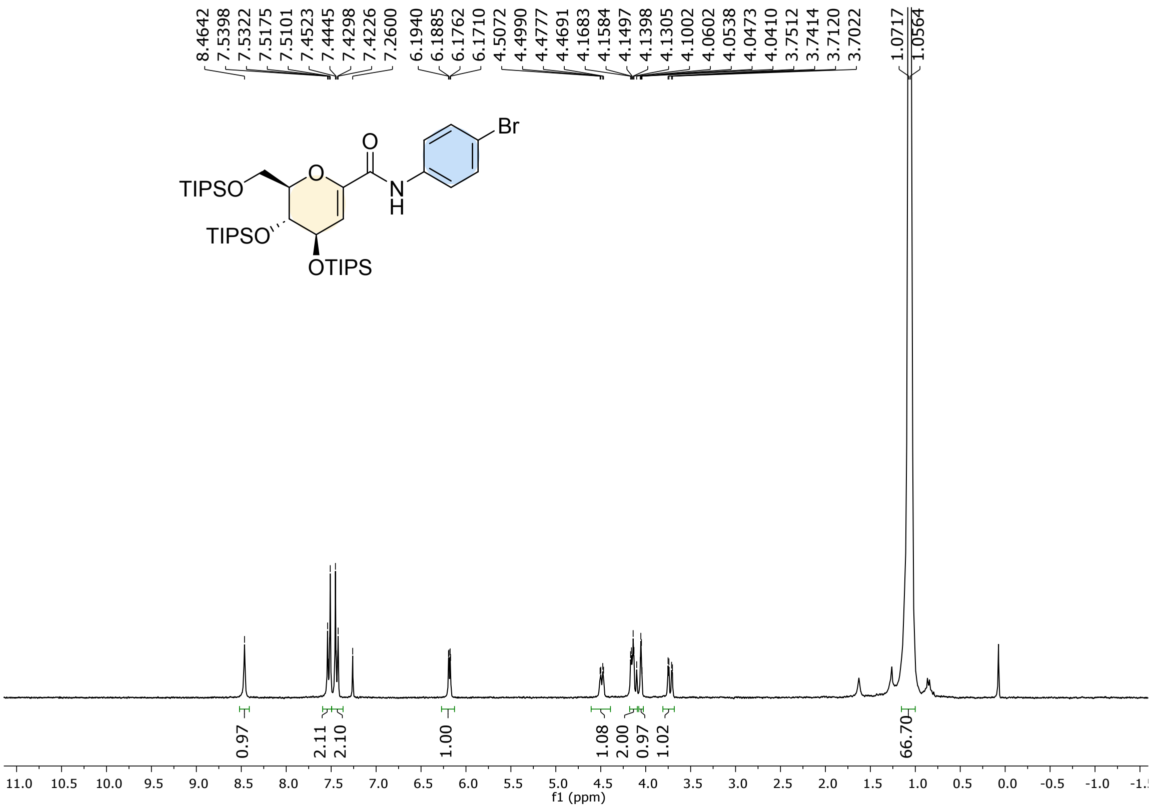

Figure S19. ^{1}H NMR spectra (300 MHz, $CDCl_3$) of **3j**

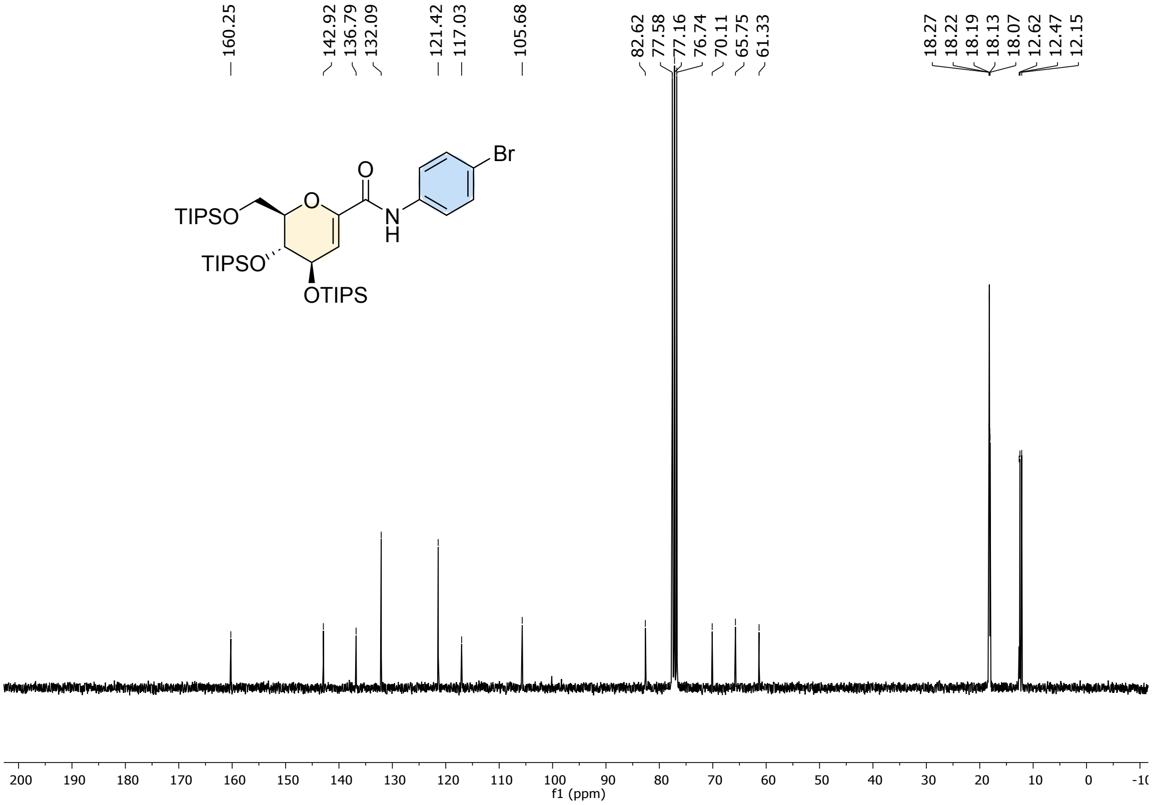

Figure S20. ^{13}C NMR spectra (75 MHz, $CDCl_3$) of **3j**

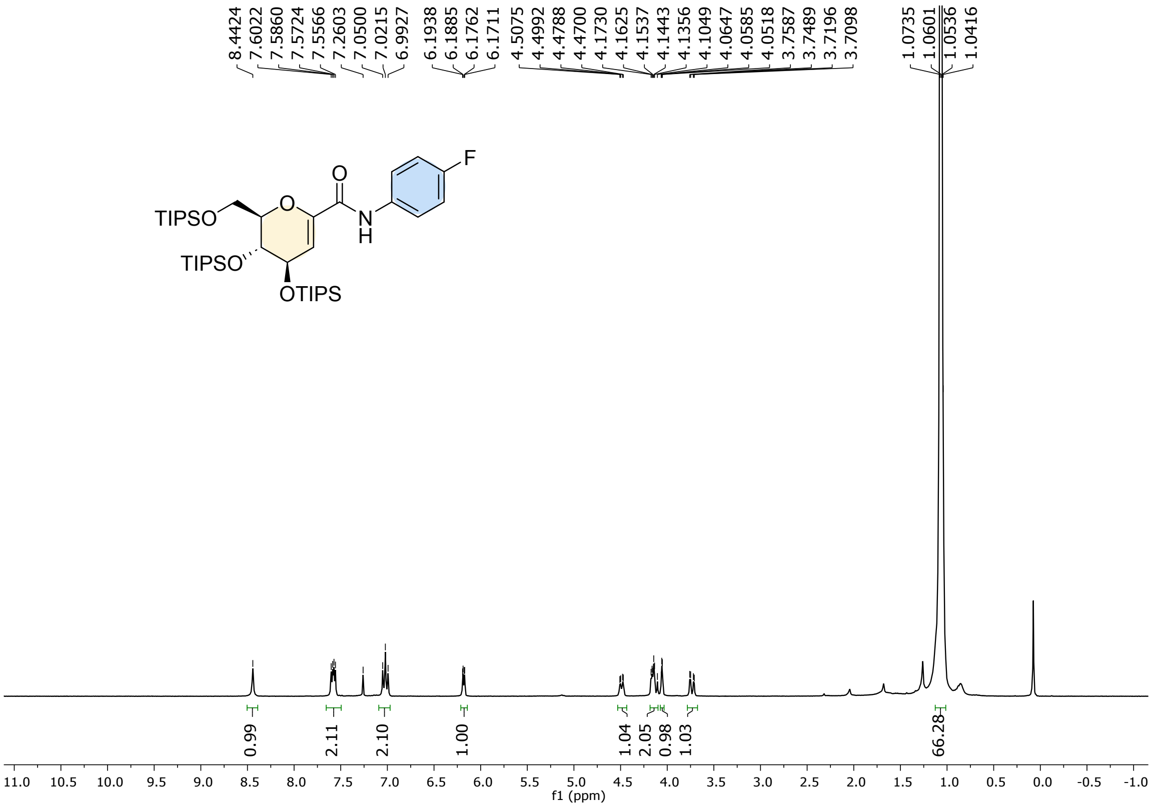

Figure S21. ^{1}H NMR spectra (300 MHz, $CDCl_3$) of **3k**

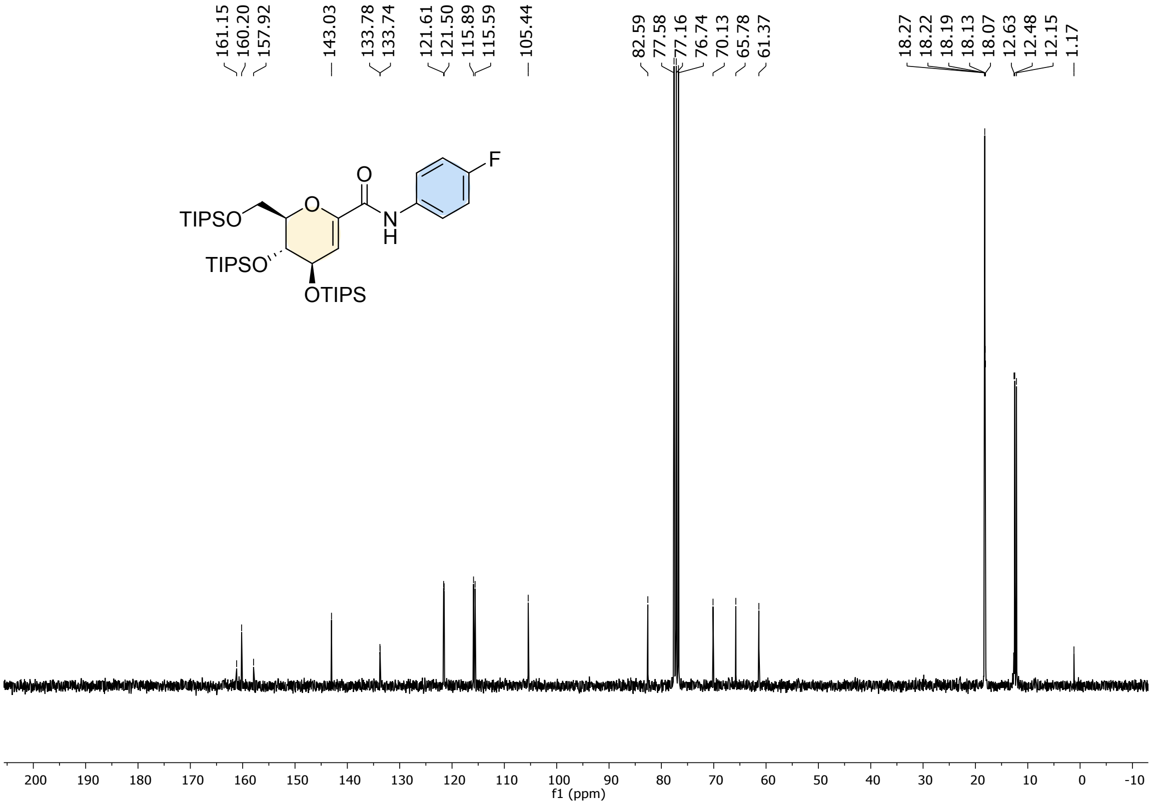

Figure S22. ^{13}C NMR spectra (75 MHz, $CDCl_3$) of **3k**

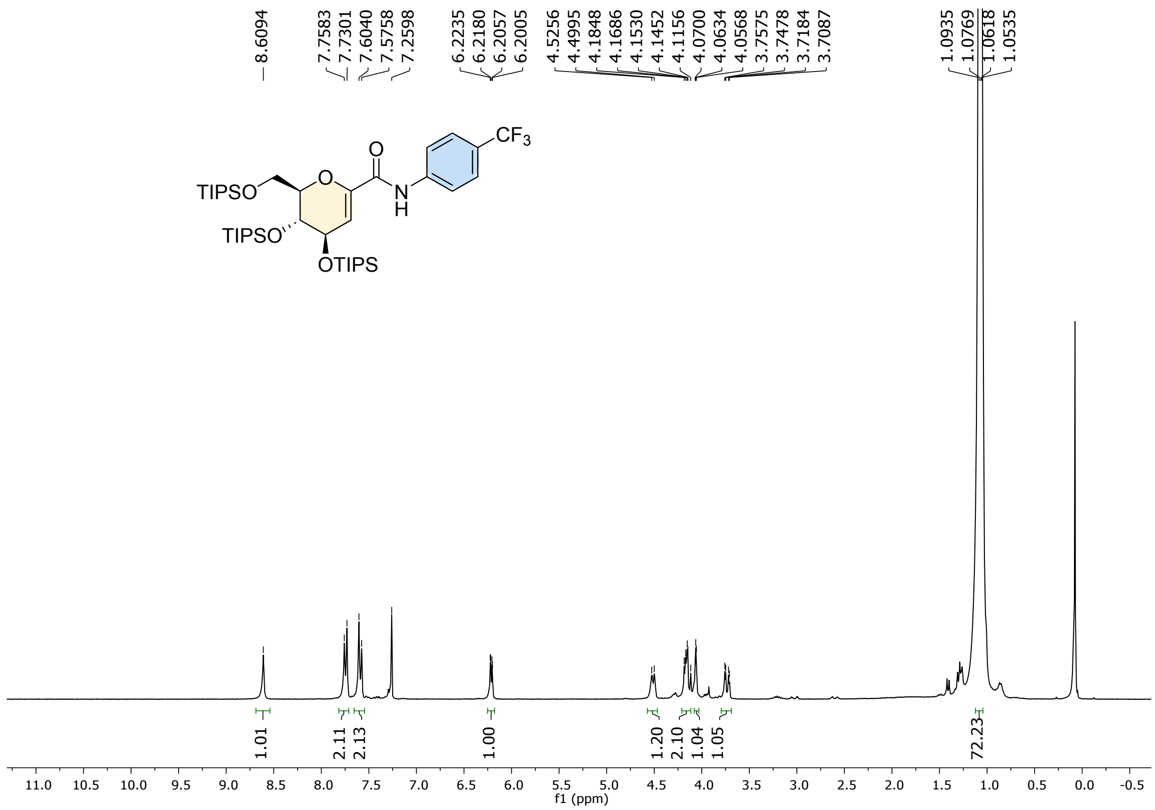

Figure S23. ^{1}H NMR spectra (300 MHz, $CDCl_3$) of **3l**

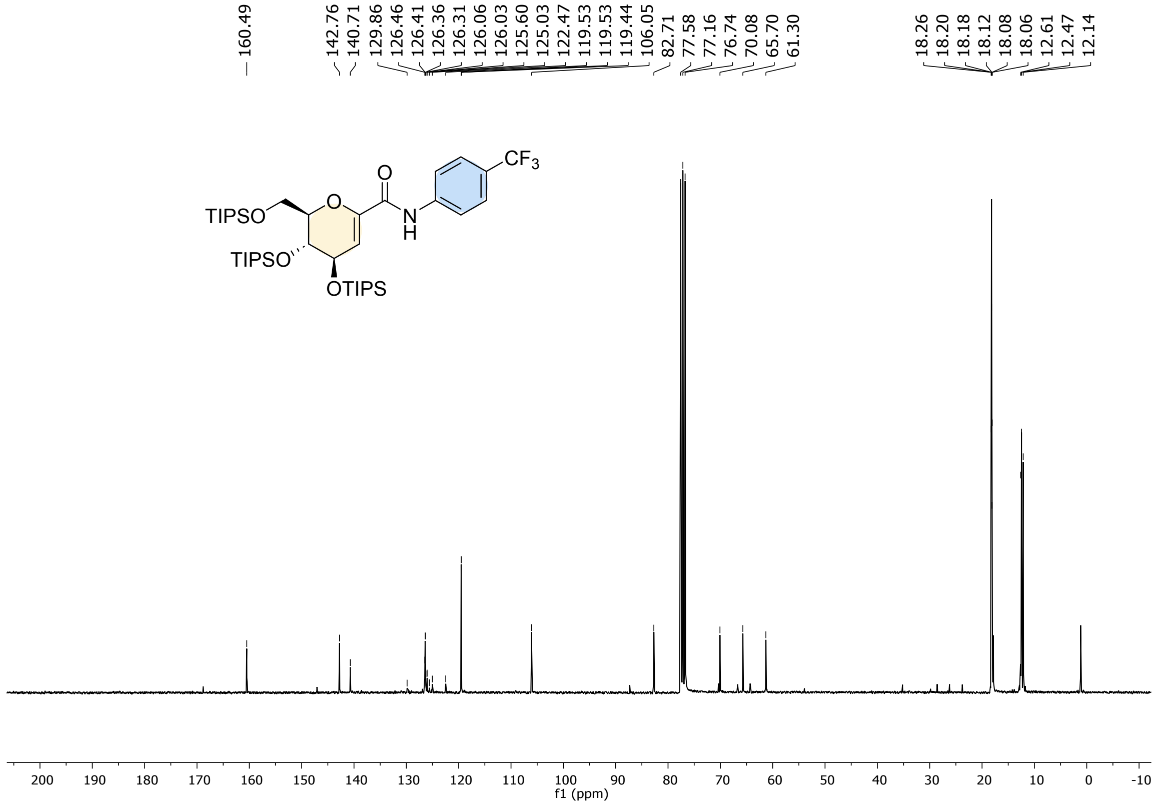

Figure S24. ^{13}C NMR spectra (75 MHz, $CDCl_3$) of **3l**

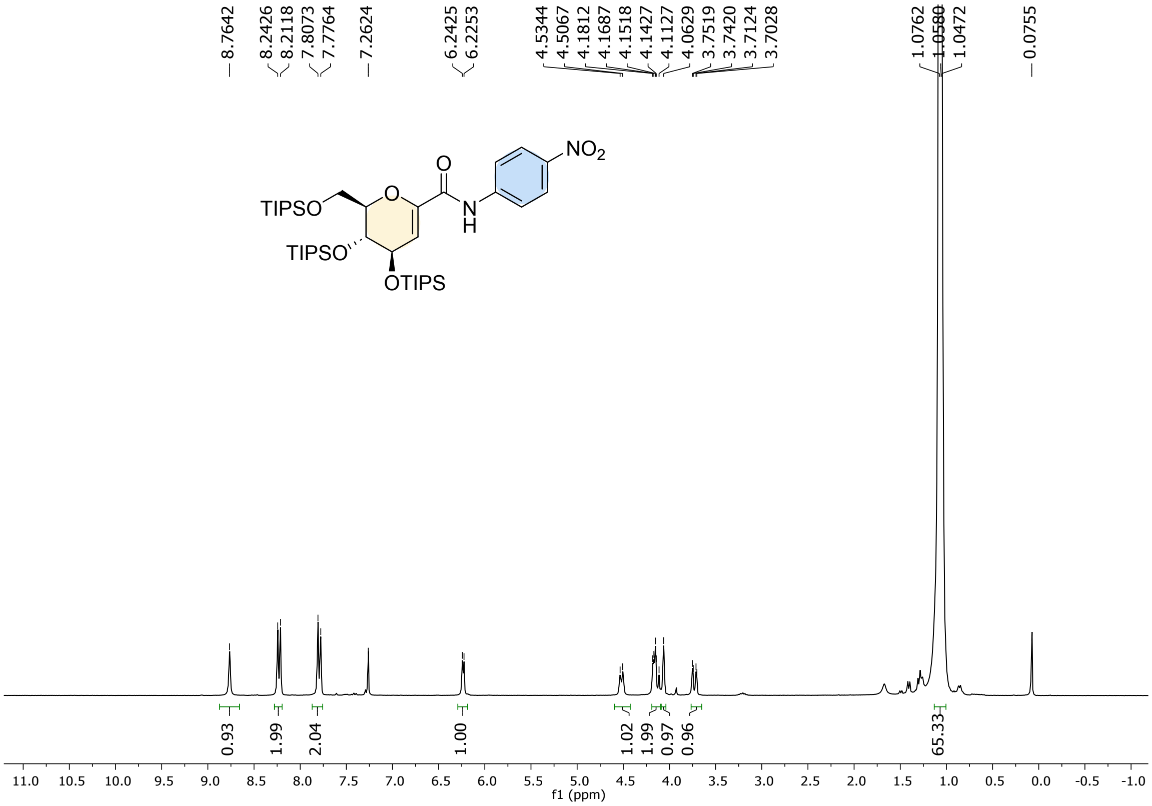

Figure S25. ^{1}H NMR spectra (300 MHz, $CDCl_3$) of **3m**

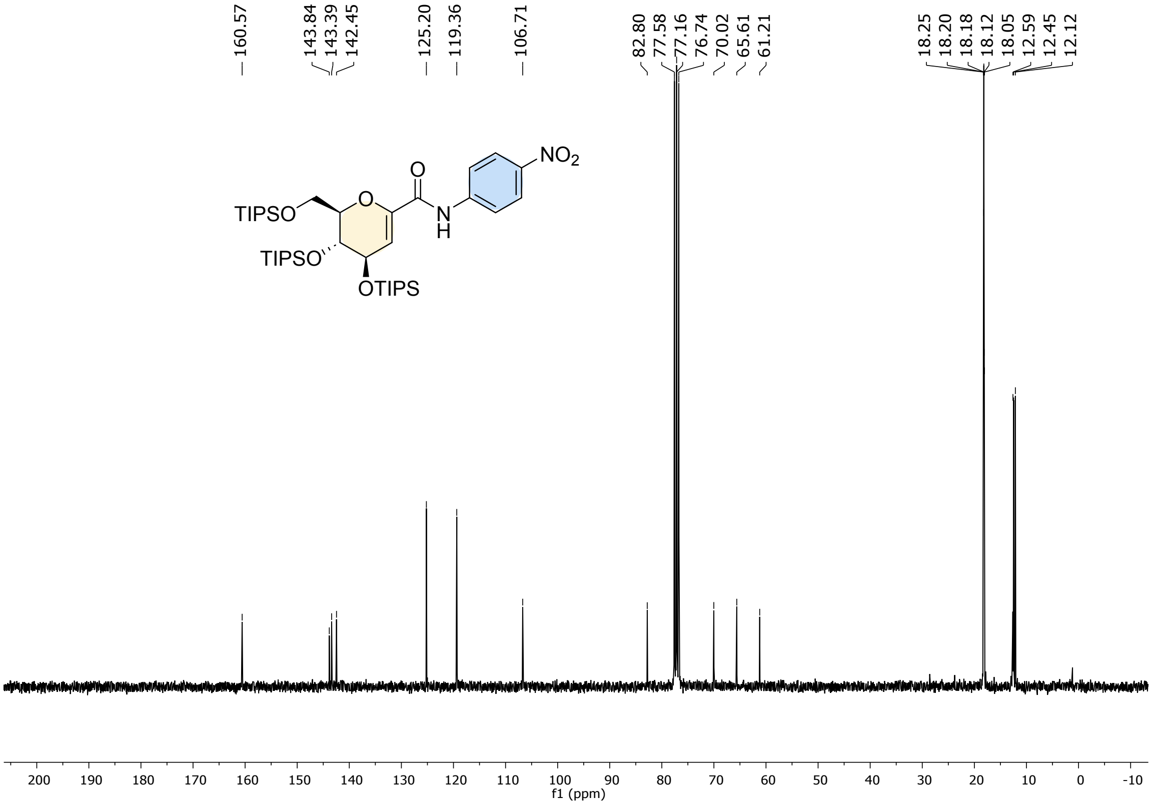

Figure S26. ^{13}C NMR spectra (75 MHz, $CDCl_3$) of **3m**

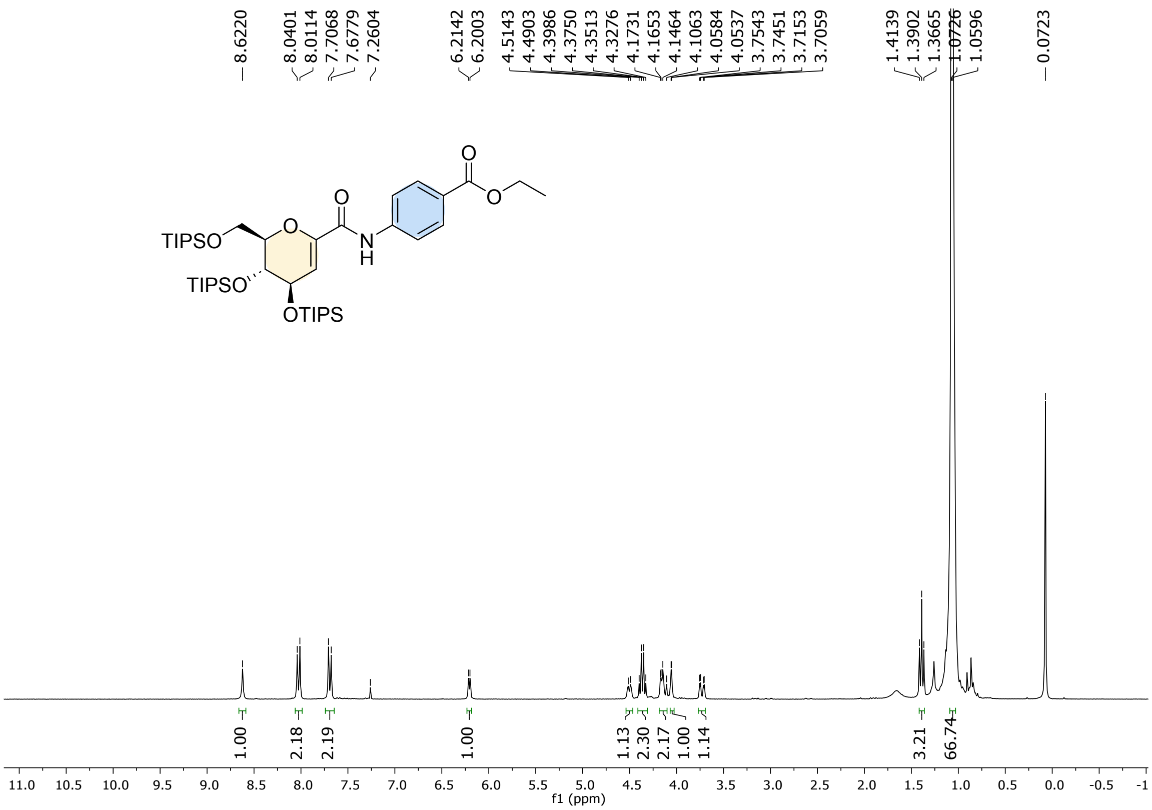

Figure S27. ^{1}H NMR spectra (300 MHz, $CDCl_3$) of **3n**

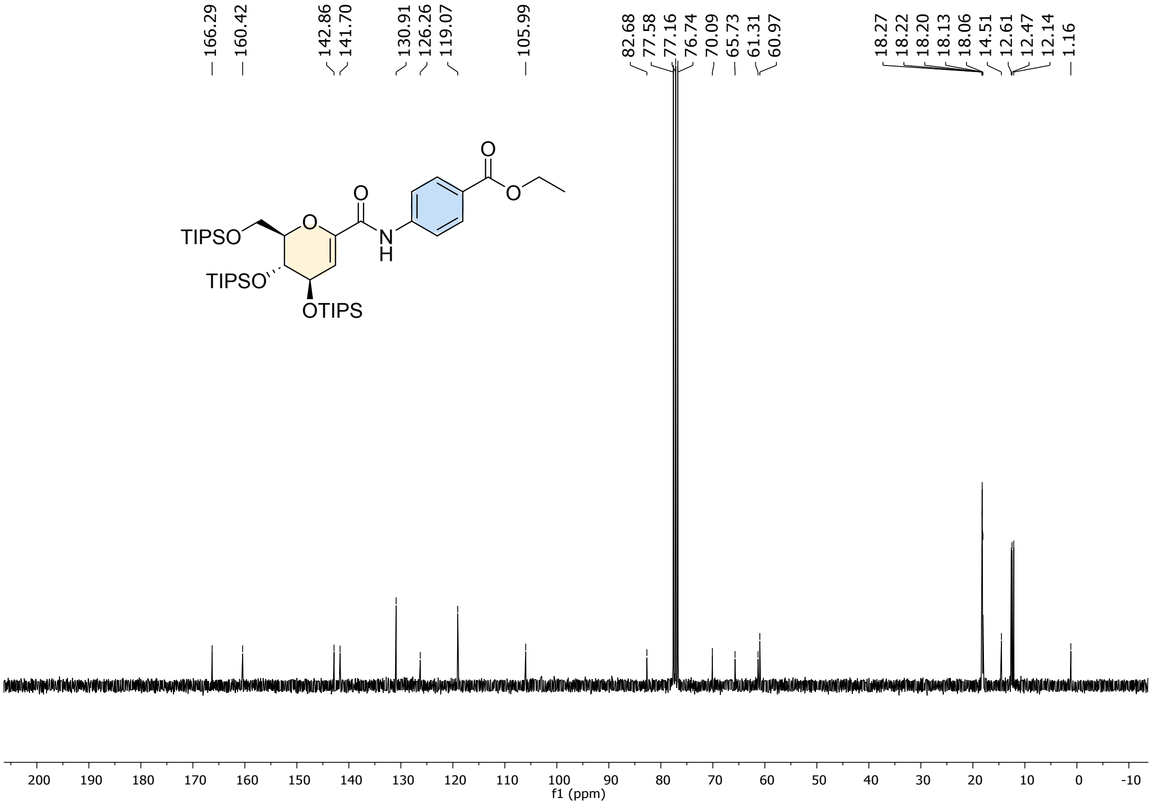

Figure S28. ^{13}C NMR spectra (75 MHz, $CDCl_3$) of **3n**

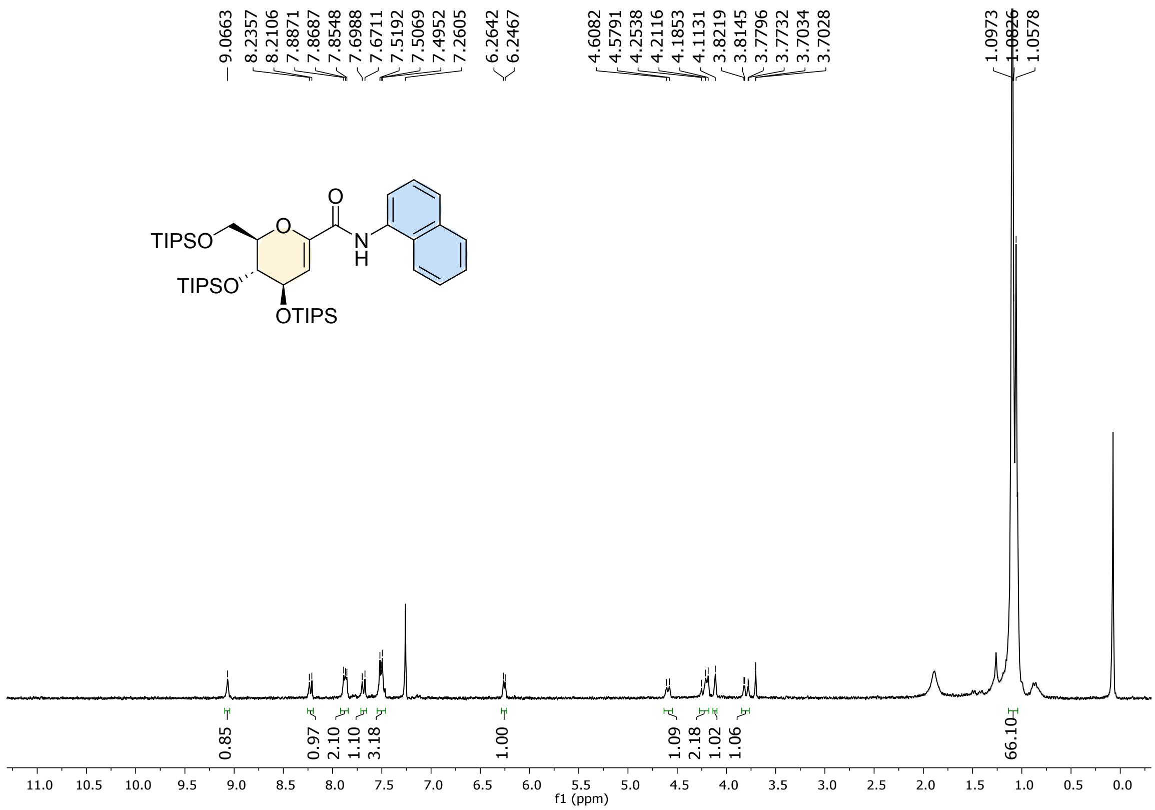

Figure S29. ^{1}H NMR spectra (300 MHz, $CDCl_3$) of **3o**

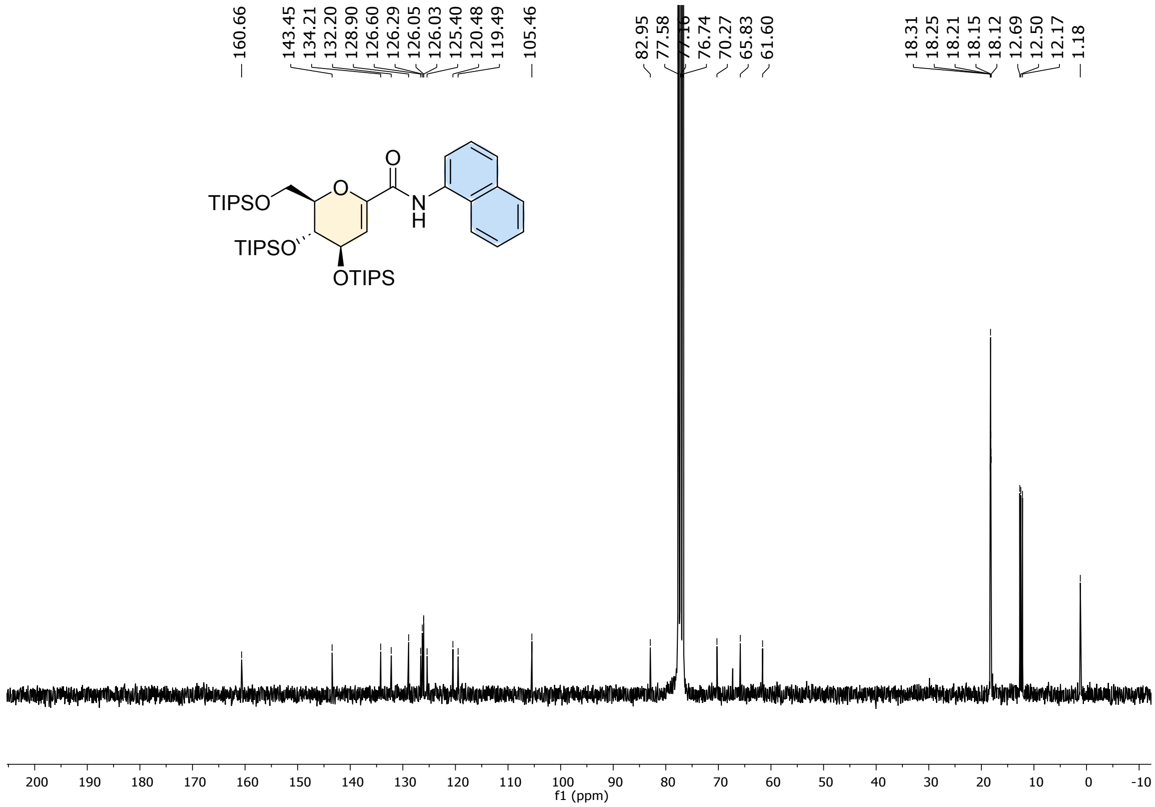

Figure S30. ^{13}C NMR spectra (75 MHz, $CDCl_3$) of **3o**

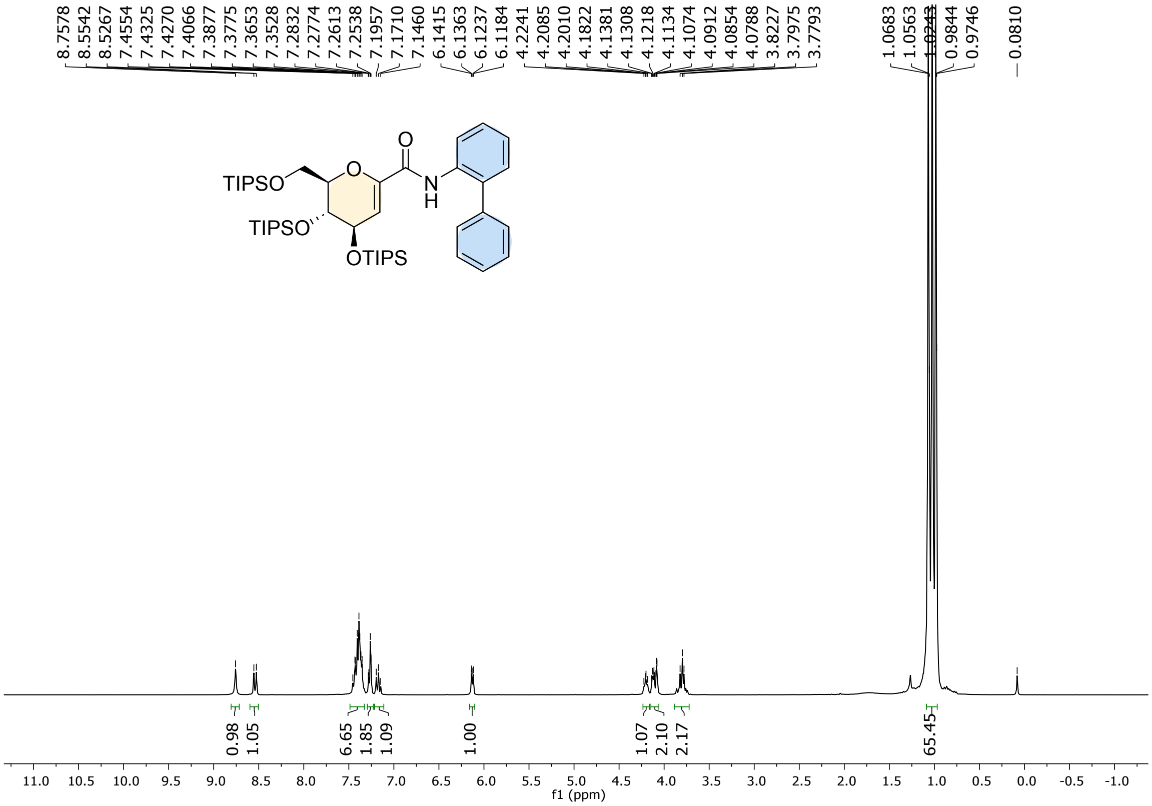

Figure S31. ^{1}H NMR spectra (300 MHz, $CDCl_3$) of **3p**

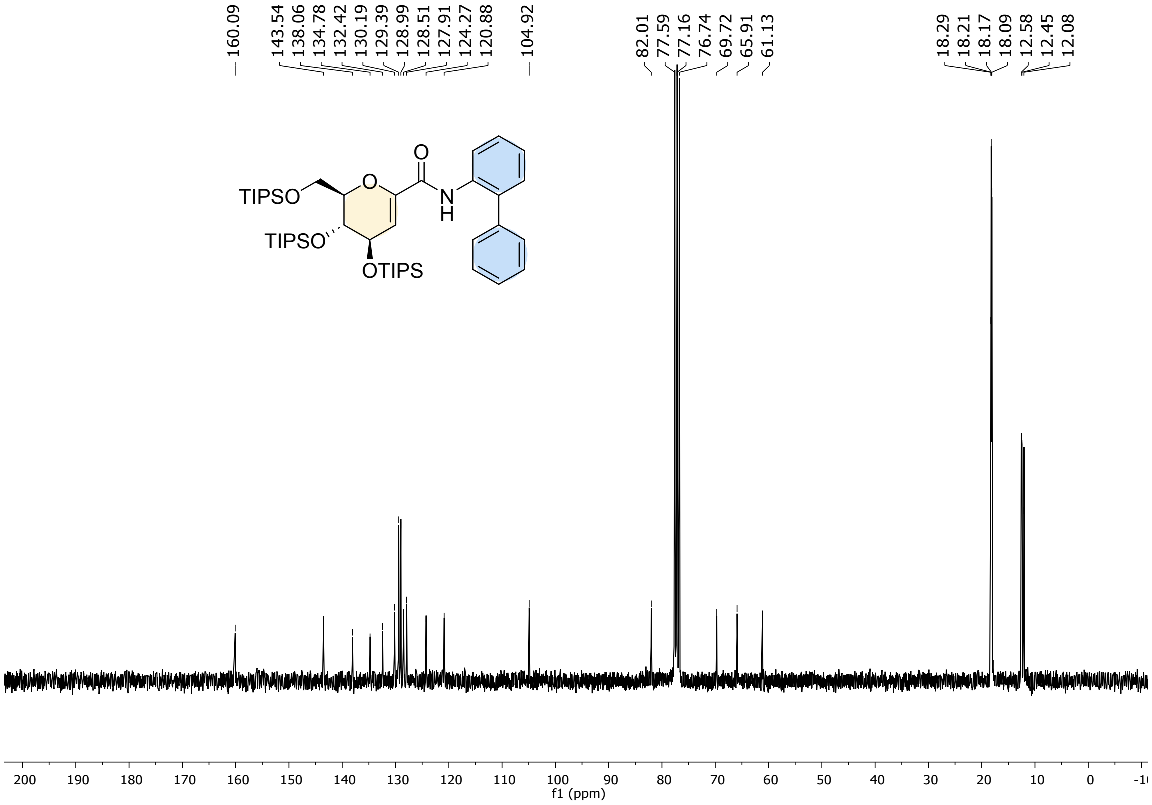

Figure S32. ^{13}C NMR spectra (75 MHz, $CDCl_3$) of **3p**

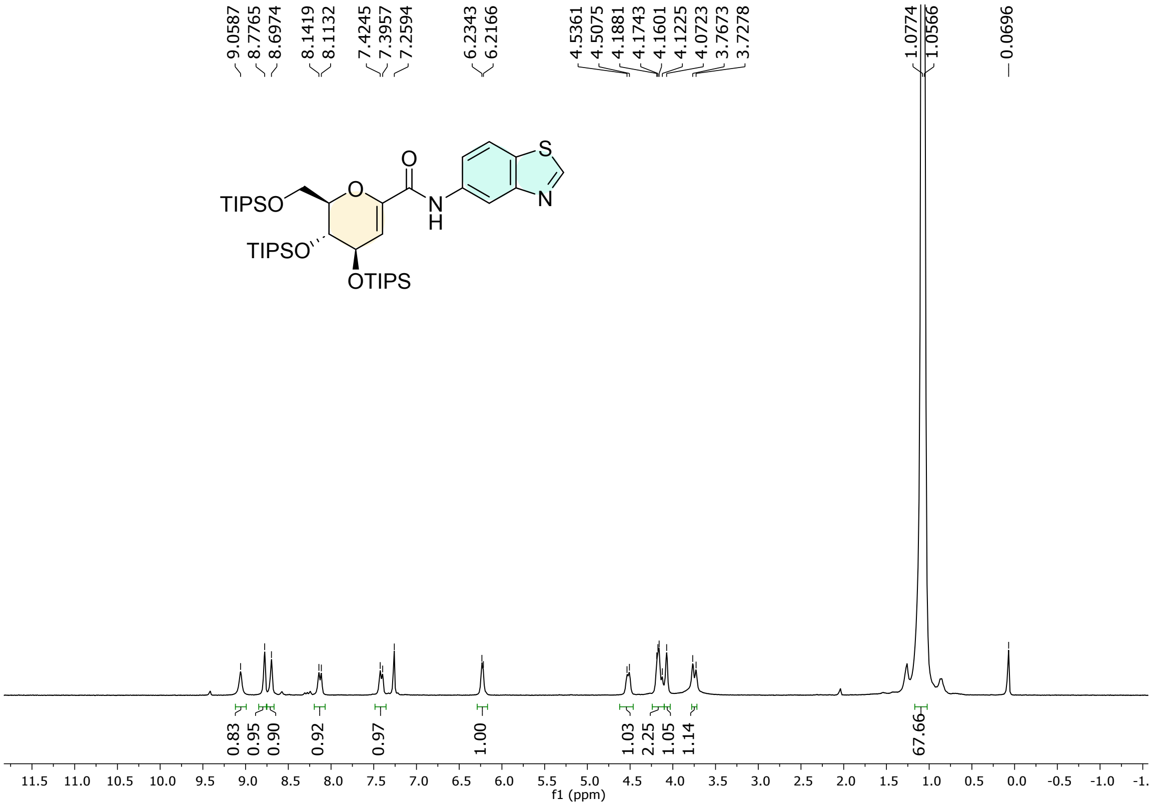

Figure S33. ^{1}H NMR spectra (300 MHz, $CDCl_3$) of **3s**

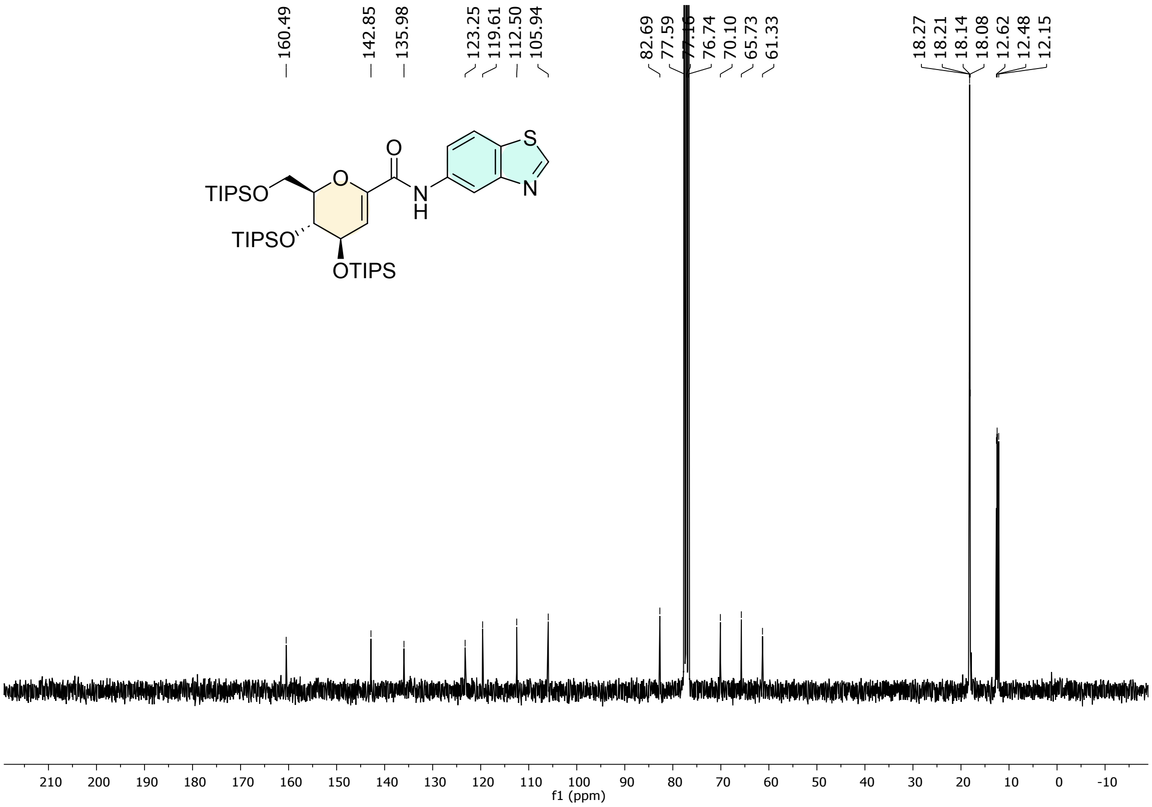

Figure S34. ^{13}C NMR spectra (75 MHz, $CDCl_3$) of **3s**

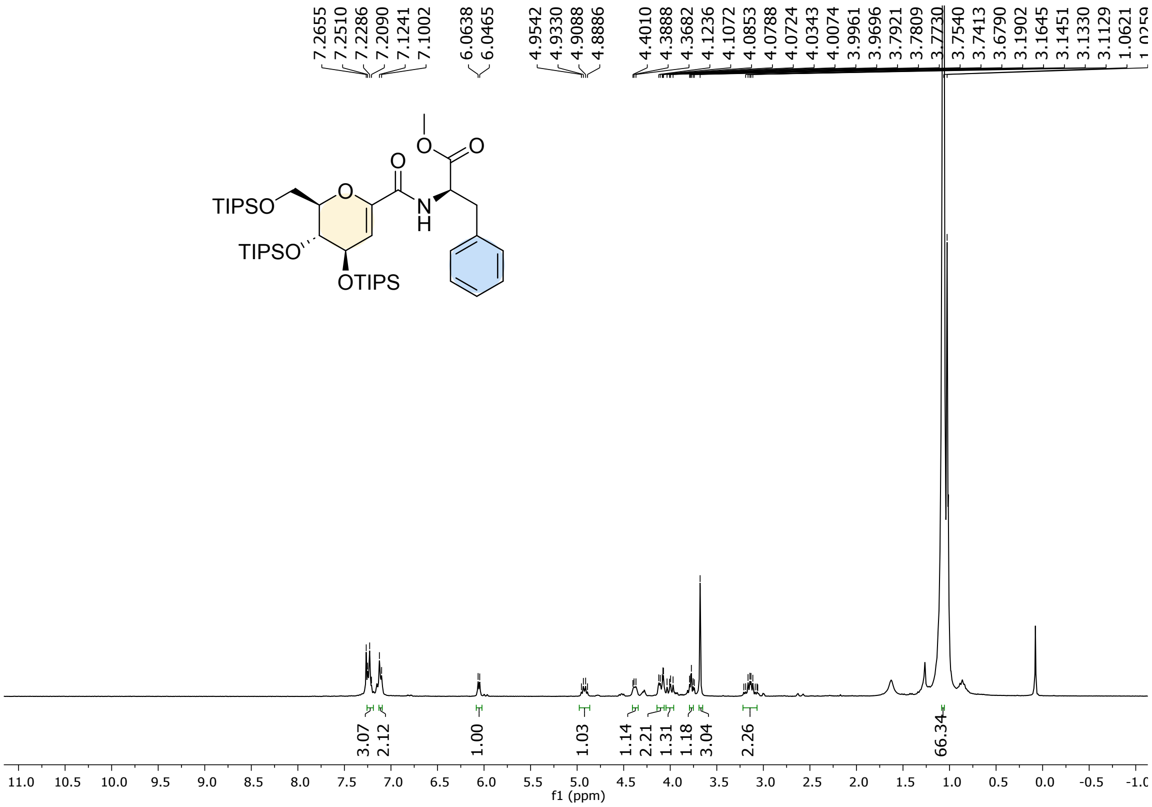

Figure S35. ^{1}H NMR spectra (300 MHz, $CDCl_3$) of **3t**

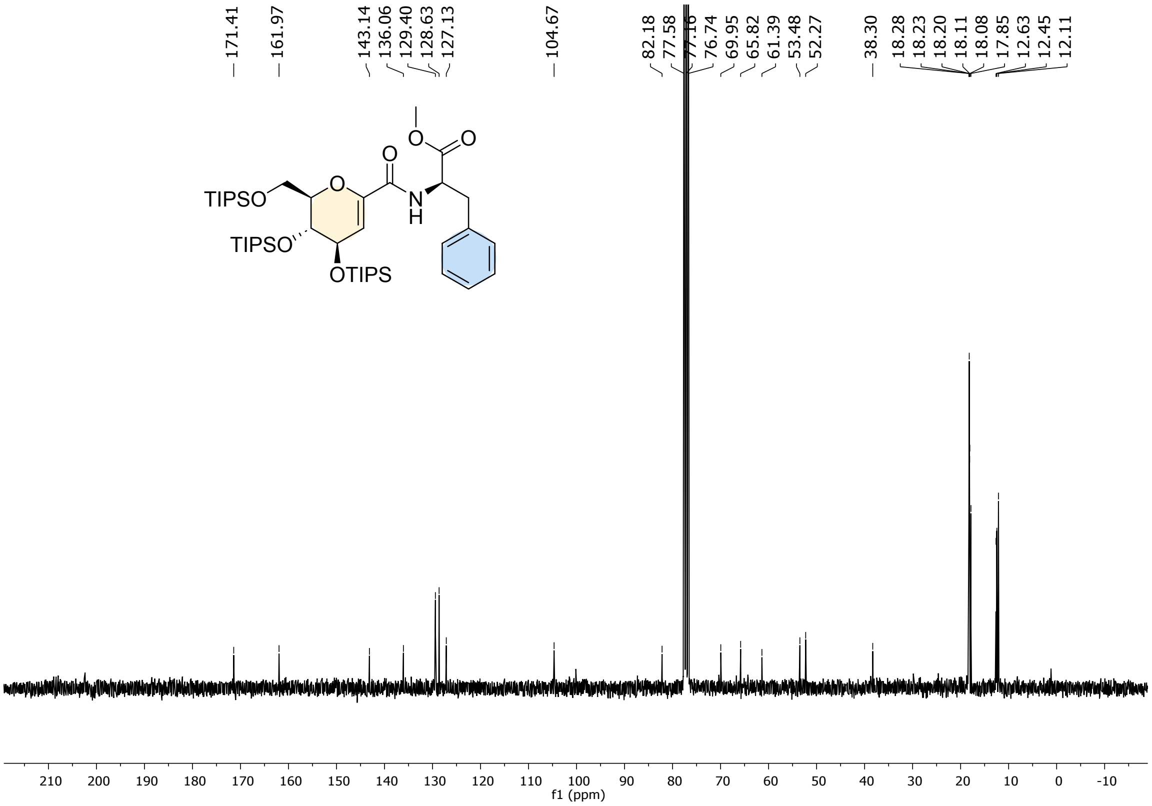

Figure S36. ^{13}C NMR spectra (75 MHz, $CDCl_3$) of **3t**

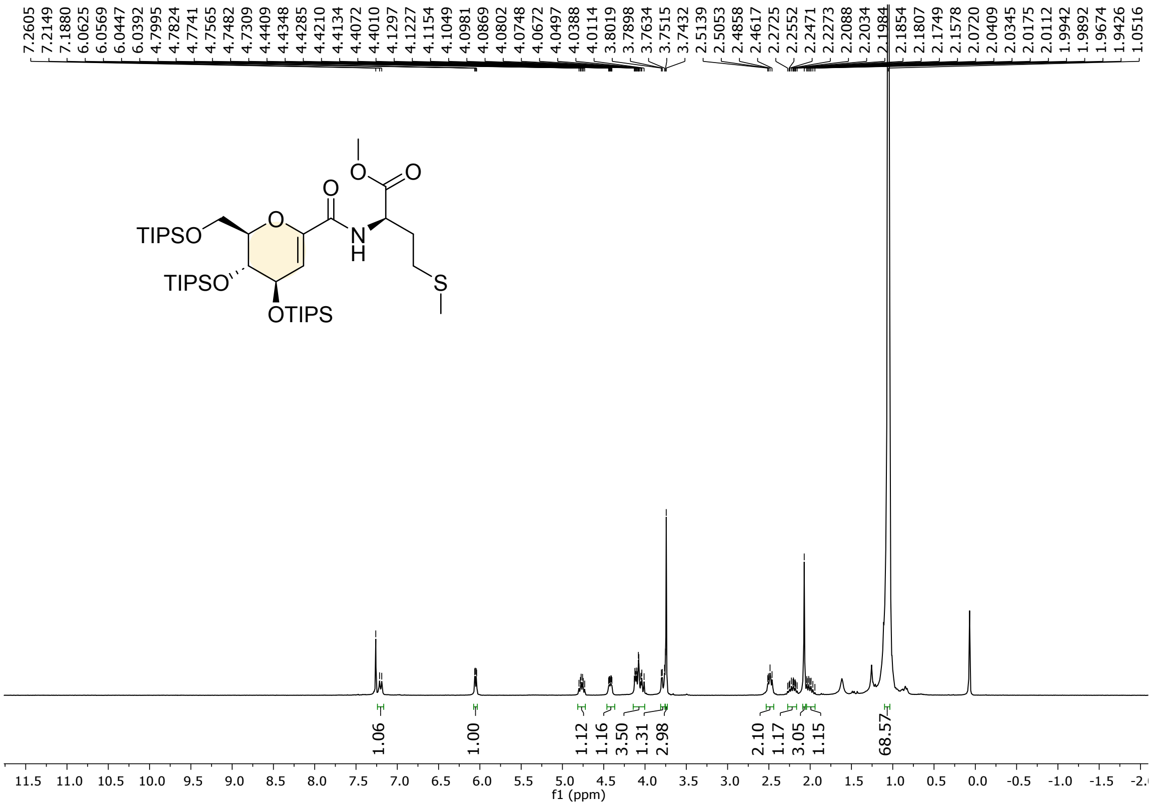

Figure S37. ^{1}H NMR spectra (300 MHz, $CDCl_3$) of **3u**

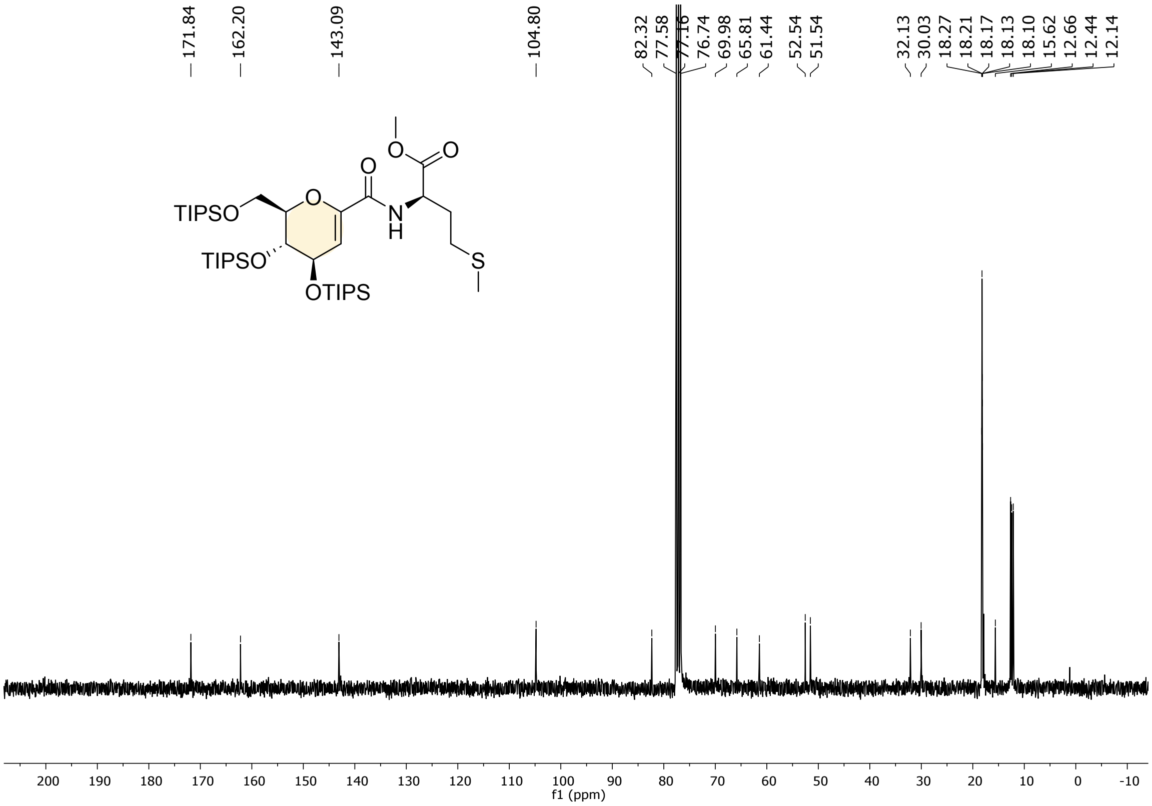

Figure S38. ^{13}C NMR spectra (75 MHz, $CDCl_3$) of **3u**

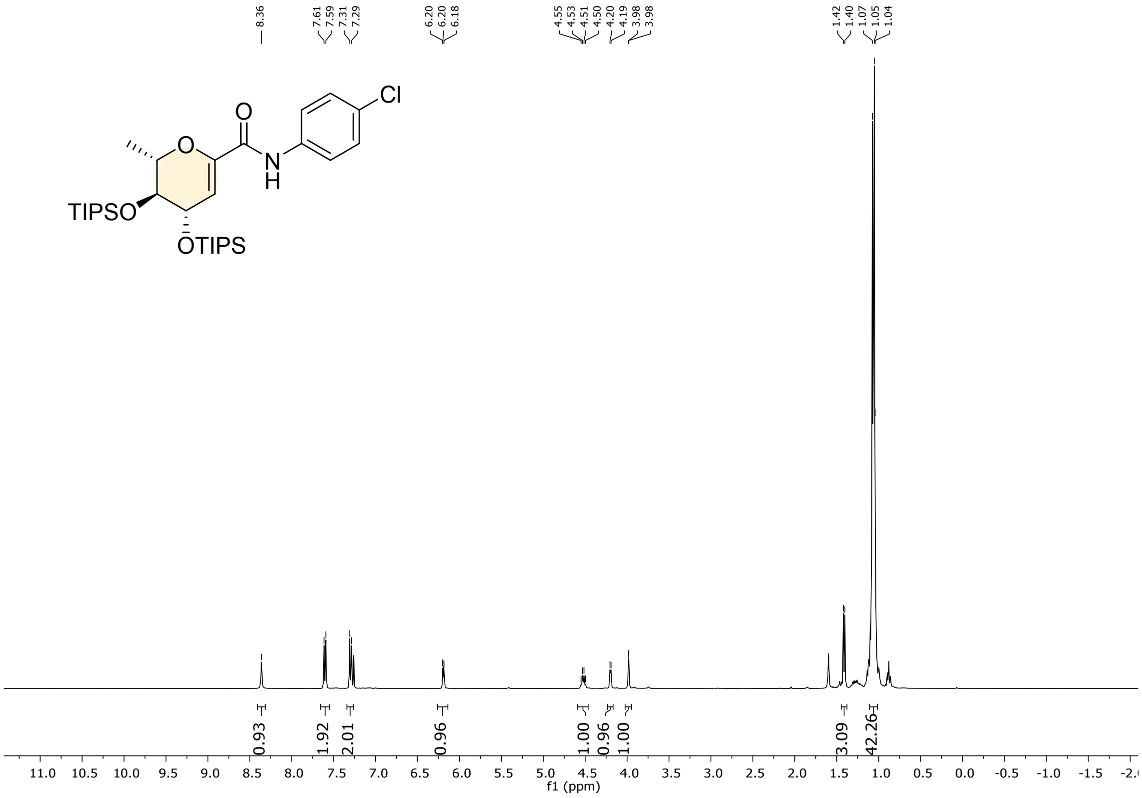

Figure S39. ^{1}H NMR spectra (400 MHz, $CDCl_3$) of **3aa**

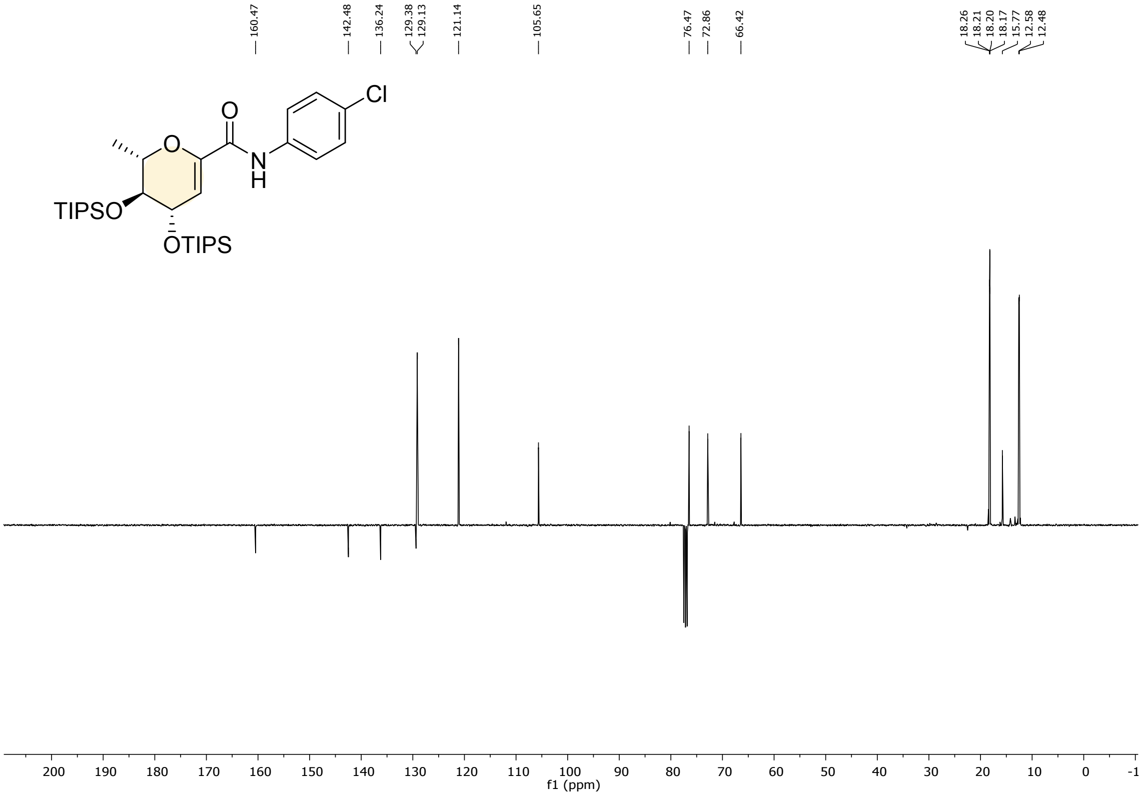

Figure S40. ^{13}C NMR spectra (101 MHz, $CDCl_3$) of **3aa**

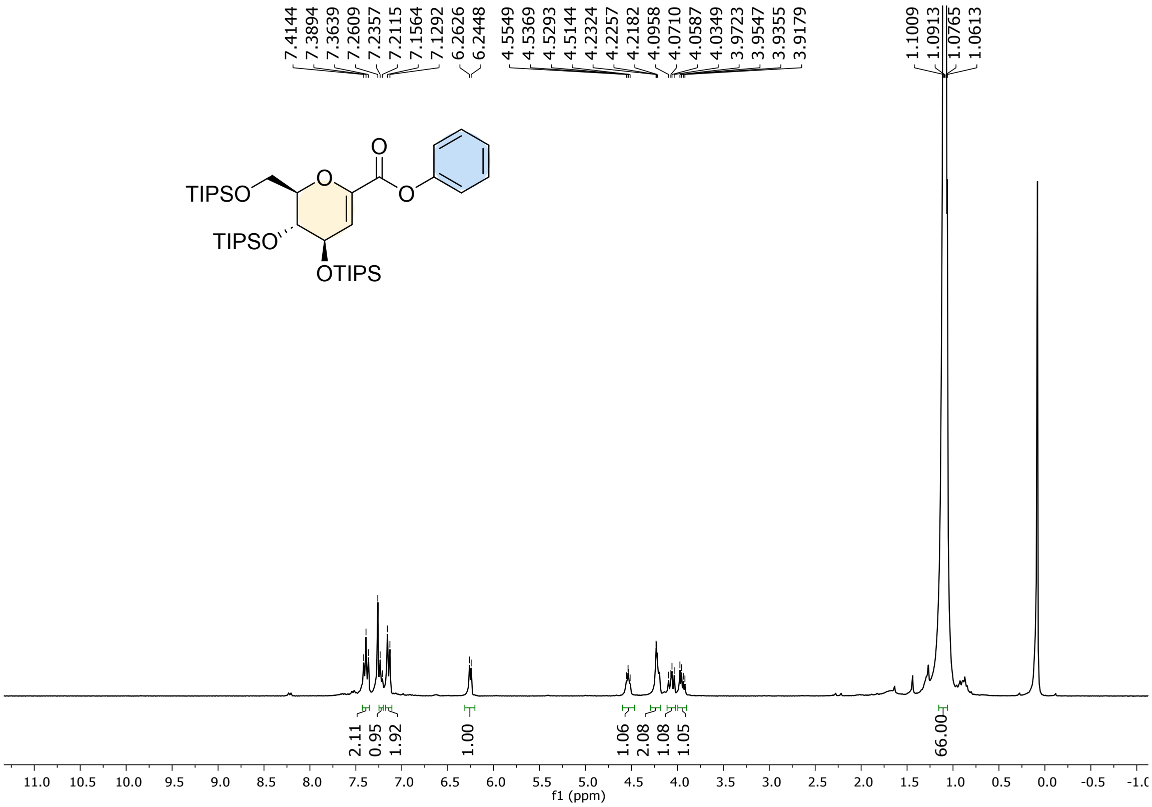

Figure S41. ^{1}H NMR spectra (300 MHz, $CDCl_3$) of **4a**

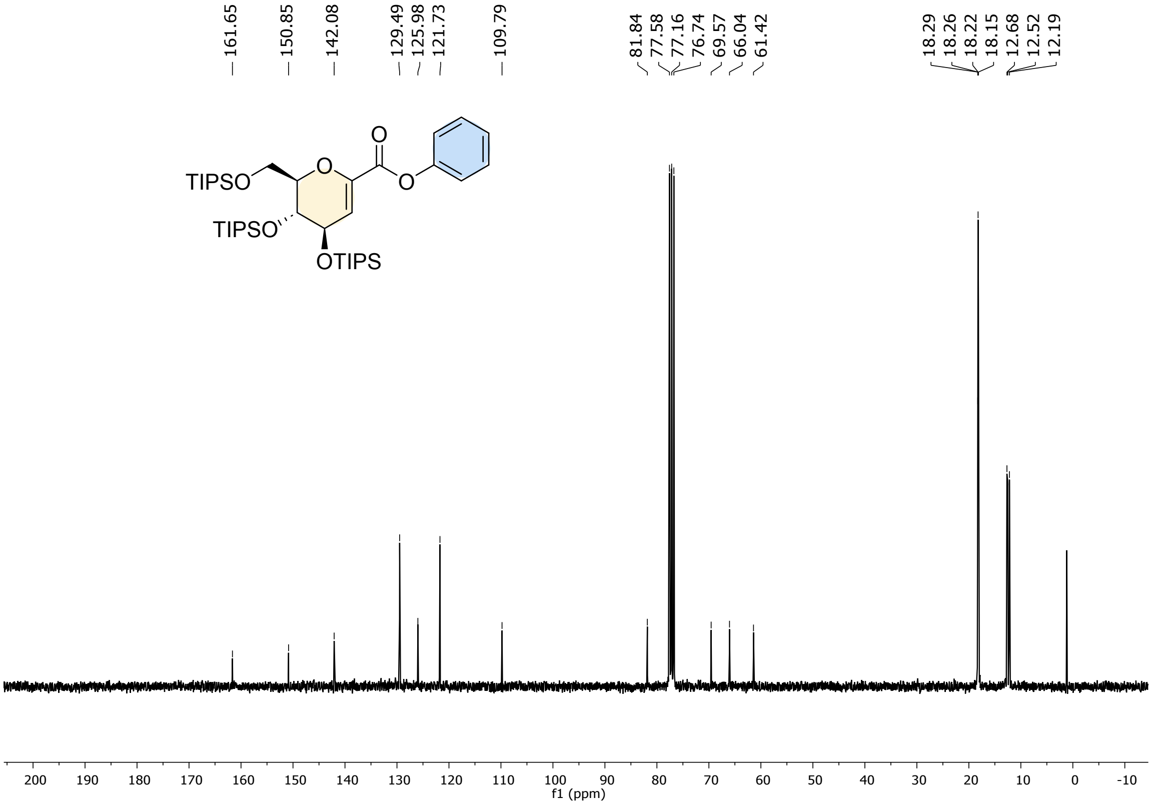

Figure S42. ^{13}C NMR spectra (75 MHz, $CDCl_3$) of **4a**

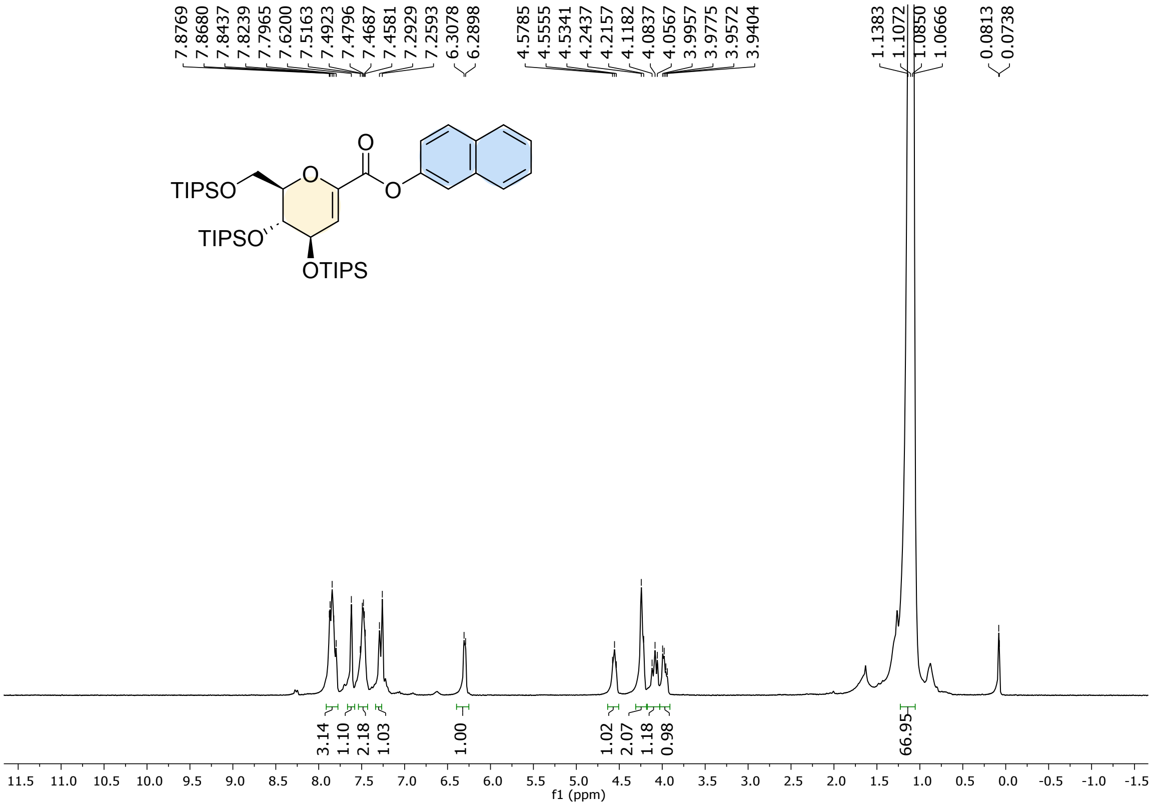

Figure S43. ^{1}H NMR spectra (300 MHz, $CDCl_3$) of **4b**

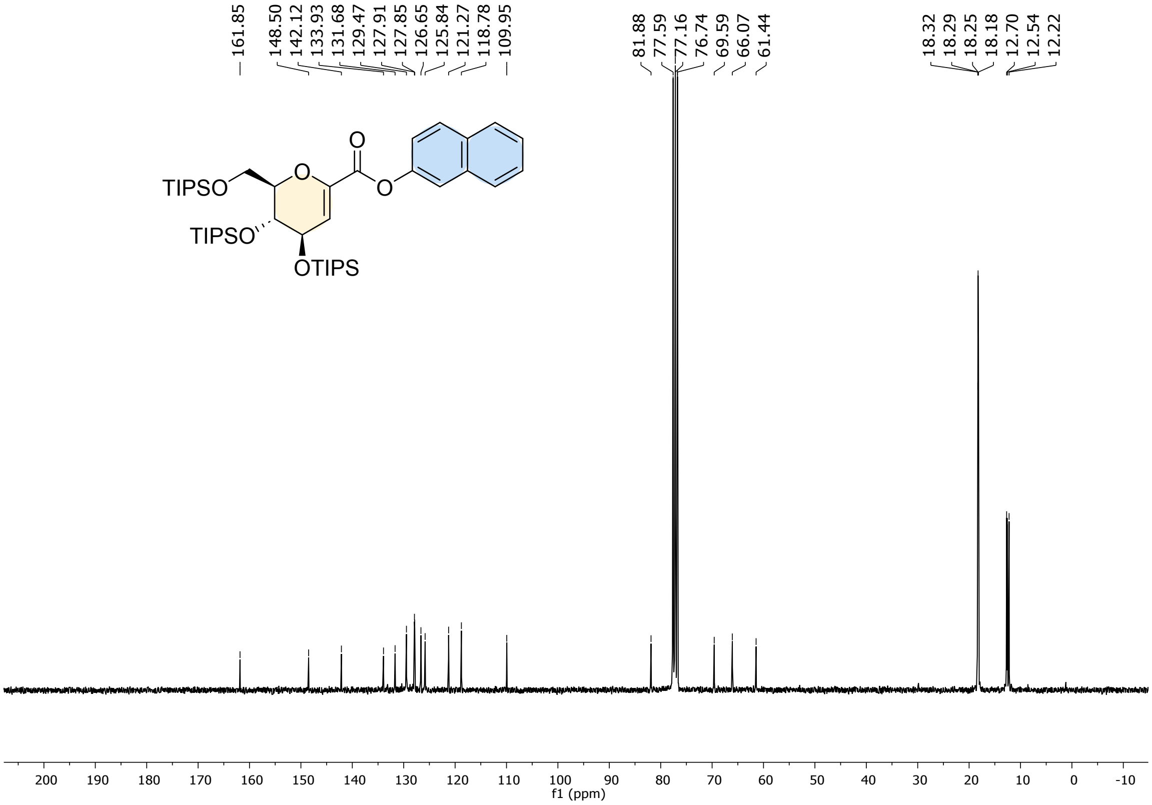

Figure S44. ^{13}C NMR spectra (75 MHz, $CDCl_3$) of **4b**

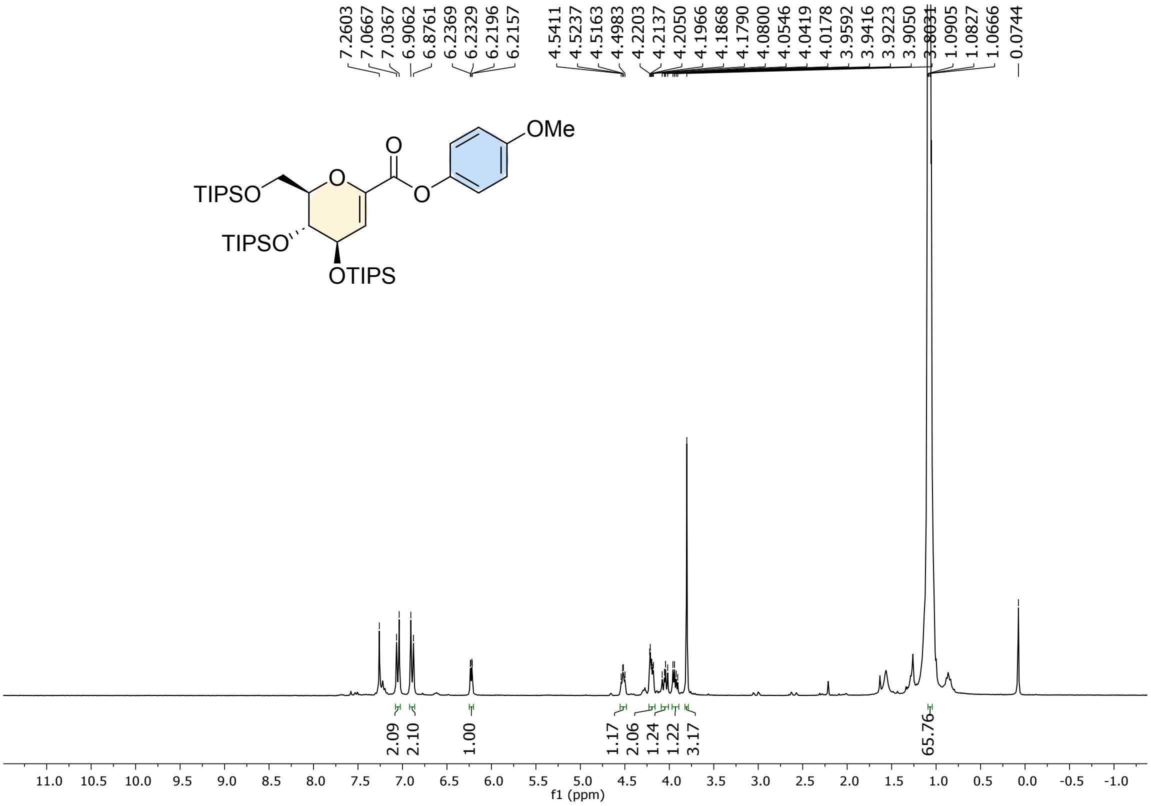

Figure S45. ^{1}H NMR spectra (300 MHz, $CDCl_3$) of **4c**

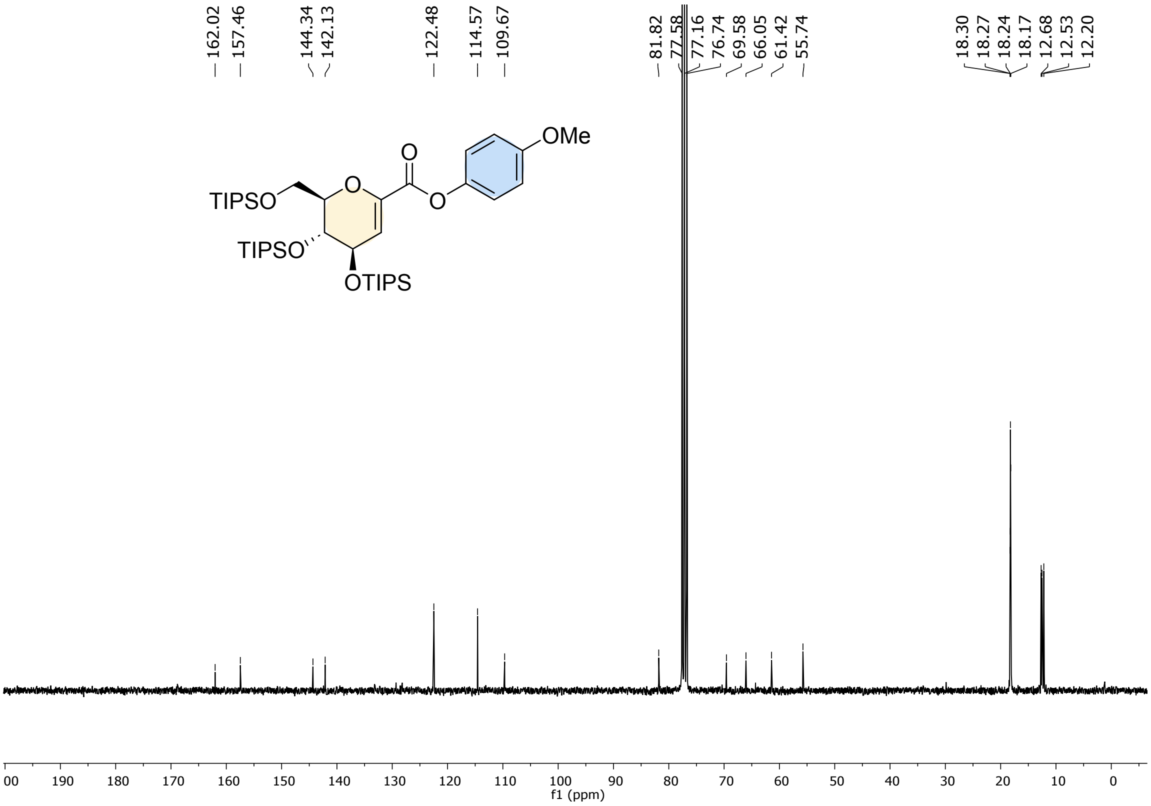

Figure S46. ^{13}C NMR spectra (75 MHz, $CDCl_3$) of **4c**

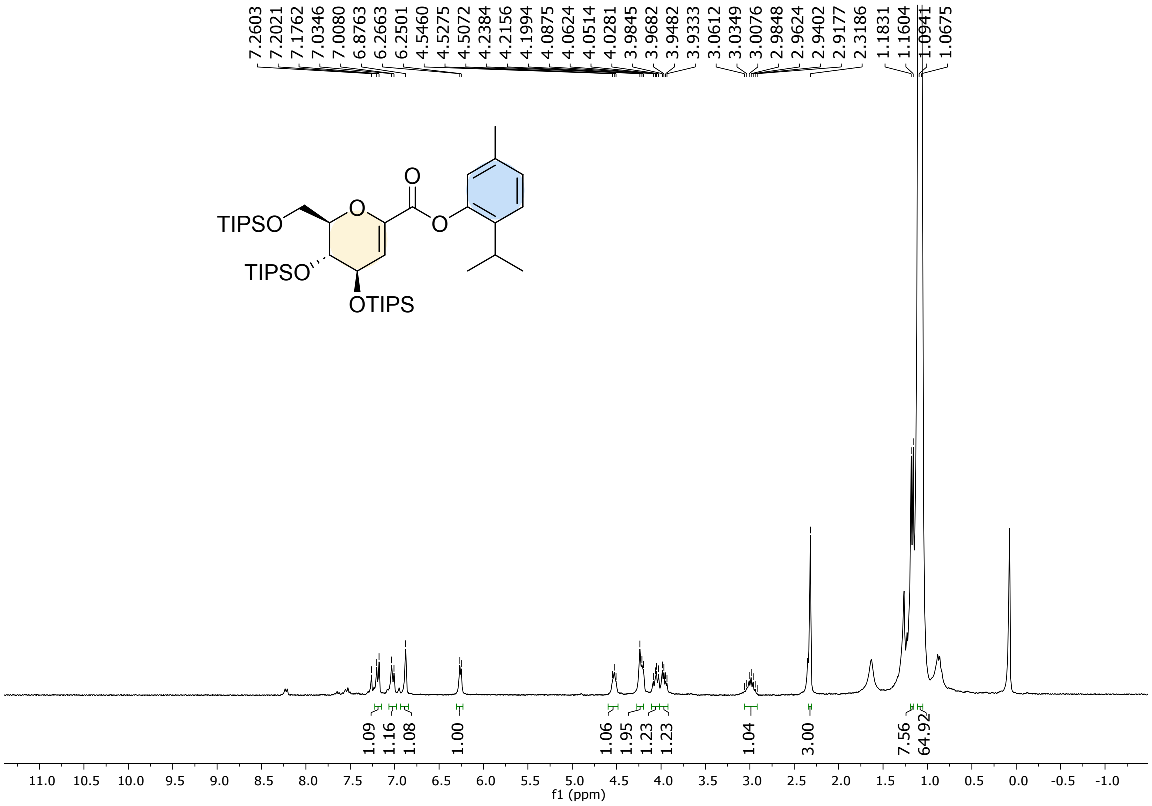

Figure S47. ^{1}H NMR spectra (300 MHz, $CDCl_3$) of **4d**

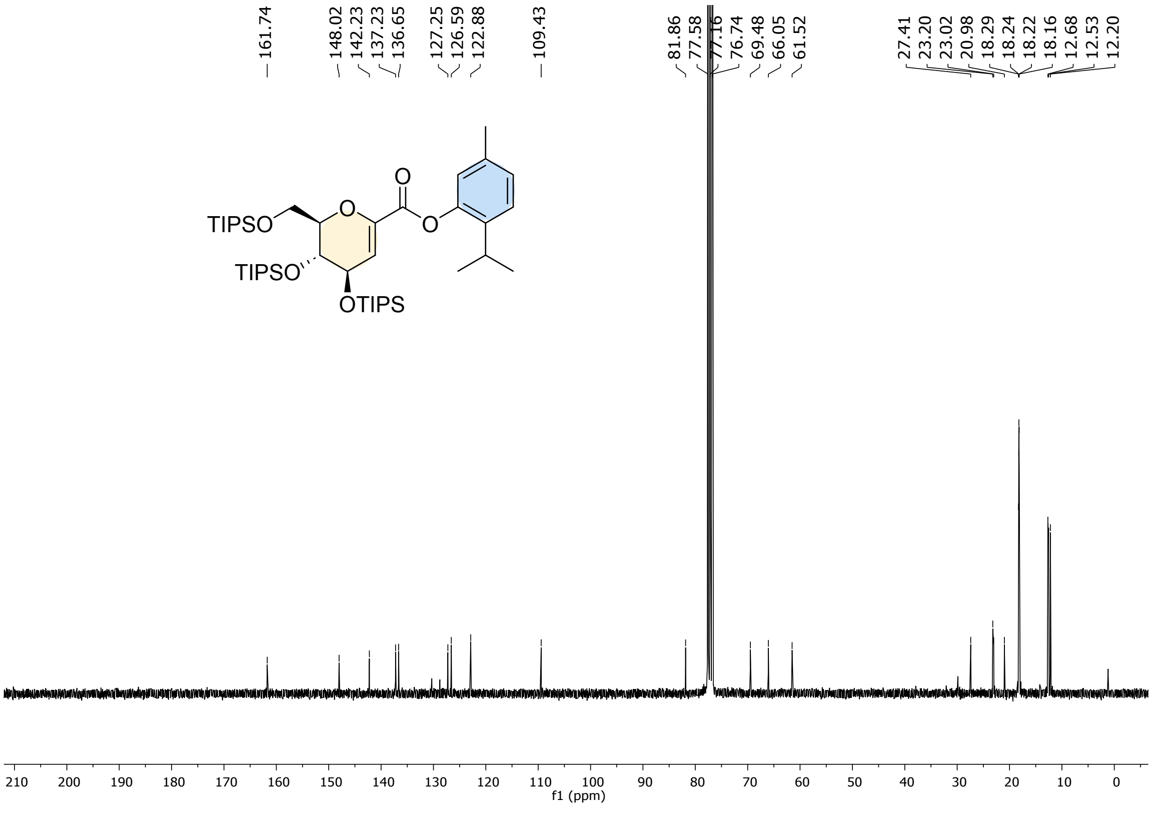

Figure S48. ^{13}C NMR spectra (75 MHz, $CDCl_3$) of **4d**

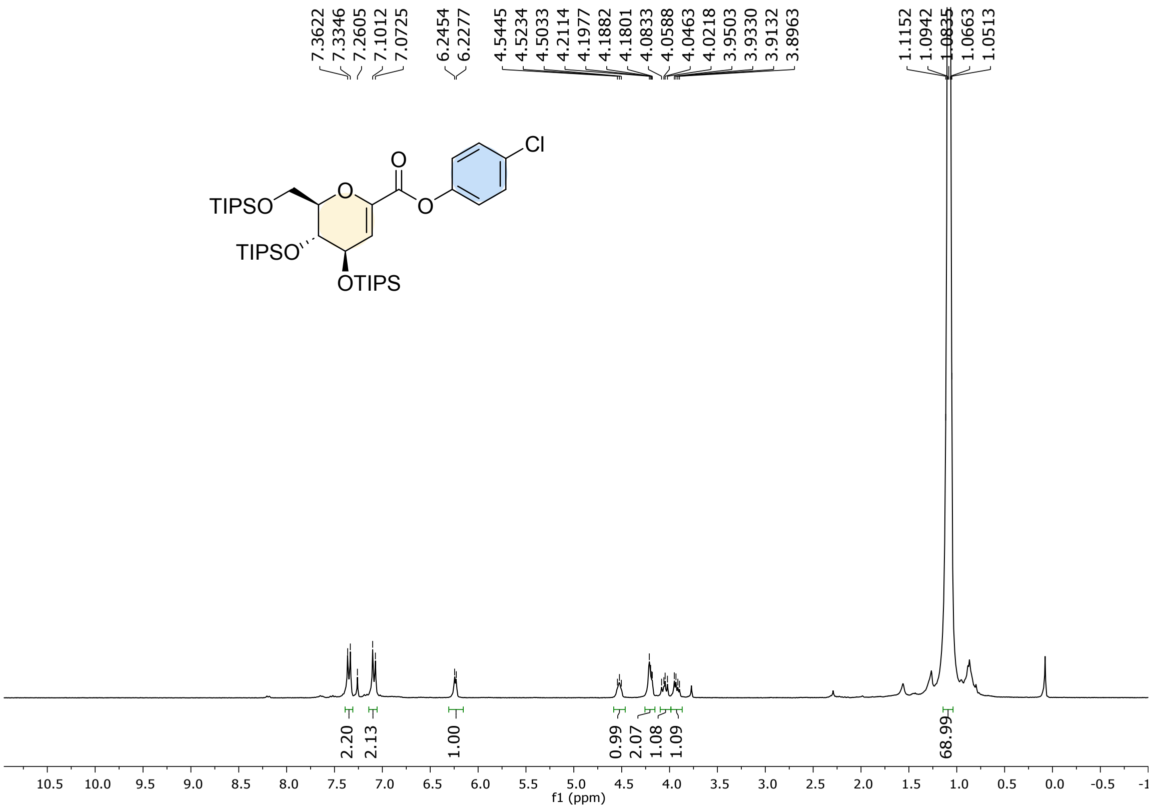

Figure S49. ^{1}H NMR spectra (300 MHz, $CDCl_3$) of **4e**

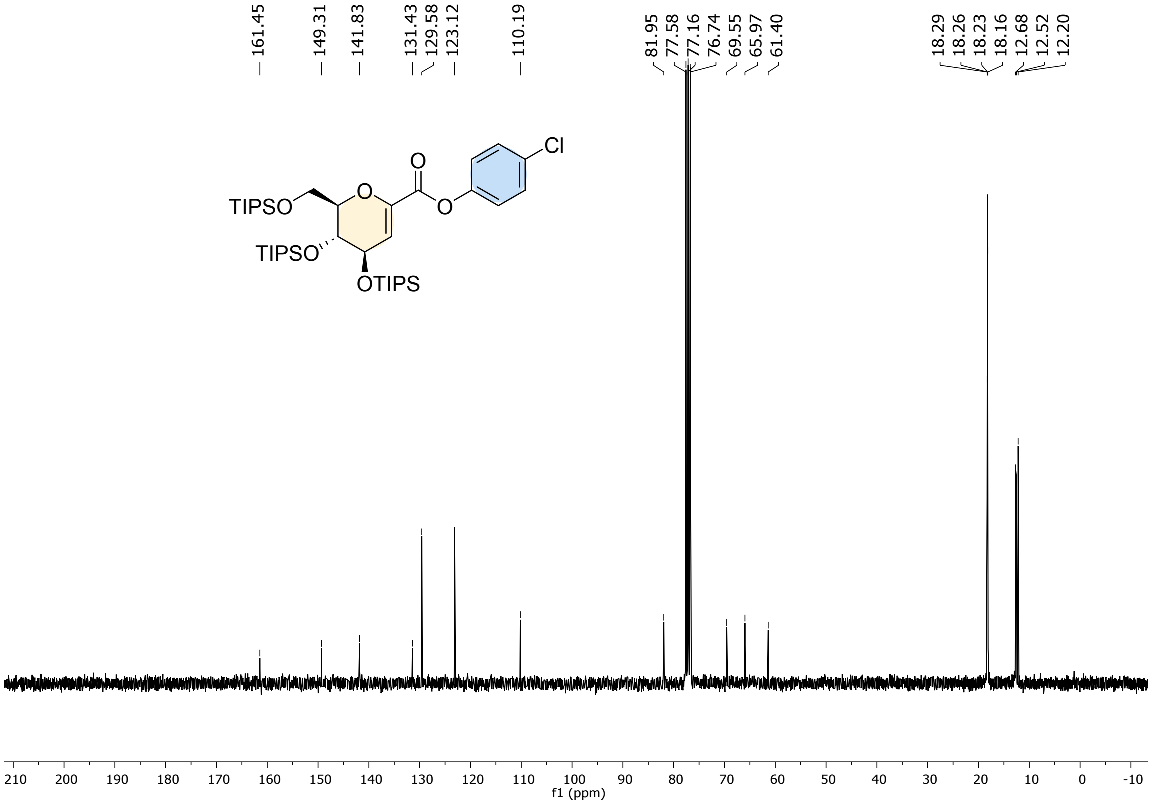

Figure S50. ^{13}C NMR spectra (75 MHz, $CDCl_3$) of **4e**

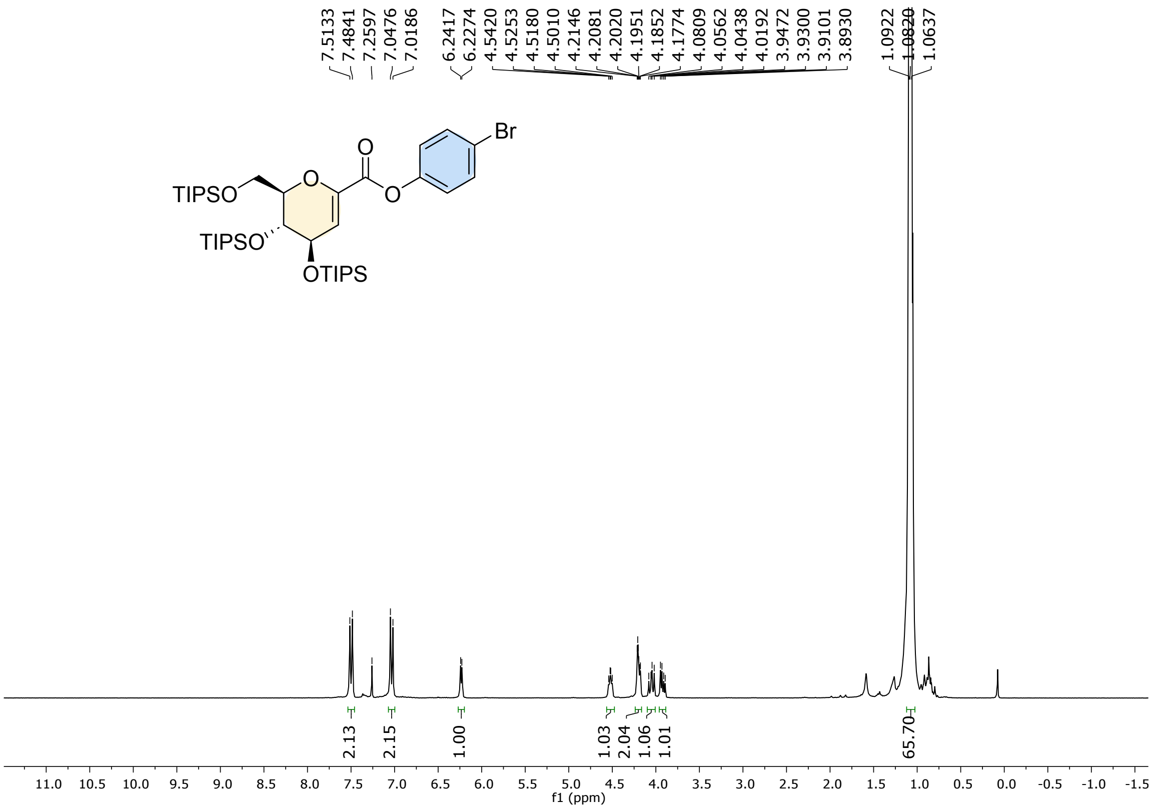

Figure S51. ^1H NMR spectra (300 MHz, $CDCl_3$) of **4f**

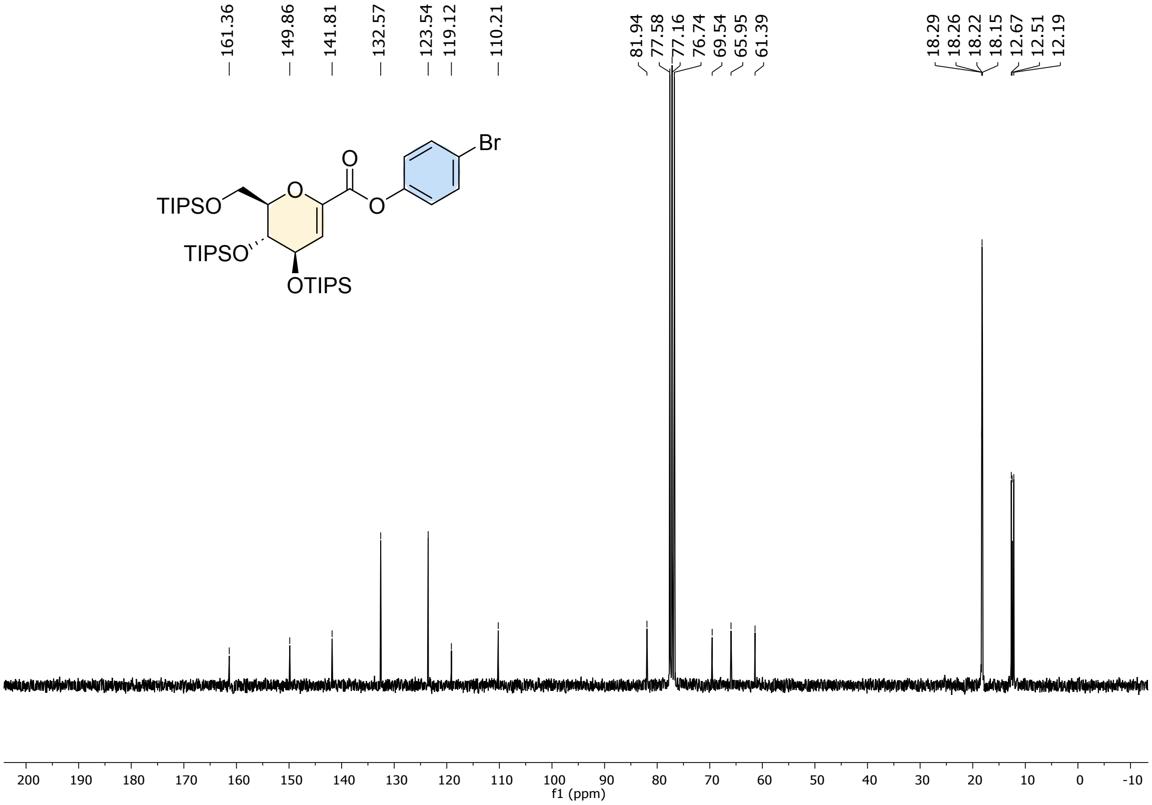

Figure S52. ^{13}C NMR spectra (75 MHz, $CDCl_3$) of **4f**

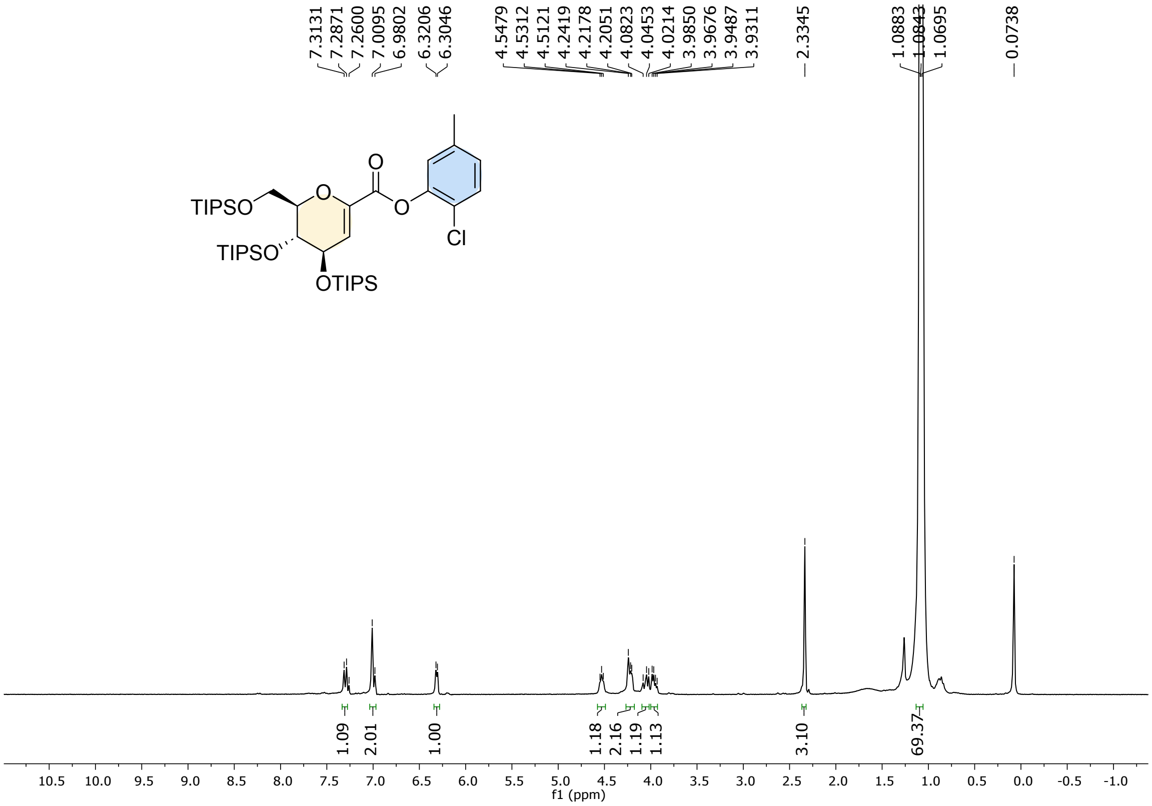

Figure S53. ^{1}H NMR spectra (300 MHz, $CDCl_3$) of **4g**

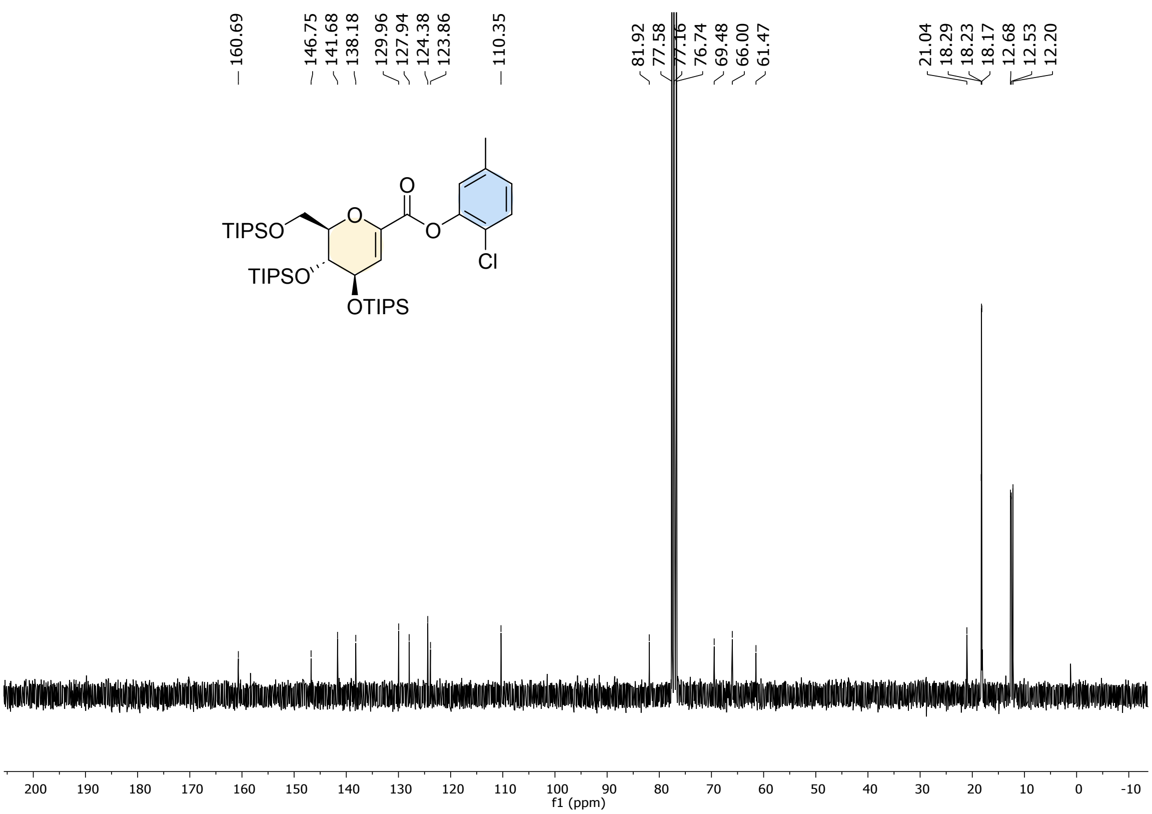

Figure S54. ^{13}C NMR spectra (75 MHz, $CDCl_3$) of **4g**

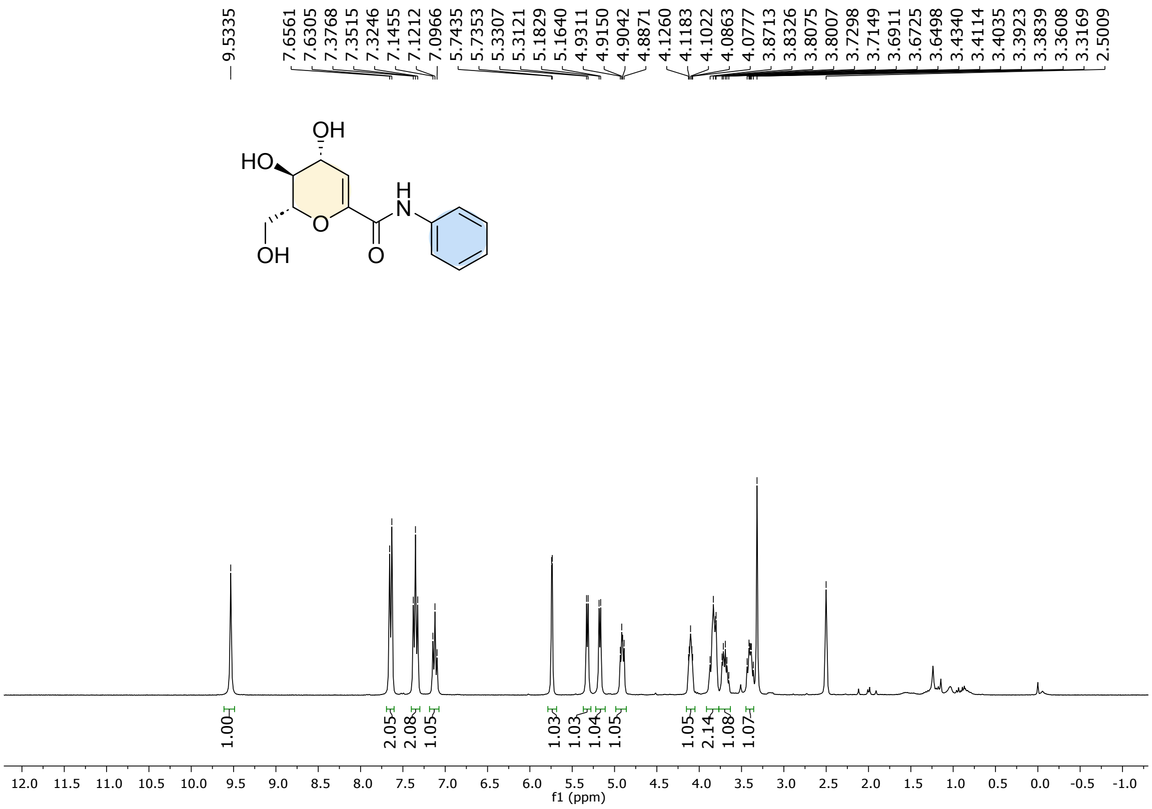

Figure S55. ^{1}H NMR spectra (300 MHz, DMSO-*d6*) of **5a**

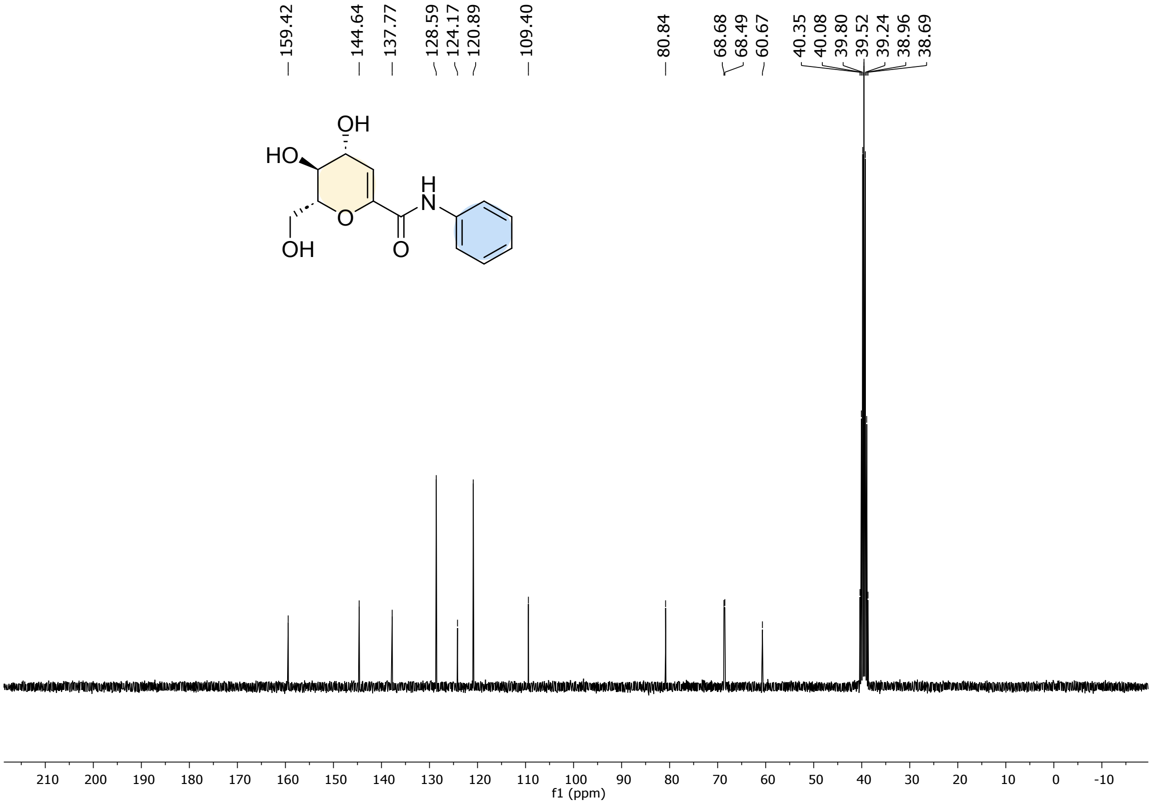

Figure S56. ^{13}C NMR spectra (75 MHz, DMSO-*d6*) of **5a**

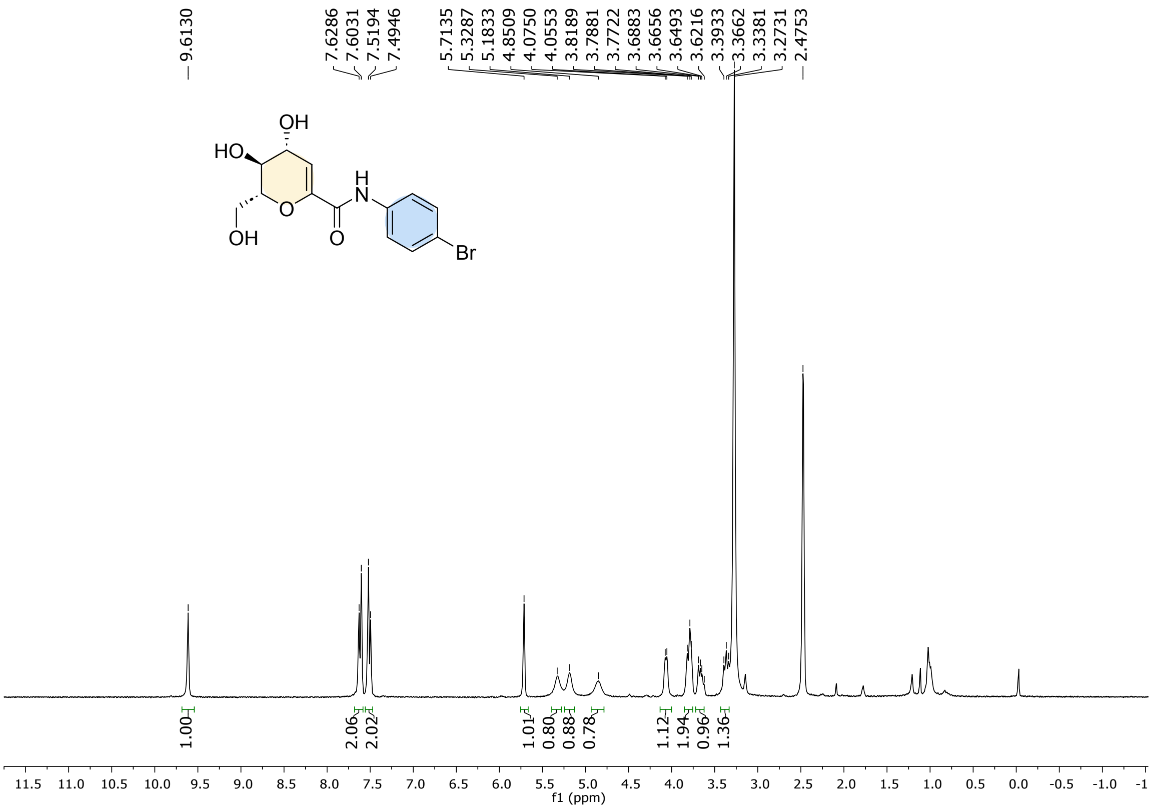

Figure S57. ^{1}H NMR spectra (300 MHz, DMSO-*d6*) of **5b**

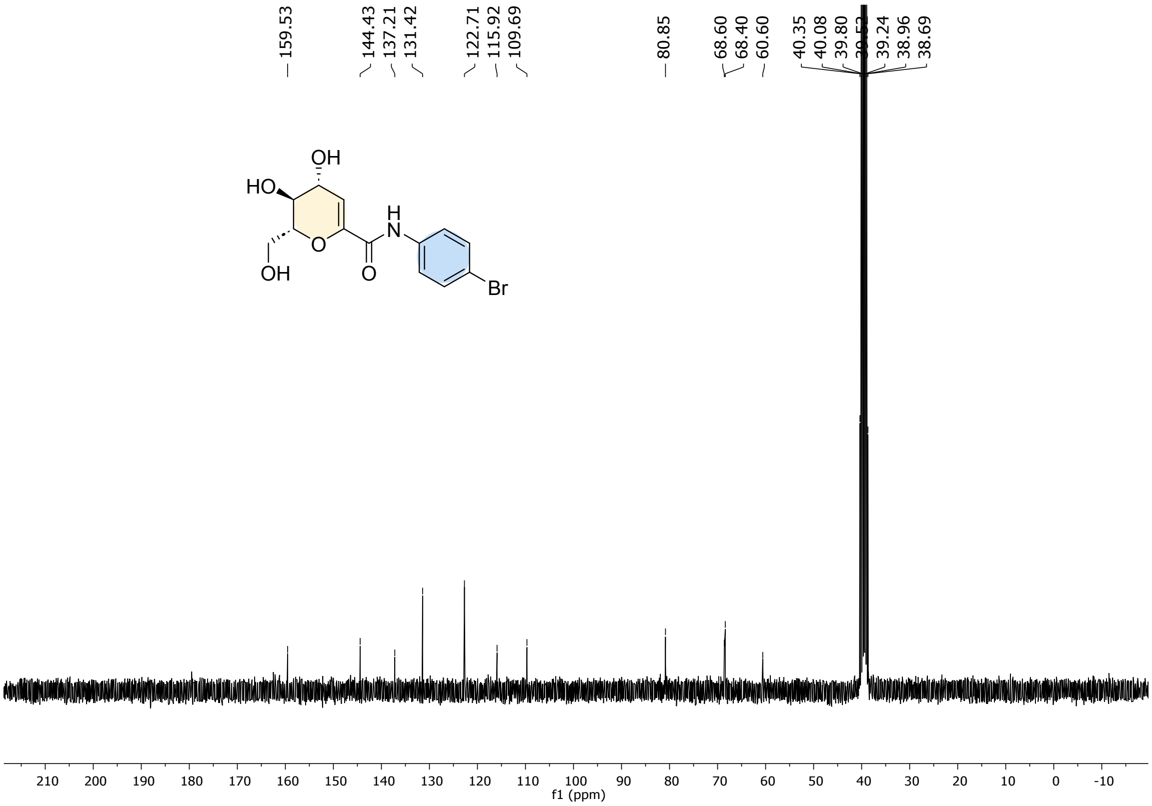

Figure S58. ^{13}C NMR spectra (75 MHz, DMSO-*d6*) of **5b**

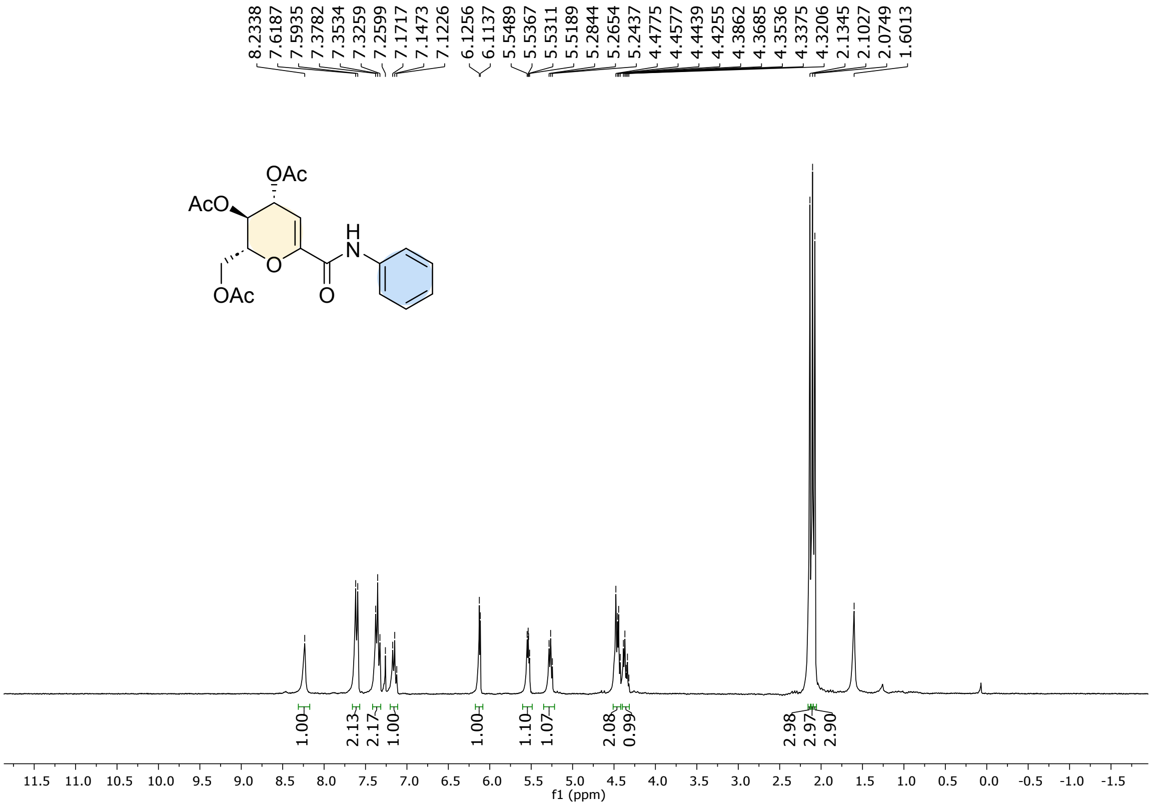

Figure S59. ^{1}H NMR spectra (300 MHz, $CDCl_3$) of **6a**

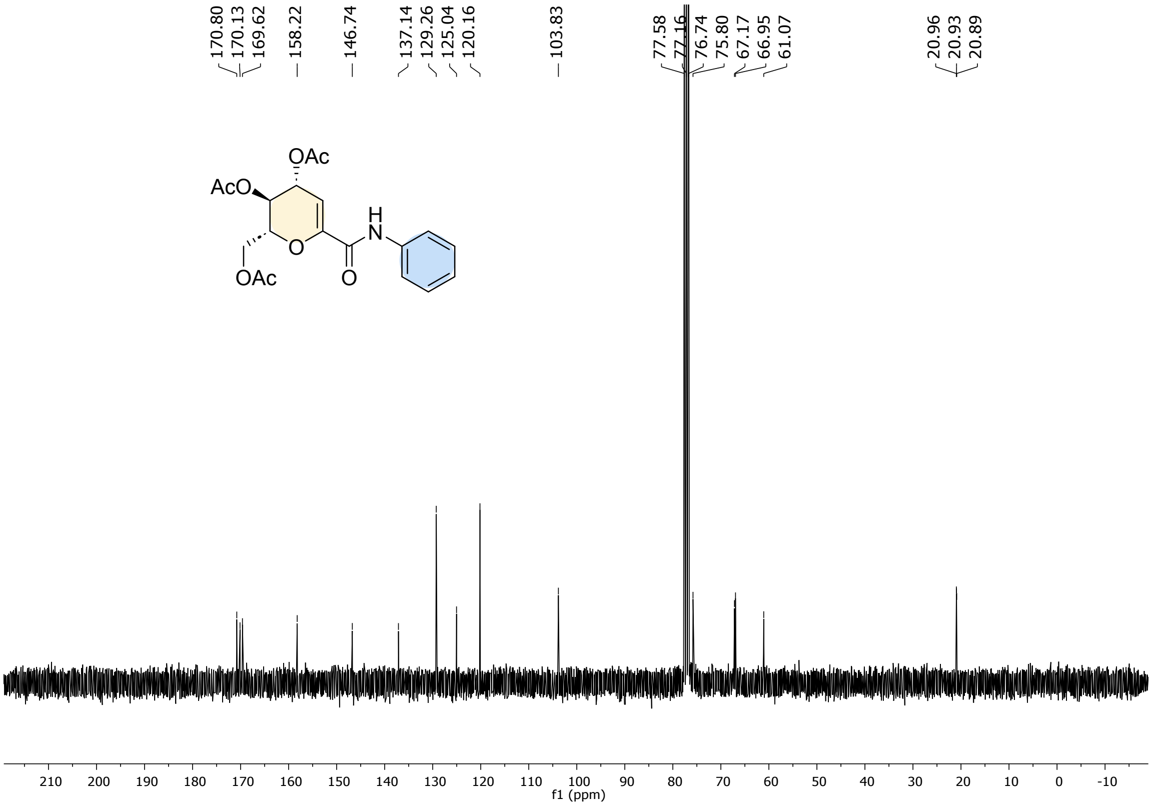

Figure S60. ^{13}C NMR spectra (75 MHz, $CDCl_3$) of **6a**

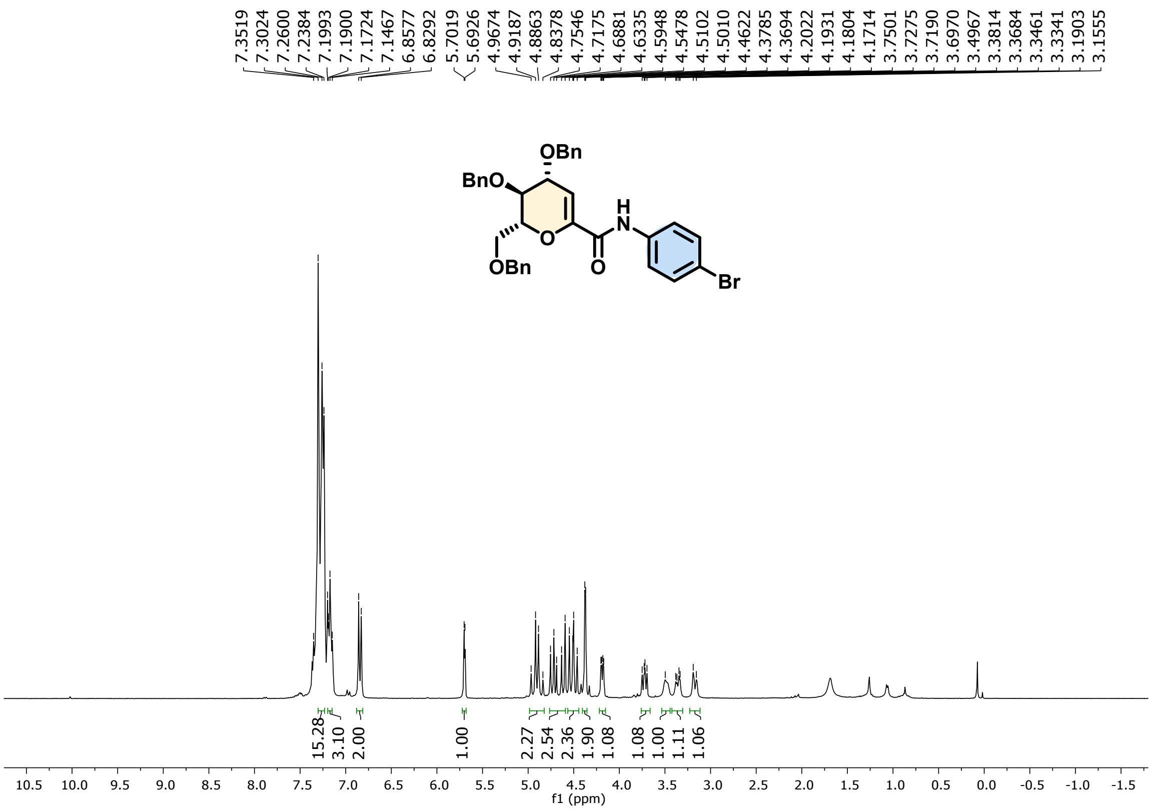

Figure S61. ^{1}H NMR spectra (300 MHz, $CDCl_3$) of **6b**

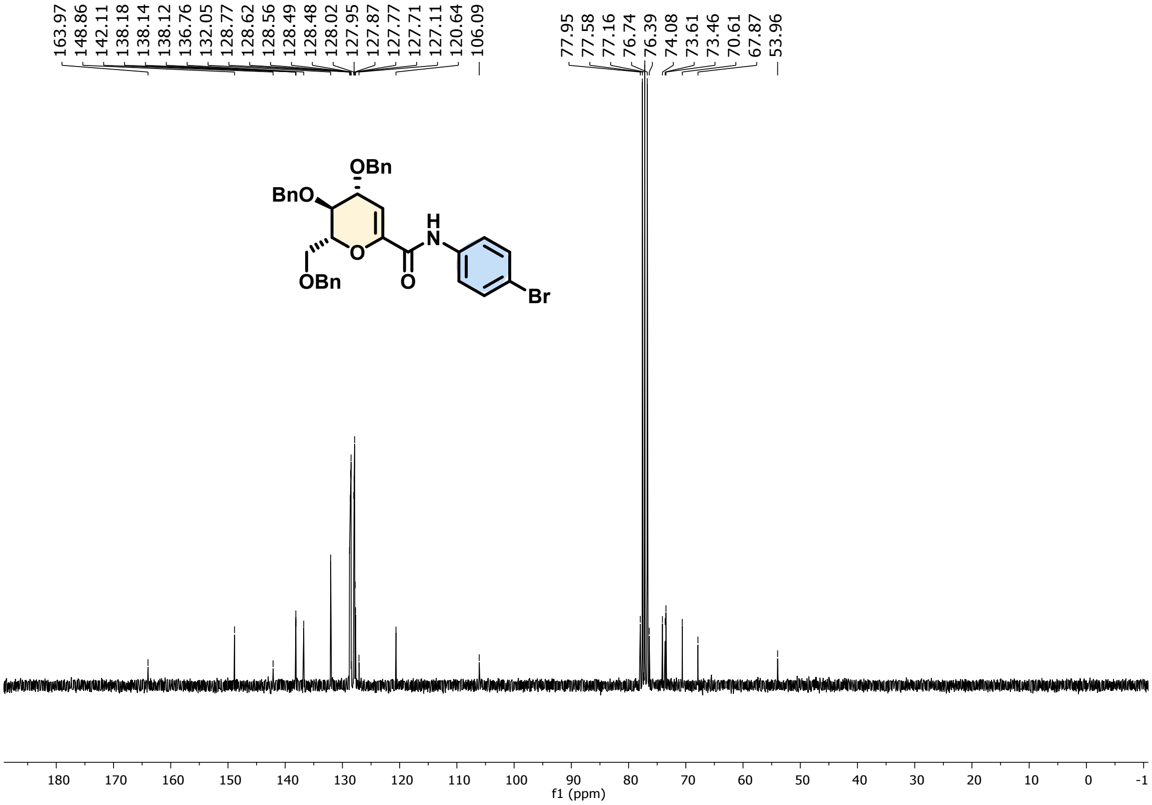

Figure S62. ^{13}C NMR spectra (75 MHz, $CDCl_3$) of **6b**

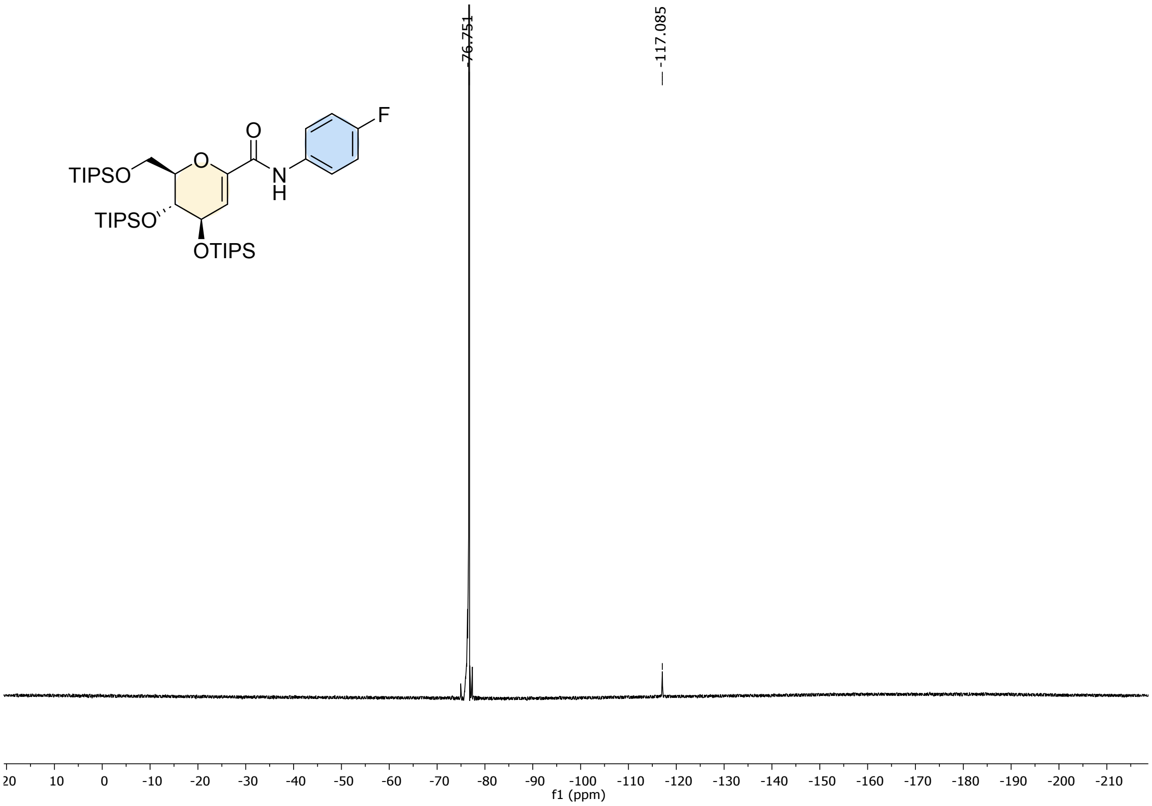

Figure S63. ^{19}F NMR spectra (282 MHz, $CDCl_3$) of **3k.** (Trifluoroacetic acid was used as an internal standard)

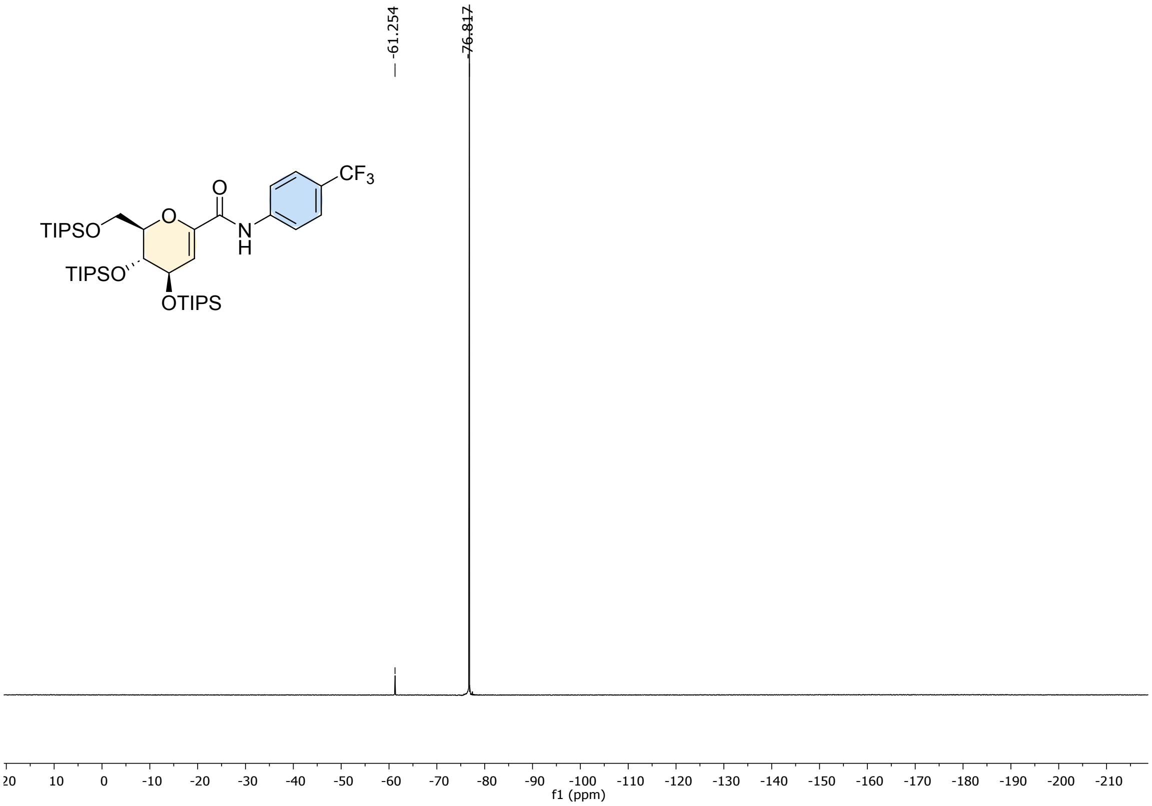

Figure S64. ^{19}F NMR spectra (282 MHz, $CDCl_3$) of **3l.** (Trifluoroacetic acid was used as an internal standard)

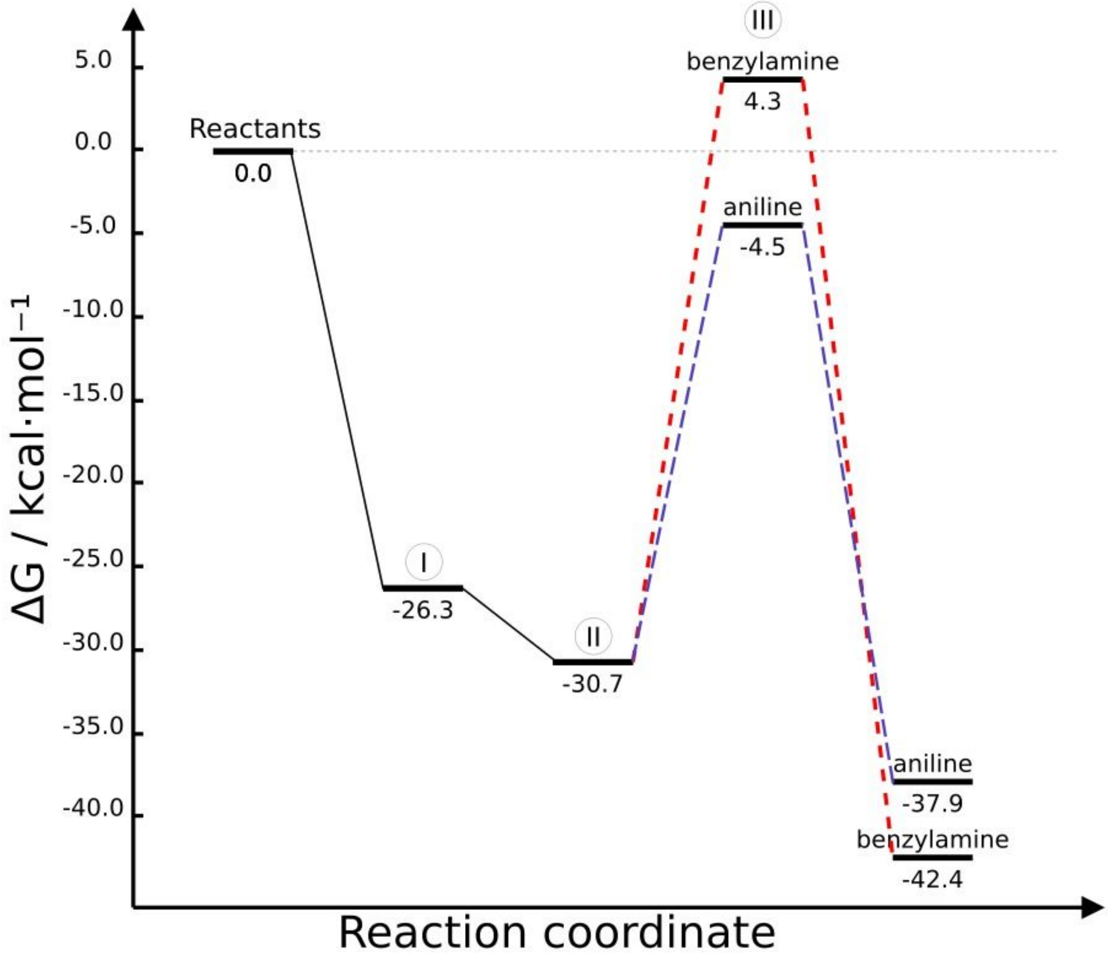

Figure S65. Potential energy surface depicting the Gibbs free energy at 298.15 K of the intermediates I, II and III portrayed in Scheme 1 for the reaction of **2a** and aniline (blue short dash) or benzylamine (red long dash) mediated by XantPhos, $Mo(CO)_6$, and Pd at the M06-2x/Def2-SVP level of theory at Gaussian16 computational package.
